# Implementing the screening for poverty and related social determinants and intervening to improve knowledge of and links to resources (SPARK) in primary care clinics across Canada

**DOI:** 10.1093/fampra/cmaf109

**Published:** 2026-04-22

**Authors:** Leanne Kosowan, Joseph J O’Rourke, Dana Howse, Lane Williams, Itunuoluwa Adekoya, Abigail Zita Seshie, Alannah Delahunty-Pike, Mélanie Ann Smithman, Alan Katz, Eunice Abaga, Alexander Zsager, Jane Cooney, Marjeiry Robinson, Dorothy Mary Senior, Kris Aubrey-Bassler, Emily Gard Marshall, Cory Neudorf, Nazeem Muhajarine, Isabelle Fortuna, Archna Gupta, Andrew D Pinto

**Affiliations:** Rady Faculty of Health Sciences, University of Manitoba, Winnipeg, Manitoba, Canada R3E 0W3; Upstream Lab, MAP Centre for Urban Health Solutions, Li Ka Shing Knowledge Institute, St. Michael’s Hospital, Unity Health Toronto, Toronto, Ontario, Canada M5B 1W8; Primary Healthcare Research Unit, Faculty of Medicine, Memorial University of Newfoundland and Labrador, St John's, Newfoundland and Labrador, Canada A1B 3V6; Department of Family Medicine, Dalhousie University, Halifax, Nova Scotia, Canada B3H 4R2; Upstream Lab, MAP Centre for Urban Health Solutions, Li Ka Shing Knowledge Institute, St. Michael’s Hospital, Unity Health Toronto, Toronto, Ontario, Canada M5B 1W8; Upstream Lab, MAP Centre for Urban Health Solutions, Li Ka Shing Knowledge Institute, St. Michael’s Hospital, Unity Health Toronto, Toronto, Ontario, Canada M5B 1W8; Faculty of Health, Dalhousie University, Halifax, Nova Scotia, Canada B3H 4R2; Upstream Lab, MAP Centre for Urban Health Solutions, Li Ka Shing Knowledge Institute, St. Michael’s Hospital, Unity Health Toronto, Toronto, Ontario, Canada M5B 1W8; Rady Faculty of Health Sciences, University of Manitoba, Winnipeg, Manitoba, Canada R3E 0W3; Upstream Lab, MAP Centre for Urban Health Solutions, Li Ka Shing Knowledge Institute, St. Michael’s Hospital, Unity Health Toronto, Toronto, Ontario, Canada M5B 1W8; Upstream Lab, MAP Centre for Urban Health Solutions, Li Ka Shing Knowledge Institute, St. Michael’s Hospital, Unity Health Toronto, Toronto, Ontario, Canada M5B 1W8; Upstream Lab, MAP Centre for Urban Health Solutions, Li Ka Shing Knowledge Institute, St. Michael’s Hospital, Unity Health Toronto, Toronto, Ontario, Canada M5B 1W8; Upstream Lab, MAP Centre for Urban Health Solutions, Li Ka Shing Knowledge Institute, St. Michael’s Hospital, Unity Health Toronto, Toronto, Ontario, Canada M5B 1W8; Upstream Lab, MAP Centre for Urban Health Solutions, Li Ka Shing Knowledge Institute, St. Michael’s Hospital, Unity Health Toronto, Toronto, Ontario, Canada M5B 1W8; Primary Healthcare Research Unit, Faculty of Medicine, Memorial University of Newfoundland and Labrador, St John's, Newfoundland and Labrador, Canada A1B 3V6; Faculty of Medicine, Memorial University of Newfoundland and Labrador, St John's, Newfoundland and Labrador, Canada A1B 3X5; Department of Family Medicine, Dalhousie University, Halifax, Nova Scotia, Canada B3H 4R2; Department of Community Health and Epidemiology, College of Medicine, University of Saskatchewan, Saskatoon, Saskatchewan, Canada S7N 5E5; Saskatchewan Population Health Evaluation Research Unit, Saskatoon, Saskatchewan, Canada S7N 2Z4; Department of Community Health and Epidemiology, College of Medicine, University of Saskatchewan, Saskatoon, Saskatchewan, Canada S7N 5E5; Saskatchewan Population Health Evaluation Research Unit, Saskatoon, Saskatchewan, Canada S7N 2Z4; Upstream Lab, MAP Centre for Urban Health Solutions, Li Ka Shing Knowledge Institute, St. Michael’s Hospital, Unity Health Toronto, Toronto, Ontario, Canada M5B 1W8; Upstream Lab, MAP Centre for Urban Health Solutions, Li Ka Shing Knowledge Institute, St. Michael’s Hospital, Unity Health Toronto, Toronto, Ontario, Canada M5B 1W8; Department of Family and Community Medicine, St. Michael's Hospital, Unity Health Toronto, Toronto, Ontario, Canada M5B 1W8; Department of Family and Community Medicine, Faculty of Medicine, University of Toronto, Toronto, Ontario, Canada M5G 1V7; Department of Family and Community Medicine, Faculty of Medicine, University of Toronto, Toronto, Ontario, Canada M5G 1V7; Upstream Lab, MAP Centre for Urban Health Solutions, Li Ka Shing Knowledge Institute, St. Michael’s Hospital, Unity Health Toronto, Toronto, Ontario, Canada M5B 1W8; Department of Family and Community Medicine, St. Michael's Hospital, Unity Health Toronto, Toronto, Ontario, Canada M5B 1W8; Dalla Lana School of Public Health, University of Toronto, Toronto, Ontario, Canada M5T 3M7

**Keywords:** social determinants of health, primary health care, electronic health record, social conditions, health service research, patient-centered care

## Abstract

**Background:**

Routine systematic collection of social determinants data can inform improvements to health service delivery and system and policy planning.

**Objective:**

This cohort study reports on the implementation of the SPARK tool to collect and integrate demographic and social needs data in primary care.

**Methods:**

The 20-question SPARK tool was implemented from September 2022 to October 2023 at five primary care clinics. Implementation was evaluated using the following outcomes: acceptability, appropriateness, adoption, feasibility, fidelity, cost, penetration, and sustainability. Data included: clinic checklists (*n* = 5), training evaluations (*n* = 33), SPARK tool responses (*n* = 2063), patient surveys (*n* = 1368), and provider and staff surveys (*n* = 36). Survey responses were analyzed using frequency (%) and mean (SD). Open-ended responses were analyzed to identify key themes.

**Results:**

There was strong acceptability and adoption of the tool. Collection methods contributed to variation in perceived relevance, compatibility and barriers. Among patients, 90.5% indicated the tool was clear and easy to complete, and 84.5% had a positive experience. A minority of patients (7.8%) indicated difficulty answering ≥1 question on SPARK. Among clinic staff and providers, 96.7% reported the tool was useful, and 81.8% had a positive experience. Clinics highlighted concerns with resource allocation and suggested adjustments to collection, integration, and personnel to support implementation.

**Conclusions:**

The SPARK tool is acceptable and feasible for adoption in primary care clinics. This study provides practical insight into implementing demographic and social needs data collection, including adaptations to clinic workflows, enhanced integration platforms, and supports that ensure the sustainability of social needs screening in primary care.

Key messages

- The SPARK tool supports collection of sociodemographic data in primary care
- Exploring implementation informs collection, integration, and use of data
- Collaboratively designed context-specific workflows support implementation
- Collection of sociodemographic data supports intervening on health inequities

## Introduction

The social determinants of health are interrelated social and economic factors including socioeconomic status (income, education, and employment), social and physical environments (housing, ethnicity, residence area, and social capital), as well as surrounding structural and societal factors that contribute to health inequalities [1, 2]. The socioeconomic gradient in health is well described, impacting health outcomes and contributing to growing recognition of health disparities and the need for interventions to consider social and structural context [1–4]. Health services delivered to address health inequalities must consider mechanisms and associations that impact health behaviors, the ability to attend to health issues, and access services, as well as the response to interventions [1–6].

Within Canada, primary care is often an individual's first point of contact with the healthcare system, with a range of services including preventive, chronic disease management, and acute care. Primary care presents an opportunity to coordinate clinical care, public health, and community-based social services [1]. However, the primary healthcare system is challenged to deliver health services while considering associated social complexity [1, 2, 6]. Concerns regarding remuneration, service provision, knowledge of patient social context, and resources and tools to support social and economic needs still persist [1]. Despite concerns, patients and providers report comfort with the capture of social and demographic data within primary care clinics [5, 7–9]. In fact, screening for social needs has been shown to improve the patient-provider relationship [9, 10]. Nationally, there is no standardized approach to collection of social determinants of health data. While several screening tools to collect demographic and social needs data have been piloted [11, 12], research has not assessed implementation in a broad range of primary care practices. Systematic and routine collection of demographic and social needs data is required to identify areas for intervention, inform strategies, and evaluate health equity interventions [1]. This study aims to assess the implementation of the SPARK tool, a demographic and social needs data collection tool, using eight implementation outcomes to capture practice change, primary care provider behavior, patient response, and inform systematic and routine data collection.

## Methods

This study assessed the implementation of the validated SPARK tool [13] in five primary care clinics in five of ten Canadian provinces: Saskatchewan, Manitoba, Ontario, Nova Scotia, and Newfoundland and Labrador. The SPARK tool includes 20 questions that assess demographics (language, immigration status, Indigenous identity, race, disability status, sex at birth, gender identity, sexual orientation, ethnicity, religion) and social needs (education, income, food security, medication access, housing status, transportation, phone and internet access, cost of utilities, social isolation, precarious employment) (Supplementary Appendix SA.1). We applied the Proctor et al. taxonomy for implementation outcomes [14]: acceptability, appropriateness, adoption, feasibility, fidelity, cost, penetration, and sustainability, to guide study conceptualization and design, supporting assessment and reproducibility of developed strategies (Supplementary Table S1) [14–16].

### Setting

This study recruited primary care clinics that varied according to location, size, scope of practice, and the type of Electronic Medical Record (EMR) used (i.e. PS Suite, MedAccess, Accuro). One clinic was located in ON in one of the largest cities in Canada, two were located in the prairies (SK, MB), and two were located in eastern Maritimes provinces (NS, NL). Clinics invited to participate included those operating in a community with a lower socio-economic status, with committed leadership and staff, demonstrated commitment to implement the SPARK tool, processes for community engagement (including Indigenous groups), continuous data quality assurance, and attending to discrimination complaints. Clinics ranged from 5 to 20 physicians and nurse practitioners providing care for 2000–14 000 patients. Participating clinics included a variety of other care providers including medical trainees (e.g. residents), social workers, mental health workers, and dietitians (Table 1).

**Table 1.** Description of clinics that implemented the SPARK tool in their clinic.

Clinic	A	B	C	D	E
Clinic staff participating	3/6	2/2	2/2	5/9	4/12
Family physician and nurse practitioners participating	4/6	6/6	1/9	4/22	4/20
Other providers at the clinic	Nurse, social worker, mental health worker, dietician, occupational therapy	Nurse, pharmacist, social worker	Nurse, mental health nurse, social worker, dietitian	Nurse, pharmacist, social worker, dietitian, residents	Nurse, residents
Patients seen 1 September 2022 to 31 August 2023	2850	1511	6613	12 719	7288
Estimated patient seen over 6-month study period	1425	756	3307	6360	3644
Patients seen per month (mean)	238	633	551	3910	607
SPARK surveys completed, *n* (% of patients seen in 6 months)	207 (14.5)	275 (36.4)	46 (1.4)	1249 (19.6)	77 (2.1)
Tablet, *n* (%)	18 (8.7)	90 (32.7)	16 (34.8)	15 (1.2)	2 (2.6)
Person device, *n* (%)	189 (91.3)	185 (67.3)	30 (65.2)	1234 (98.8)	75 (97.4)

### Data collection

The study team was comprised of provincial research coordinators to support implementation and assessment by reviewing expectations with local clinics, completing a clinic description and readiness checklist, and assessing clinic-specific implementation strategies. At a clinic-specific orientation, the study team discussed the SPARK tool, implementation, and study resources (Supplementary Appendix SA). A training module was used to review health equity, the study, and provide guidance on discussing the SPARK tool with patients (Supplementary Appendix SB). Training was evaluated using 13 questions to assess the module and readiness to implement (Supplementary Table S2). Clinic teams were comprised of participating primary care providers (e.g. family physicians, nurse practitioners, nurses, etc.), clinic administrative staff [e.g. reception, Information Technology (IT) staff], and clinic leadership (e.g. clinic managers, etc.) who were considered participants if they supported implementation and completed ≥1 survey.

Clinic-specific implementation spanned 6 months. Patients aged ≥18 years were offered the SPARK tool either prior to (appointment reminder, clinic tablet, or brochure with Quick Response (QR) code), during (administered by provider), or after (brochure with QR code) an appointment. Following completion of the SPARK tool, patients were invited to complete a seven-question feedback survey to assess ease-of-use, comfort, utility, and purpose. Dependent on the EMR and jurisdiction, the SPARK tool was integrated into the EMR using different strategies, including (i) OceanMD [17] or Pomelo [18] secure patient messaging, (ii) building the tool into an EMR form, and (iii) manual entry of the tool responses.

Clinic teams were provided with clinic-specific 3- and 6-month aggregate reports of SPARK tool responses. A 12-question mid-implementation survey assessed familiarity and utility of the SPARK tool. Throughout implementation, provincial coordinators maintained observational field notes of site visits, meetings, and email conversations documenting facilitators, challenges, and required adjustments.

### Analysis

Using descriptive statistics including frequency (%), and mean (SD), we characterized participating clinics, providers, and patients. We described the implementation outcomes [14–16] quantitatively using responses from the clinic description, training evaluation, mid-implementation survey, SPARK tool, and patient feedback survey. We contextualized quantitative results using observational field notes and open-ended responses.

Open-ended responses on the training, mid-implementation, and patient feedback surveys were thematically analyzed by provincial coordinators. Using a deductive approach, preliminary themes of expected responses were described. Following this, coordinators independently assessed and thematically grouped responses to each question. New themes were generated as coordinators reviewed the responses. Applying an iterative process, coordinators collectively discussed and adjusted themes to create final thematic groupings [19, 20]. There were three open-ended questions on the training module, “aspects most liked” and “areas to improve” produced four themes each, and “incorporating training into practice” produced two themes. On the mid-implementation survey, there were three open-ended questions on usefulness of the SPARK tool, challenges with implementation, and use of SPARK tool data. We identified eight themes around challenges and nine themes related to utility of the tool. The patient feedback survey had one open-ended question to obtain comments or concerns about the SPARK tool. The patient feedback survey produced eight themes: three improvement themes (tool improvement, gaps, contextual factors) and five implementation themes (overall thoughts, use of collected data, offering the tool, collecting social and demographic data in healthcare, and survey format). Results were summarized by each data source to describe the thematic groups, present the frequency of each group, and provide example quotes from respondents.

## Results

### Acceptability

Across all sites, 37.6% (*N* = 35/93) of eligible primary care providers and clinic staff (e.g. administrative staff, and clinic leadership) participated in this study, including 30.6% (*n* = 19/62) of providers and 51.6% (*n* = 16/31) of clinic staff (Table 1). Clinics described previous experience with similar data collection, largely during patient intake, and anticipated benefits from systematically using the SPARK tool. One provider shared, “*the theory behind the study, collecting data on socioeconomic factors to improve care and its delivery is not a new concept for me. However, having this information readily available in my patient's EMR will help me provide more informed and, therefore, appropriate care and treatment.*” Most participants completed the training evaluation (77.1%, *n* = 27/35), with 77.8% (*n* = 21/27) indicating training met expectations and prepared them for data collection. A minority of participants (14.8%) felt the training was too long and should incorporate implementation strategies or case examples (Table 2).

**Table 2.** Responses for participants that completed the SPARK training evaluation survey, *N* = 27.

How would you rate the training module	
Excellent, *n* (%)	11 (40.7)
Good, *n* (%)	10 (37.0)
Average, *n* (%)	6 (22.2)
Training had clear objectives (agree vs. neutral or disagree), *n* (%)	23 (85.2)
Training objectives were met (agree vs. neutral or disagree), *n* (%)	24 (88.9)
Training prepared me to collection SDH data in the clinic, *n* (%)	21 (77.8)
Training content was organized and easy to follow, *n* (%)	23 (85.2)
Time allotted was appropriate, *n* (%)	20 (74.1)
Training was interactive enough, *n* (%)	19 (70.4)
Easy to navigate module, *n* (%)	23 (85.2)
Training met expectations, *n* (%)	21 (77.8)
What did you like most about the training module	
Format and design, *n* (%)	12 (44.4)
Content, *n* (%)	8 (29.6)
Ease of access and easy to use, *n* (%)	8 (29.6)
Completing the SPARK tool myself, *n* (%)	4 (14.8)
How could the module be improved	
Long, *n* (%)	4 (14.8)
More case examples, *n* (%)	4 (14.8)
Will incorporate information from training in how discuss SPARK with patients, *n* (%)	9 (33.3)

### Adoption

**Figure 1:**
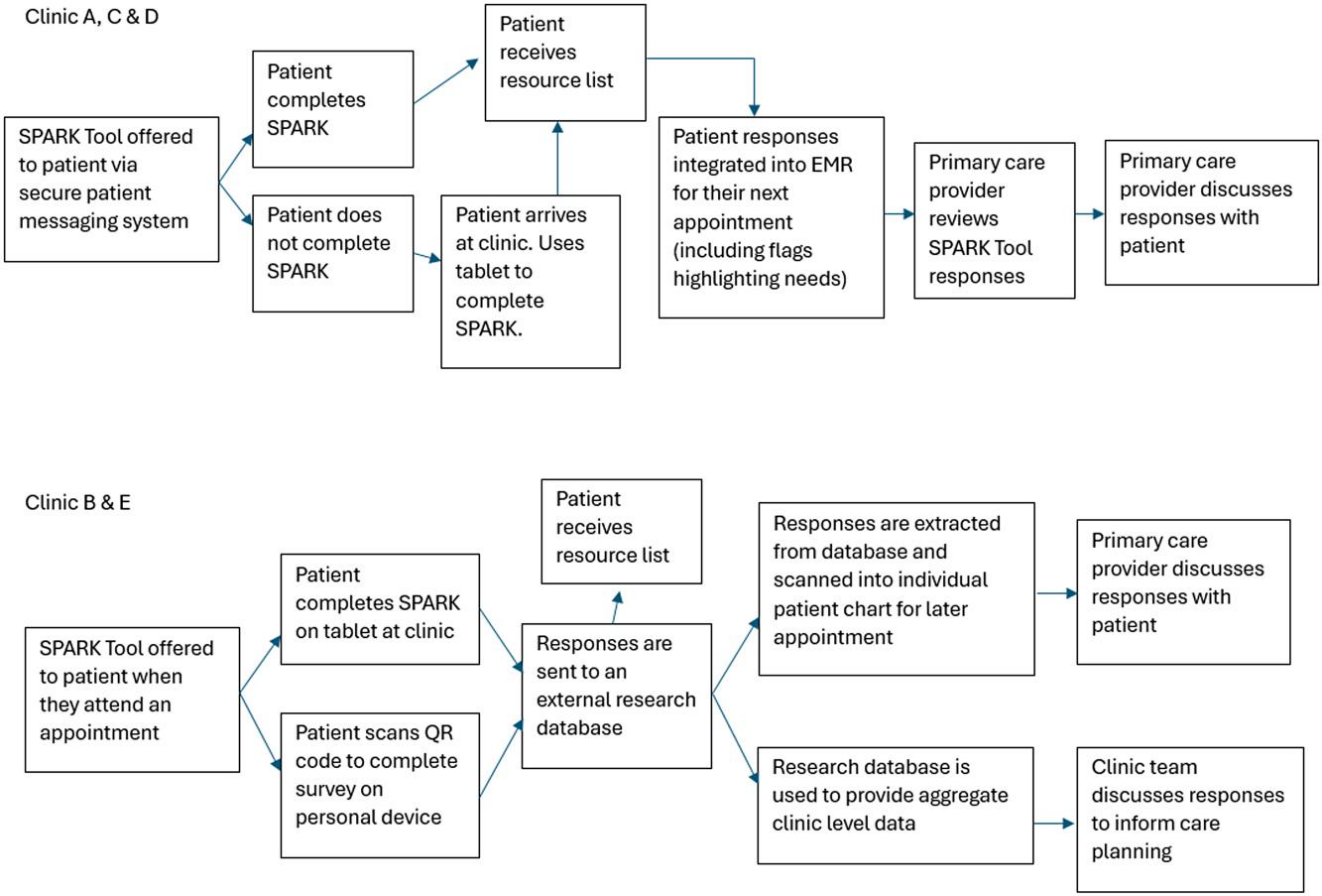
Clinic-specific SPARK tool implementation plans.

Clinic-specific implementation plans followed two workflows (Fig. 1). Clinics A, C, and D used an external service (i.e. OceanMD or Pomelo) to offer direct patient messaging and EMR integration. Among patients that did not receive a direct patient message (e.g. no email), clinic staff offered the tool on a tablet when they arrived at the clinic. Clinics B and E were unable to use an external integration service, therefore, the tool was offered through tablets and a QR code available at the clinic. Clinic teams were excited to receive SPARK tool responses and use data to inform improvements to patient care.

### Appropriateness

Patients who completed the survey using secure patient messaging took ∼15 (SD 11) min to complete the SPARK tool. The majority of patients indicated the tool was easy to complete (90.5%, *n* = 1238/1368) (Table 3). Although most patients (58.1%, *n* = 795/1368) indicated they were comfortable answering the tool questions, 10.2% (*n* = 139/1368) were extremely uncomfortable answering ≥1 question on the tool. One patient shared, “*I doubt this will make the slightest difference to improved treatment.*” Overall, patients indicated their experience completing the tool was excellent (51.0%, *n* = 697/1368) or good (33.6%, *n* = 459/1368), with 75.4% (*n* = 1031/1368) stating that they understood the purpose of the tool (Table 3).

**Table 3.** Patient responses to the SPARK tool feedback survey, *N* = 1368.

Variable	Clinic A *N* = 200	Clinic B *N* = 253	Clinic C *N* = 39	Clinic D *N* = 804	Clinic E *N* = 72	All clinics
SPARK was easy to complete (vs. difficult), *n* (%)	189 (94.5)	157 (62.1)	38 (97.4)	783 (97.4)	71 (98.6)	1238 (90.5)
Experienced difficulty answering a question (yes vs. no), *n* (%)	19 (9.5)	14 (5.5)	8 (20.5)	63 (7.8)	3 (4.2)	107 (7.8)
Comfort answering the questions (vs. discomfort), *n* (%)	116 (58.0)	91 (36.0)	23 (59.0)	517 (64.3)	48 (66.7)	795 (58.1)
Overall experience completing the survey						
Excellent, *n* (%)	83 (41.5)	98 (38.7)	21 (53.8)	455 (56.6)	40 (55.6)	697 (51.0)
Good, *n* (%)	86 (43.0)	55 (21.7)	15 (38.5)	276 (34.3)	27 (37.5)	459 (33.6)
Satisfactory, *n* (%)	21 (10.5)	8 (3.2)	2 (5.1)	49 (6.1)	4 (5.6)	84 (6.1)
Needs improvement, *n* (%)	3 (1.5)	1 (0.4)	1 (2.6)	17 (2.1)	1 (1.4)	23 (1.7)
Patient understood the purpose of SPARK						
Understood, *n* (%)	128 (64.0)	143 (57.5)	35 (89.7)	647 (80.5)	68 (94.4)	1031 (75.4)
Somewhat understood, *n* (%)	61 (30.5)	18 (7.1)	3 (7.7)	130 (16.2)	2 (2.8)	214 (15.6)
Did not understand, *n* (%)	5 (2.5)	3 (1.2)	0	23 (28.6)	1 (1.4)	32 (2.3)

Additional thoughts from patients (*n* = 237 all clinics represented)		
Theme	Subtheme	Example quote
Improvements (*n* = 107, 45.1%)	SPARK tool improvements (*n* = 85)	“Regarding the “Are you employed?” question, it might be useful to include “retired” among the possible answers.”
	Gaps in the SPARK tool (i.e. missing questions or subjects) (*n* = 15)	“There are social determinants of health and gaps in care that you are potentially missing… you don't ask about having children or caregiving for aging parents or other dependents. You're very focused on income and costs and food and housing insecurity… All parts of our identity and circumstances that define us and determine our health…”
	Capture of contextual factors (*n* = 31)	“I worry constantly that my housing will get beyond my ability to pay. While I would not classify myself as ‘poor’, my income is fixed, costs are out of control, and I sometime worry that I will have to choose between food and rent. I already minimize my food costs. Finances are a constant and debilitating worry, exacerbated by age.”
Implementation (*n* = 122, 51.5%)	Overall thoughts on SPARK tool and the study (*n* = 63)	“Very good little questionary, easy, but very good questions. I see the purpose of the questions.”
	Thoughts on implementation: providers using/acting on the information (*n* = 16)	“I doubt this will make the slightest difference to improved treatment”
	Thoughts on implementation: offering SPARK (*n* = 36)	“So many might not even talk about these things (income, social support) at a doctor's visit because of shame or fear of not being treated properly. But I appreciate this tool as it helps identify social determinants of health and looks at the person as a whole.”
	Thoughts on implementation: collection of data (*n* = 15)	“We are all human beings that come to the clinic for medical attention. Not sure what our Nationality had/have to do with seeking medical help. I was uncomfortable with that question.”
	Thoughts on implementation: format and survey administration (*n* = 20)	“I appreciated that you defined certain words that may not be in everybody's vocabulary and that you kept the reading level to a more basic level. If you were able to a) keep the sentences consistently short and b) create more white space in the survey you would increase reading accessibility. Thank you for your work!”

Perceived relevance and compatibility with clinic activities varied, dependent on methods for collection and integration. On the mid-implementation survey, most clinic staff and providers reported positive experiences with the tool (81.8%, *n* = 18/22), training (87.0%, *n* = 20/23), and implementation resources (90.9%, *n* = 20/22) (Table 4). One third (33.3%, *n* = 9/27) of respondents indicated that training prepared them to discuss the tool with patients (Table 2).

**Table 4.** Responses from clinic leads, clinic administrative staff and providers on the mid-implementation survey.

Question	All position types *N* = 30^a^
Experience using SPARK	
SPARK tool was extremely or very useful, *n* (%)	21/30 (70.0)
Positive experience with SPARK vs. fair or poor experience, *n* (%)	18/22 (81.8)
Positive experience with training and orientation vs. fair or poor experience, *n* (%)	20/23 (87.0)
Positive experience with pamphlets and materials vs. fair or poor experience, *n* (%)	20/22 (90.9)
Ease of Implementation (very easy or easy vs. neutral or difficult)	
Explain to patients, *n* (%)	13/21 (61.9)
Ask patients to complete, *n* (%)	5/19 (26.3)
Allocate resources, *n* (%)	4/18 (22.2)
Use survey interface, *n* (%)	8/16 (50.0)
Use tablet interface, *n* (%)	6/14 (42.9)
Manage tablets, *n* (%)	5/12 (41.7)
Find SPARK responses in EMR, *n* (%)	13/18 (72.2)
Read SPARK responses in EMR, *n* (%)	13/18 (72.2)
Challenges identified	
Length of SPARK in EMR, *n* (%)	6/18 (33.3)
SPARK formatting in EMR, *n* (%)	6/18 (33.3)
Discomfort discussing with patient, *n* (%)	5/18 (27.8)
How was the data used	
To understand patient circumstances, *n* (%)	13/19 (68.4)
To refer to services, *n* (%)	9/19 (47.4)
To change care, *n* (%)	7/19 (36.8)
Discuss patient concerns, *n* (%)	5/19 (26.3)

^a^Denominator changes because questions were not mandatory.

### Feasibility

Most patients completed the SPARK tool using their own device (92.4%, *n* = 1713/1854) instead of the clinic-based tablet (7.6%, *n* = 141/1854). Although 61.9% (*n* = 13/21) of providers and clinic staff indicated ability to explain the tool to patients, only 26.3% (*n* = 5/19) reported comfort during these discussions (Table 4). Providers and staff at clinics with EMR integration were more likely to report a positive experience (55.6%, *n* = 5/9) compared to those at nonintegrated clinics (42.9%, *n* = 3/7). However, across all clinics, only 22.2% (*n* = 4/18) of respondents indicated ease in resource allocation (Table 4). Compared to nonintegrated clinics, integrated clinics reported greater difficulty using and managing tablets (28.6%, *n* = 2/7 vs. 60.0%, *n* = 3/5). Clinics suggested paper surveys could support data collection when connectivity challenges were present. A third (33.3%, *n* = 6/18) of respondents had concerns with the length and format of the tool in the EMR. A provider shared, “*the [training] module should include information about where in the EMR the data from this tool can be accessed*.” Despite challenges, 72.2% reported positive experiences in finding and reading the SPARK tool responses in the EMR (Table 4).

### Fidelity

While our original study protocol required secure patient messaging and automated EMR integration, jurisdictional and clinic differences led to clinic-specific workflows (Fig. 1). Clinic brochures with QR codes and provider administered surveys were well received by clinics B and E. However, a provider mentioned, “*I'm not certain the staff were able to prioritize its distribution [which] may have contributed to poor response rates. EMR uploads (manual integration) are not data extraction friendly.*” Further to this we heard, “*while [SPARK is] useful, [there are] barriers to getting the [Tool] completed by all patients, particularly those who face more significant barriers to healthcare.*” Clinics suggested paper surveys could support collection from patient subgroups typically less represented (e.g. language barriers, less familiar with or limited access to technology). A respondent elaborated, “*Maybe helpful to have an annual or biannual follow-up survey tool that only includes questions that might change.*” Identifying Tool domains more likely to change (i.e. financial security) compared to domains less likely to change (i.e. race) can facilitate regular updates of SPARK tool responses.

### Cost

The readiness and feasibility checklist identified clinic requirements and implementation costs such as staff time, IT support, tablets, EMR integration software, and printing. Further, research coordinators supported implementation set-up and orientation, adjustments, and preparation of aggregate reports. Additional potential implementation costs discussed by clinics included hiring a peer or student to support tool completion, translators, and compensation.

### Penetration

Clinic B, which used manual integration, had the highest estimated completion rate (36.4%), followed by clinics using secure patient messaging (Clinic D, 19.6%; Clinic A, 14.5%) (Table 1). Availability of patient email addresses in the EMR varied. Clinic D had experience sending secure patient messages and a greater number of emails captured in their EMR compared to other participating sites. Contrary, Clinic C experienced the lowest patient response (1.4%) associated with limited email addresses in the EMR, requiring clinic staff to obtain patient consent prior to emailing the survey. Further, Clinic B was able to use both tablet and QR code to obtain patient responses, whereas Clinic E experienced internet connectivity issues most days, preventing the use of a tablet in their waiting room, which may have contributed to lower patient response (2.1%) (Table 1).

Among the 15 492 eligible patients, 1854 (12.0%) completed the SPARK tool (Table 1). With the exception of Clinic A, women were more likely to complete the tool. On average, 62.8% of respondents indicated ≥1 social need. Social needs identified varied by clinic; however, precarious employment and food insecurities were the most prominent (Supplementary Table S3). When discussing implementation one patient shared, “*I just hope that patients feel comfortable in sharing and are honest in providing accurate data about their personal situation so that changes can be made*” (Table 3). Patient comfort in responding to SPARK was a key theme discussed by patients, providers, and clinic staff.

### Sustainability

Clinic staff and providers indicated the SPARK tool was useful for understanding patient circumstances (68.4%, *n* = 13/19), referring patients to services (47.4%, *n* = 9/19), and informing care (36.8%, *n* = 7/19) (Table 4). Across all clinics, basic needs, housing stability, and food security were considered the most important, broader questions, to capture social needs data (Supplementary Table S4).

Among patients, 17.3% (*n* = 237/1368) provided thoughts on the tool; 45.1% (*n* = 107/237) suggested improvements to the tool, and 51.5% (*n* = 122/237) provided feedback on implementation (Table 3). Patients suggested improvements focused on SPARK tool questions (*n* = 85/107), gaps (*n* = 15/107), and contextual factors (*n* = 31/107) (Table 3). Further patients provided feedback on offering the tool (29.5%, *n* = 36/122), acceptability of data collection (12.3%, *n* = 15/122), and format, frequency, and mode of collection (16.4%, *n* = 20/122) (Table 3).

The aggregate clinic reports provided an opportunity to review and change services and assess sustainability of programs. Although there was positive feedback on the Tool, most participating clinics had no intention of continuing its use. Participating providers and clinic staff highlighted concerns with resource allocation and suggested the following adjustments: (i) hire personnel to support implementation; (ii) use well-developed EMR integration platforms; (iii) information to support practice change including support services; or (iv) offer the tool in smaller segments using a variety of modes.

## Discussion

The SPARK tool was implemented in five Canadian primary care clinics, screening 12.0% of eligible patients. Despite strong acceptability, there was variability in perceived appropriateness and feasibility related to implementation challenges, EMR integration, and contextual factors. Adjustments made during implementation supported data collection, integration, and use. Overall, primary care providers, clinic staff, and patients reported positive experiences with the tool. Providers and clinic staff indicated tool utility for understanding patient circumstances, referring patients to services, and informing care changes. Patients favorably reviewed the tool and were comfortable providing social and demographic information to their primary care provider. Despite this, half of the participating clinics did not have an intention of continuing its use. Clinics highlighted concerns with resource allocation and suggested adaptations to clinic workflows, enhanced integration platforms, and support for implementation that would contribute to sustainability of screening.

There is a growing body of research demonstrating benefit and willingness to collect and integrate social needs screening in primary care [1, 5, 9, 10, 13, 21–32]. Screening is feasible and useful for understanding patient context, starting conversations around social needs, and preventing inappropriate care [9, 22, 33, 34]. Studies have demonstrated value and feasibility in using a computer interface to screen patients [5]. Integration software and clinic-specific workflows affected the ease of implementation, response rates, and availability of the SPARK tool responses in the EMR. Similar to literature, training, time constraints, infrastructure, and availability of community resources were documented barriers affecting both implementation of SPARK and participation in this study [22, 32, 35]. Further, staff turnover during the study created fluctuations in participation by clinic staff. When implementing social needs screening, training should be available to new staff during onboarding. Less than a quarter of clinic staff (22.2%) indicated it was easy to allocate resources to data collection; however, automatic integration enhanced ease. Systematic collection is supported by context-informed flexible implementation strategies. Although most patients completed the tool on their personal device, having a variety of data collection modes ensured screening was possible when tablets were not charged, internet connection was unstable, and integration software was unavailable. The addition of different collection modes including paper surveys may have promoted increased patient response to the SPARK and supported access to screening by different population subgroups. While several screening tools have been implemented to collect demographic and social needs data [1, 5, 9, 13, 21–31], there has been little research assessing implementation in primary care practices. Integrating information on social challenges into the medical record ensures the entire care team considers social context during care planning [1, 5, 22, 36].

Screening not only supports individualized care but also enhances patient-provider relationships and facilitates system-level planning [1, 5, 9, 27]. Providers indicated screening was useful for understanding patient circumstances (68.4%), supporting referral (47.4%), and informing individualized care (36.8%). Tong et al. similarly noted that awareness of social needs improved knowledge of patients (53%) and influenced care delivery (23%) [27]. Facilitators to support social needs discussion include staff training, flexibility in data collection methods, dedicated staff providing adequate follow-up, resource lists, and rapport with providers [5, 32, 33, 36]. Implementing equity-focused quality improvement requires sustainable interventions supported by the health system [35, 36]. Traditionally, medicine focuses on risk factors with behavior modification as a main strategy for preventing disease; however, reducing unhealthy behaviors necessitates creating supportive environments [22]. Team-based care can facilitate social prescribing and system navigation [1, 2, 6, 22, 37]. Systematic screening can better allocate resources for care management and improve access to community resources [1, 2, 6, 34, 36]. Structural and policy changes are essential for the routine systematic collection of the social determinants of health, linking individual data to health records to enhance care and reduce health inequalities [5, 22, 34].

### Limitations

This study captures a diverse group of clinics, providers, and patients; however, the relatively small sample size impacts generalizability of its findings. Further, our study may have selection bias, participating clinics had an interest in collecting and using social data to support care provision. The small sample size and selection bias may produce differences in acceptability and feasibility of the tool, clinics and providers that participated may have differences not captured in this study. Further research with a larger sample should continue to assess implementation. During this study, the SPARK tool was only available in English. Availability of translation services to support survey completion depended on clinic resources. There were differences in availability of resources and services to assist patients with identified needs, which influenced clinic willingness to implement and sustain the SPARK tool. There were differences in how EMR vendors collected, stored, and displayed tool responses, impacting collection, access, and use of responses. Further to this, not all EMRs were able to distinguish if patients completed the SPARK tool, contributing to lower response rates in some clinics. The EMR and integration platform had implications for barriers, challenges, and opportunities experienced. Implementation must be tailored to the clinic context and may need to consider barriers or challenges not captured in this study.

## Conclusion

This study demonstrates acceptability and feasibility of implementing a brief screening tool to collect demographic and social needs in primary care. The SPARK tool was reviewed favorably by virtually all participants. However, there were practical insights with context-specific adjustments required to support collection, integration, and use. Collaboratively designed clinic workflows with seamless integration, multiple options for completion, and processes that minimize staff time are important for successful implementation. Routine systematic collection of demographic and social information in primary care settings can enable identification and measurement of health inequalities as well as support the development and evaluation of evidence-based health equity interventions.

## Supplementary Material

cmaf109_Supplementary_Data

## Data Availability

Aggregate data collected during this study have been provided within the article and as online supplemental information. Individual-level anonymous data are available from the corresponding author (A.D.P) upon reasonable request and with appropriate approvals. Data are not publicly available due to the confidential nature.
