# Supplementary material for: Implementing the screening for poverty and related social determinants and intervening to improve knowledge of and links to resources (SPARK) in primary care clinics across Canada
Source: Fam Pract. 2026 Apr 22;43(3):cmaf109. doi: 10.1093/fampra/cmaf109 (PMC13100817; doi:10.1093/fampra/cmaf109)
Supplement: cmaf109_Supplementary_Data [file cmaf109_supplementary_data.pdf]

Supplementary tables

Supplementary Table 1: Implementation Outcomes[13,14]	
**Outcome**	**Study Definition**
**Acceptability:** perception that a given intervention is agreeable, palatable, and satisfactory.	Perception of the SPARK. This domain assesses comfort with the intervention and perceived value of the tool.
**Adoption:** intention, initial decision, or action to try or employ an innovation or evidence-based practice.	Clinics intention to uptake or utilization the SPARK tool. This domain assesses the intention to adopt the intervention and initial uptake.
**Appropriateness:** perceived fit, relevance, or compatibility of the innovation or evidence-based practice for a given practice setting, provider, or consumer; and/or perceived fit of the innovation to address a particular issue or problem.	Perceived as relevant to primary care and compatible with other clinic activities. Although something may be seen as a good fit some features may not be appropriate for the context. This domain assesses the usefulness and relevance of the intervention in a specific context.
**Feasibility:** extent to which a new treatment, or innovation, can be successfully used or carried out within a given setting.	The suitability of the survey in a practical sense for the context and setting.
**Fidelity:** degree to which an intervention was implemented as it was prescribed	This domain assesses if the SPARK tool was delivered as intended including processes meant to prepare the team to deliver SPARK and assure adherence and quality while the intervention was being delivered.
**Cost:** cost impact of an implementation effort.	Specific costs of the intervention as well as missed opportunities (e.g. marginal cost, cost-effective or cost-benefit). Costs may include staff time for training and implementation, IT costs to support integration, and time taken away from other activities.
**Penetration:** integration of a practice within a service setting and its subsystems.	The degree and spread of integration including how many patients were invited to the survey, completion rate and response rate.
**Sustainability:** extent to which a newly Implemented treatment is maintained or institutionalized within a service setting's ongoing, stable operations.	The maintenance, continuation, durability, incorporation and sustained use of the SPARK tool.

Supplementary Table 2: Data collection Tools

Data collection tool	Who completed	Description
Pre-implementation		
Clinic description & readiness checklist	Provincial RC in conversation with clinic manager and clinic lead	The clinic description and readiness checklist included 21 questions aimed at understanding the current clinic context as well as past experiences with socio-demographic data collection at the clinic.
Training evaluation survey	Providers and clinic staff that completed the training	Brief 13 question survey to understand if the participants felt prepared to incorporate the SPARK tool into clinic processes.
Baseline focus group	Providers and clinic managers	Nine questions aimed at understanding (1) why the clinic chose to take part in sociodemographic data collection, (2) understanding proposed implementation and use, (3) anticipated concerns or challenges.
Implementation		
SPARK Tool	Patients	20 question (18 plus two optional questions) survey to capture a patient's demographic and social needs. There are eight demographic questions (language, immigration status, indigenous identity, race, disability, sex, gender identity, sexual orientation) and ten social needs questions (education, income, food security, medication access, housing stability, transportation, phone and internet access, utility bill affordability, social support, employment). Optional questions were ethnicity and religion.
SPARK feedback survey	Patients	Immediately following the SPARK tool patients were asked 7 questions focused on their experience completing the SPARK tool and comfort answering these questions for their provider.
Mid-implementation survey	Providers and clinic staff	13 survey questions to assess implementation as well as if the research team could further support implementation at the clinics.
Observational field notes	Providers, clinic staff	Excel spreadsheet to document and track correspondence with the clinic including success, challenges, and adjustments required.
Interviews	Clinic staff	12 questions aimed at understanding experience with administering the survey, impact on workflow, lessons learned, and ideas to improve.
Following implementation		
Focus group	Providers and clinic managers	Six questions to understand experience implementing SPARK, using the SPARK responses in practice and assess sustainability.

Supplementary Table 3: SPARK Aggregate Demographic Responses					
**Demographic Domain**	**A**	**B**	**C**	**D**	**E**
**Total responses**	**207**	**275**	**46**	**1249**	**77**
**Gender**					
Women	27.5%	72.4%	65.2%	64.6%	76%
Man	56.5%	26.2%	28.3%	32.3%	24%
Transgender man	3.9%	0.4%	0	0.1%	0
Transgender women	1.9%	0	0	0.1%	0
Gender fluid	4.8%	0	0	1.5%	0
Two spirit	3.4%	0	0	0	0
**Age**					
18-24 years	1.0%	10.2%	0	3.4%	10%
25-34 years	19.3%	18.2%	13.0%	12.7%	15%
35-44 years	16.9%	30.2%	13.0%	15.8%	25%
45-54 years	17.9%	18.5%	13.0%	13.1%	17%
55-64 years	31.4%	17.5%	23.9%	18.3%	13%
65+ years	13.5%	4.7%	45.7%	32.1%	17%
**Social needs**					
Patients with >=1 social need	76.3%	68.7%	67.4%	44.5%	57.1%
number of social needs identified, mean (SD)	2.21	1.88	1.7	0.79	1.3
Precarious employment	37.2%	41.1%	19.6%	25.6%	44.2%
Food insecurities	37.7%	34.5%	26.1%	10.8%	22.1%
Paying for basic needs	30.0%	25.1%	17.4%	7.2%	15.6%
Unable to pay for medication	22.2%	19.6%	17.4%	7.8%	16.9%
Missed utility payment	30.0%	26.5%	19.6%	4.6%	11.7%
Unable to pay for mortgage or rent	18.8%	13.8%	4.3%	5.0%	9.1%
Lack of transportation	17.4%	6.9%	17.4%	4.9%	6.5%
No social support	18.4%	10.5%	45.7%	7.7%	1.3%

No phone or internet	1.4%	0.7%	0	0.7%	0
Less than high school education	7.7%	9.1%	2.2%	5.1%	2.6%

**Supplementary Table 4: Mid-survey implementation most important SPARK questions**			
Most important demographic questions			
Factor	1st	2nd	3rd
Ethnicity, n(%)	**6/26 (23.1)**	1/26 (3.9)	2/26 (7.7)
Gender, n(%)	**4/26 (15.4)**	**9/26 (34.6)**	3/26 (11.5)
Indigenous identity, n(%)	**4/26 (15.4)**	3/26 (11.5)	3/26 (11.5)
Language, n(%)	**4/26 (15.4)**	0	**6/26 (23.1)**
Difficulty, n(%)	3/26 (11.5)	**7/26 (26.9)**	2/26 (7.7)
Race, n(%)	2/26 (7.7)	2/26 (7.7)	**4/26 (15.4)**
Newcomer, n(%)	2/26 (7.7)	**4/26 (15.4)**	2/26 (7.7)
Sexual orientation, n(%)	1/26 (3.9)	0	**4/26 (15.4)**
Religion, n(%)	0	0	0
Sex, n(%)	0	0	0
Most important social needs questions			
	1st	2nd	3rd
Basic needs, n(%)	**11/25 (44.0)**	3/26 (11.5)	**4/26 (15.4)**
Housing, n(%)	**6/25 (24.0)**	**6/26 (23.1)**	**8/26 (30.8)**
Food insecurity, n(%)	3/25 (12.0)	**6/26 (23.1)**	1/26 (3.9)
Education, n(%)	2/25 (8.0)	3/26 (11.5)	3/26 (11.5)
Ability to pay medications, n(%)	1/25 (4.0)	1/26 (3.9)	**4/26 (30.8)**
Social support, n(%)	1/25 (4.0)	2/26 (7.7)	**5 (19.2)**
Utilities, n(%)	1/25 (4.0)	3/26 (11.5)	0
Employment, n(%)	0	2/26 (7.7)	0
Phone and internet, n(%)	0	0	0
Transportation, n(%)	0	0	1/26 (3.9)

Appendix A: SPARK tool and Study Resources

A.1: SPARK Tool

SPARK Tool

Screening for Poverty And Related Social determinants to improve Knowledge of and links to resources (SPARK)

Demographics

1 Language

a) If available, would you prefer your healthcare appointments offered in another language?

b) If yes, which language?

2 Born in Canada

a) Were you born in Canada?

b) If no, when did you arrive?

3 Indigenous Identity

a) Do you identify as an Indigenous person?*

b) If yes, are you Status (Registered or Treaty Indian as defined by the Indian Act of Canada)?*

c) If yes, Inuk/Inuit, are you a member of an Inuit land claims agreement?*

**This data must be collected with engagement with local First Nations, Métis, and Inuit governance bodies in accordance with the First Nations OCAP, Métis OCAS, and Inuit Qaujimajatuqangit data governance and sovereignty principles.*

4 Race

In our society, people are often described by their race or racial background. Our race may influence the way we are treated by individuals and institutions, and this may affect our health. Which category(ies) best describes you? Select all that apply.

5 People with Disabilities

Do you currently experience any of the following due to a severe and persistent physical or mental condition? Select all that apply.

6 Sex at Birth

What was your sex at birth?

7 Gender Identity

What is your gender identity?

8 Sexual Orientation

Which category(ies) best describe your sexual orientation? Select all that apply.

? Descriptors

Patients can click on a hyperlinked "?" beside each question to learn about each question's purpose, a definition of terms, and why it is being asked.

Optional Questions

1 Ethnicity

What is your ethnic or cultural background? e.g., Chinese, Filipino, Guyanese, Scottish, Somali, Korean

2 Religion

What is your religious or spiritual affiliation? Select all that apply.

Social needs

9 Education

What is your current level of education?

10 Income/Finances

Do you currently have difficulty paying for basic needs?

11 Food Security

Please respond to the following statements:

a) "Within the past 12 months, we worried whether our food would run out before we could buy or get more."

b) "Within the past 12 months, the food we bought just didn't last and we could not buy or get more."

12 Medication Access

In the past 12 months, were you unable to get medicine or medical supplies, or did you do anything to make them last longer *because of the cost*?

13 Housing

a) What is your current housing situation?

b) Who do you live with? Select all that apply.

c) In the past 12 months, was there a time when you were not able to pay the mortgage or rent on time?

14 Transportation

In the past 12 months, has lack of transportation kept you from medical appointments, meetings, work, or from getting things needed for daily living? Select all that apply.

15 Phone and Internet Access

Do you currently have consistent access to a phone or the internet?

16 Utilities

In the past 12 months, did you miss making a payment on any utility bills (e.g., electric, gas/oil, water) *because of cost*?

17 Social Supports

a) Do you feel you have people who you can open up to or confide in?

b) Do you have people to rely on if you needed help?

18 Employment

a) Are you currently employed (this includes self-employed, full-time, part-time or other)?

If no:
b) Are you currently looking for work?

If yes:
c) Is your main job temporary or part-time (e.g., casual, contract, freelance, short-term, seasonal)?

d) Do you feel that your current employment could be negatively affected if you raised concerns about your work (e.g., health, safety, rights)?

e) In the past 12 months, did your income change a lot from month to month?

CIHR IRSC Canadian Institutes of Health Research / Instituts de recherche en santé du Canada

UPSTREAMLAB

Learn more at upstreamlab.org/spark

cc BY NC SA

A.2 Posters

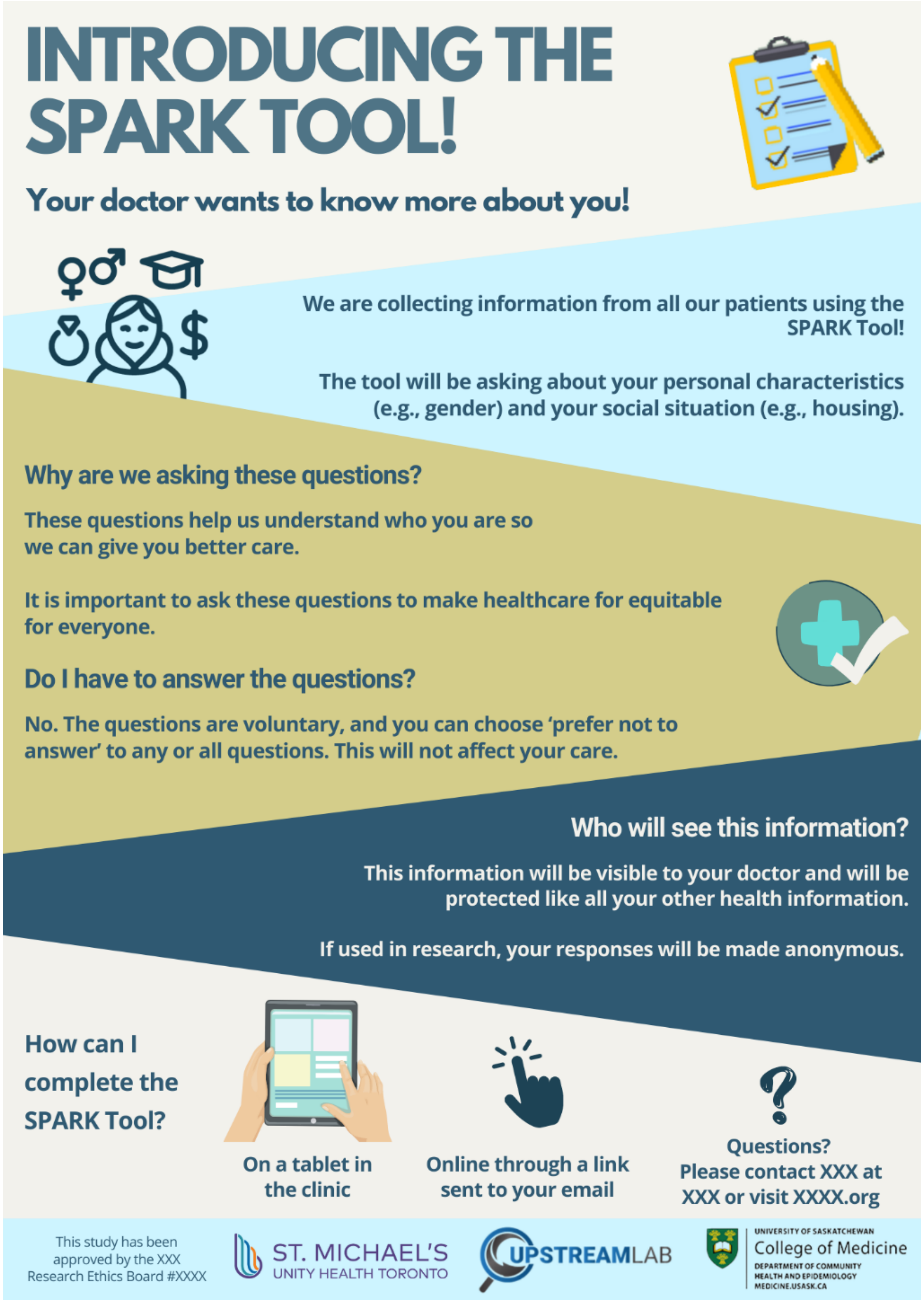
INTRODUCING THE SPARK TOOL!
Your doctor wants to know more about you!
We are collecting information from all our patients using the SPARK Tool!
The tool will be asking about your personal characteristics (e.g., gender) and your social situation (e.g., housing).
Why are we asking these questions?
These questions help us understand who you are so we can give you better care.
It is important to ask these questions to make healthcare for equitable for everyone.
Do I have to answer the questions?
No. The questions are voluntary, and you can choose 'prefer not to answer' to any or all questions. This will not affect your care.
Who will see this information?
This information will be visible to your doctor and will be protected like all your other health information.
If used in research, your responses will be made anonymous.
How can I complete the SPARK Tool?
On a tablet in the clinic
Online through a link sent to your email
Questions? Please contact XXX at XXX or visit XXXX.org
This study has been approved by the XXX Research Ethics Board #XXXX
ST. MICHAEL'S
UNITY HEALTH TORONTO
UPSTREAMLAB
UNIVERSITY OF SASKATCHEWAN
College of Medicine
DEPARTMENT OF COMMUNITY HEALTH AND EPIDEMIOLOGY
MEDICINE.USASK.CA

A.3 Brochures

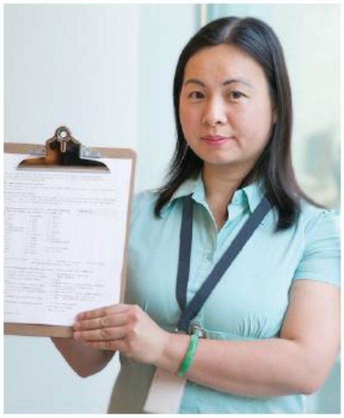

The SPARK Tool

Collecting Patients' Descriptive and Social Need Data in Health Care Settings

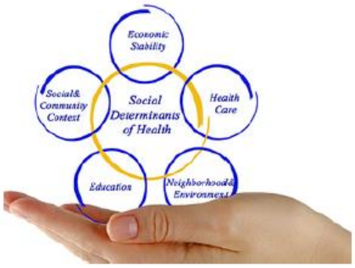

Your medical clinic is participating in a research study, which is testing the collection of descriptive and social needs data, or "sociodemographic information," in health settings.

The purpose of collecting this information is to understand **who our patients and clients are and what unique needs they may have**. The information you share with us will also improve our understanding of patient and client experiences and health outcomes.

"Sociodemographic" means information about a group of people that helps to describe them. Examples of the information we are collecting include:

- Language
- Racial/ethnic group
- Disability
- Gender Identity
- Sexual orientation
- Ability to pay for needs
- Social support

Why are you asking me these questions?

By asking these questions, we will understand our patients and become better at providing care and services. Also, sometimes people experience discrimination in healthcare. We want to ensure that this is not happening or address any form of discrimination happening at our clinic.

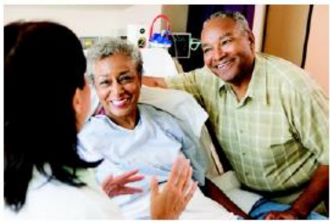

Is it legal to ask these questions?

Yes. Collecting data on human rights "grounds" (race, citizenship, sex, disability, etc.) for a human rights purpose is permitted and is in accordance with Canada's human rights legislative framework, including the Code, the Canadian Human Rights Act, the federal Employment Equity Act, and the Charter of Rights and Freedoms.

Not only is it legal to ask these questions, but the safe collection of this data is also urged by the Canadian Public Health Association.

We believe that we cannot fully understand patients' and clients' health care experiences without knowing more about who they are.

How will you use this information?

Your provider may review and use your information to improve your individual care. The clinic may use it to develop programs and do service training.

Members of your healthcare team may refer you to services, give you information, or identify unique needs, such as:

- Interpretation and translation services
- Health and care information
- Treatment programs
- Accommodation for disabilities
- Financial and similar supports related to housing, medication, utilities, food.

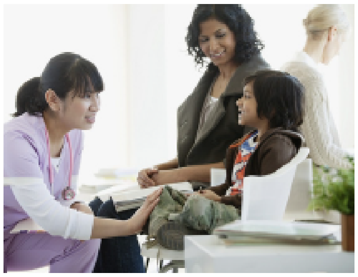

Who can see my demographic information?

We take your privacy very seriously.

(1) This information will be visible only to your health care team and protected like all your other health information.

(2) This information will remain confidential and will not be visible to anyone.

If used in research, the information from all patients and clients will be combined and researchers will not be able to identify who any of the patients and clients are.

I'm only here for a quick appointment. How is this relevant to my care?

It is important for us know who we serve, and whether patient and client needs match the care we provide.

This information will help us understand and plan care not only for your future visits but for other patients and clients who may have similar needs as yours.

What if there are questions that I don't want to answer?

You can select "prefer not to answer" to any or all questions. This will not impact the care you receive here.

Questions or Comments?

The clinic's front desk staff can answer many of the questions you may have about this survey known as the **SPARK Tool.**

If you have questions or concerns about the information you provide in the SPARK Tool or how your healthcare team may use it, you may discuss them with your healthcare provider at this clinic.

Members of the SPARK Research team would be happy to discuss the survey and this study with you.

If you have questions, please contact the SPARK team coordinator ....

This study is approved by the Behavioural Research Ethics Board. Project ID:

A.4 SPARK tool workflow

SPARK Tool
Workflow for Clerical Staff

1

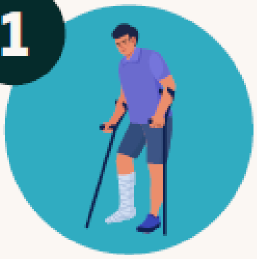

Patient enters clinic:

1. **Register patient following standard clinic procedure**
2. **Ask**: "Have you completed the SPARK Tool survey?"

If patient has already completed survey, they are good to go!

2

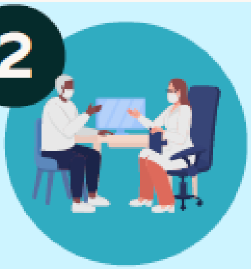

Provide a brief description:

"The SPARK Tool is a survey to collect descriptive and social needs data about patients to help our clinic provide better and more equitable care to our patients. Are you interested?"

- 18 (plus 2 optional) questions and takes about 15 minutes.
- Use supporting documents to answer questions.

3

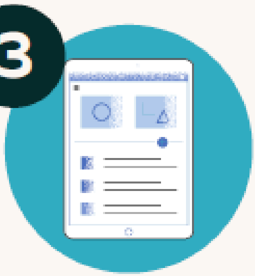

Three ways patients can do the survey:

1. **Ask**: "Please enter your health card number or provincial health number and complete on the tablet." or
2. **Ask**: "Please see us before leaving to complete on the tablet." or
3. **Ask**: "Please click on the link in your appointment reminder".

4

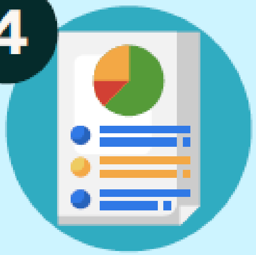

Patient responses are used in four ways:

1. Saved to secure Electronic Medical Record (EMR).
2. Saved to EMR **and** research study (if patients consent to have their de-identified responses included in research).
3. EMR data is visible to healthcare providers.
4. EMR data may also be used for quality improvement at the clinic.

St. Michael's Research Ethics Board #21-306. Version current as of 10/6/2022, #1.

A.5 Key messages about the SPARK tool

Three Key Messages

For Introducing the SPARK Tool to Patients

Why

- Find out who we serve
- Identify patient needs
- Provide better care to patients

Voluntary

- The questions are voluntary
- You can choose 'prefer not to answer' to any/all questions
- Your responses will not affect your care

Confidential

- Responses only visible to healthcare team
- Protected like all health information
- If used in research, patients' responses will be grouped together
- Researchers will not be able to identify you

A.6 Sample Script for Staff

SAMPLE SCRIPTS FOR HANDLING PATIENT QUESTIONS ABOUT THE SPARK TOOL

**Patient Question**	**What does this have to do with my care?**
**Script**	"We want to provide care based on our patients' needs. We don't want to make any assumptions about patient needs or who our patients are.<br><br>"Having this information gives us an idea of who visits our clinic."<br><br>"In some cases, depending on the patient and the situation, knowing this information means providing better care. For example, when we know a patient doesn't speak English, we will work towards obtaining interpreter services."
**Patient Question**	**"I'm just here for a quick appointment."**
**Script**	"This information is for both now and future visits as well".
**Patient Question**	**This has nothing to do with me - so what if I say (e.g. straight)?**
**Script**	"We ask everyone these questions. Depending on the patient's response, we may be able to refer them to services or talk to them about any needs they have."
**Patient Question**	**Do I have to answer these questions?**
**Script**	"No, it's completely voluntary. You can choose to not complete the survey or select 'Prefer not to answer' for any questions you do not want to answer."
**Patient Question**	**Who will see this information?**
**Script**	"Your provider(s) will see this information, and it will become part of your medical record. Your information is confidential and protected by law, just like all of your other health information."
**Question**	**How will this information be used?**
**Script**	"Your provider(s) will use this information to help meet your healthcare needs. In addition, gathering this information from all patients allows the clinic to see if there are gaps in care or services across different populations. Learning this tells us if we need to improve the care we give to our patients."<br><br>"With your permission, which you will be asked to provide after completing the SPARK Tool, a research team may access your anonymous data which will be grouped with other patients' data to study the use and value of this survey in our clinic. The researchers will not be able to identify you. "

Local Resources in [City]

XXX 211: for help finding programs and services call 2-1-1 or visit http://

INCOME SUPPORT:

List of resources including name, services provided, contact information and hours

AGENCIES OFFERING MEALS:

List of resources including name, services provided, contact information, and hours

TRANSPORTATION SERVICES

List of resources including name, contact information and hours

Local Resources in [City]

HOUSING SUPPORT:

UTILITIES SUPPORT

EMPLOYMENT SUPPORT

Local Resources in [City]

SOCIAL SUPPORT

NEWCOMER SUPPORT

INDIGENOUS SUPPORT

2SLBGTQ+ SUPPORT

ACKNOWLEDGEMENT

This resource list was created for the SPARK study, a CIHR-funded national study aimed at developing a survey for sociodemographic data collection in healthcare. This project was approved by the...

For additional information on resources available in your area please visit

Definitions of Key Terms in SPARK Tool

Socio-demographics

Indigenous Identity

Truth and Reconciliation Commission of Canada gives calls to action.[5] These calls to action can improve outcomes for First Nations, Métis, and Inuit peoples in Canada.[5]

Data sovereignty means that each nation governs the collection, ownership, and application of its data. No matter where that data is stored[1-4].

Indigenous: In Canada, Indigenous peoples are First Nations, Métis, and Inuit. These are the original people of the land that is now Canada.[6-9]

First Nations are the original people of the land that is now Canada. First Nations may or may not be Status (registered under the Indian Act) or live in a First Nations community.[10]

Métis have their own history, culture, language, and territory. Métis are people born in the Métis Nation Homeland (Manitoba, Saskatchewan, Alberta, as well as parts of Ontario, British Columbia, and the Northwest Territories).[11]

Inuit are the original people of the Northern region of Canada, Alaska, and Greenland. Their homeland included much of the land, water, and ice in the Artic.[7,12]

Status (Registered or Treaty Indian) is a person registered under the Indian Act of Canada. People with status may receive health services under the Non-Insured Health Benefits Program.[13,14]

A member of an **Inuit land claims agreement** is a beneficiary of: Nunavut Land Claim Agreement, or Inuvialuit Final Agreement.[15]

Race and Ethnicity

Race is a term used to classify people into groups usually based on observable physical characteristics (for example, skin colour) but can also include characteristics such as accent or dress.[1,2,3]

Ethnicity refers to groups of people who share a common culture or ancestry. They may also share a common language, religion, geographic origin, nationality, cultural traditions, migration history, or other commonalites.[1,2]

Religious Affiliation

"Religious or spiritual affiliation" is about your connection with a particular set of beliefs, attitudes, or practices. This may or may not be related to a specific religion (for example, Catholicism, Islam) or belief in a higher being.[1] This also relates to a person's sense of spirituality.

Sex at Birth

Sex at Birth is often recorded at a person's birth and is usually based on a person's reproductive system and other physical traits.[1]

Intersex refers to people born with differences in their reproductive features[2,4] that may not be easily characterized as male or female.[5]

Gender Identity

Gender Identity is a person's sense of themselves relating to gender, be it male, female, a combination of both, or neither.[1] Gender can be fluid and may change over time.[2]

Gender non-binary describes a person who may experience a gender identity that is neither exclusively woman or man, is a combination of woman and man or is between or beyond genders.[4]
Non-binary can also be used as an umbrella term for identities that are gender fluid, moving between and through identities.[1]

Two-Spirit is a term used by Indigenous peoples to self-describe and express diverse sexual, gender and/or spiritual identities.[5] It is a way for Indigenous peoples to reconnect with their traditional languages, ways, and cultures.[6]

Transgender or trans is an umbrella term for people whose gender identity or expression is different from conventional or cultural expectations based on the sex they were assigned at birth.[7,8]

Sexual Orientation

Sexual orientation is a term for the emotional, physical, romantic, sexual, and spiritual attraction, non-attraction, desire, or affection for another person.[1]

Aromantic describes a romantic identity for people who do not experience romantic attraction to people of any gender but may still have sexual attraction to other people.[2]

Asexual describes people who do not experience sexual attraction to people of any gender but may still experience romantic attractions to other people.[1,3]

Bisexual describes a person whose primary emotional, romantic, or sexual attraction is toward people of both the same and different gender.

Demisexual describes a person who feels sexual attraction only to people that they have an emotional bond with.[3]

Homosexual or Gay describes a person whose primary emotional, romantic, or sexual attraction is toward people of the same gender.[1]

Heterosexual or Straight describes a person whose primary emotional, romantic, or sexual attraction is toward people of an opposite gender.

Lesbian describes a person, usually a woman, whose primary emotional, romantic, or sexual attraction is toward people of the same gender.[1]

Pansexual describes a person who has emotional, romantic, or sexual desire for people of all genders and sexes.[1]

Queer is a term used to express a range of identities and orientations that are beyond the mainstream.[1]

Two-Spirit is a term used by Indigenous peoples to self-describe and express diverse sexual identities as having both a masculine and feminine spirit.[4] It is a way for Indigenous peoples to reconnect with their traditional languages, ways, and cultures.[5]

Social Needs

Education

Some high school means high school education may be in progress or stopped.

Ongoing means schooling or training is in progress.

Basic Needs

Basic Needs are things that a person requires to achieve and maintain physical and mental well-being and include food, water, shelter, and clothing.[1-3]

Medication and Medical Supplies

Medicine includes both prescription and over-the-counter medicines.

Medical supplies are any equipment needed to manage health or disability. For example, needles, glucose monitors, walking aids.

Housing Situation

Social housing is housing that is subsidized or partly paid for by a level of government.[1]

Subsidized housing means receiving help paying for housing from the government or a private organization.[2]

Rent-geared-to-income is a type of rent assistance. It lets a person pay a percentage of their income for rent.[3]

Supportive housing refers to housing with on-site staff who provide residents with ongoing support with their needs.

Transportation

"Medical Appointments" are appointments to meet your health needs. This may include an appointment with your doctor(s), specialist, or social worker. This also includes getting medicine from your doctor or pharmacist.[1]

Lack of transportation may be because of:[2]

- cost and access,
- distance,
- gas or parking costs,
- public transportation safety,
- and/or other reasons.

“Things needed for daily living” are things such as groceries or personal products that you need to be healthy.

Phone and Internet

“Consistent access” means having regular, reliable, and adequate access to a phone or internet to obtain needed services. This includes access to a phone or internet for a healthcare appointment.[1] This also includes access to health information that is on the internet that your provider refers you to.[1]

Basic Utilities

Basic utilities[1] may include:

- heating or cooling
- electricity or hydro
- water or sewer
- phone, and internet.

Basic utilities may vary by where a person lives. For example, homes may be heated using natural gas, furnace oil or wood depending on where the home is.

Social Supports

Having **“people you feel you can open up to or confide in”** means being able to talk to people that you care about, trust and feel close to.[1]

Employment

Health and safety concerns at work may include concerns about unsafe work (for example, lifting items that are too heavy, working with violent clients), unsafe work environments (for example, slippery floors, broken machinery), or harassment due to race, gender, or sexual orientation.[1]

Concerns about **employment rights** may include questions about the right to breaks, sick leave or vacation time.[1]

Health and safety rights[1] include the right to:

- know about unsafe work
- refuse unsafe work
- participate in workplace health and safety decisions

References

Indigenous identity

1. Canadian Institute for Health Information. (2020). A path forward: toward respectful governance of First Nations, Inuit and Metis Data Housed at CIHI. Accessed April 14, 2022.
2. First Nations Information Governance Centre. The First Nations principles of OCAP®. Accessed April 11, 2022.
3. University of Manitoba Faculty of Health Sciences. (2019). Framework for Research Engagement with First Nation, Metis, and Inuit Peoples. Accessed April 11, 2022.
4. Tagalik S. (2009–2010). National Collaborating Centre for Aboriginal Health. Inuit Qaujimajatuqangit: The Role of Indigenous Knowledge in Supporting Wellness in Inuit Communities in Nunavut. Accessed April 11, 2022.
5. Truth and Reconciliation Commission of Canada. (2015) Truth and Reconciliation Commission of Canada: Calls to Action. Accessed April 11, 2022.
6. Statistics Canada. (2021). Indigenous group of person. Accessed April 11, 2022
7. The Canadian Encyclopedia. Indigenous Peoples in Canada Accessed April 11, 2022
8. United Nations. Indigenous Peoples at the United Nations Accessed April 11, 2022
9. United Nations. United Nations Declaration on the Rights of Indigenous Peoples. 2018
10. Government of Canada. (2014). First Nations People in Canada. Accessed April 11, 2022
11. Libraries and Archives Canada. Government of Canada. (2021) First Nations People in Canada. Accessed April 11, 2022
12. Government of Canada. (2021). Inuit. Accessed April 11, 2022
13. Government of Canada. (2022). About Indian Status. Accessed April 14, 2022
14. Statistics Canada. (2021). Registered or Treaty Indian status of person. Accessed April 11, 2022
15. Government of Canada. Your Health Benefits – A Guide for Inuit to Access Non-Insured Health Benefits. Accessed April 22, 2022.

Race and Ethnicity

1. Canadian Institute for Health Information (2022). Guidance on the Use of Standards for Race-Based and Indigenous Identity Data Collection and Health Reporting in Canada Ottawa, ON: CIHI. Accessed April 5, 2022
2. Torontohealthequity.ca. Measuring Health Equity: Demographic Data Collection in Health Care. Accessed April 5, 2022
3. Government of Ontario Anti-Racism Directorate. Data Standards for the Identification and Monitoring of Systemic Racism. Accessed April 5, 2022

Sex at Birth

1. Statistics Canada, Government of Canada (2021). Sex at birth of person Accessed April 18, 2022
2. Human Rights Campaign (2011). Glossary of Terms - Human Rights Campaign. Accessed April 18, 2022
3. Torontohealthequity.ca. Measuring Health Equity: Demographic Data Collection in Health Care. Accessed April 18, 2022
4. Ucdavis.edu. (2014). LGBTQIA Resource Center Glossary | LGBTQIA Resource Center. Accessed April 18, 2022
5. Ohrc.on.ca. (2014). Appendix B: Glossary for understanding gender identity and expression | Ontario Human Rights Commission (ohrc.on.ca). Accessed April 18, 2022

Gender Identity

1. Ohrc.on.ca. (2014). Appendix B: Glossary for understanding gender identity and expression | Ontario Human Rights Commission. Accessed April 18, 2022
2. Torontohealthequity.ca. Measuring Health Equity: Demographic Data Collection in Health Care. Accessed April 18, 2022
3. Statistics Canada, Government of Canada (2021).Sex at birth of person. Accessed April 18, 2022
4. Losty, M. and O'Connor, J. (2017). Falling outside of the 'nice little binary box': a psychoanalytic exploration of the non-binary gender identity. Psychoanalytic Psychotherapy, 32(1), pp.40–60
5. Knudson, S. and Hahn, D. (2019). Committing sociology: critical perspectives on our social world. Toronto: Pearson Canada.
6. Fewster, P.H. (2018). Researching for LGBTQ Health. Lgbtqhealth.ca. Accessed April 18, 2022
7. Ucdavis.edu. (2014). LGBTQIA Resource Center Glossary | LGBTQIA Resource Center. Accessed April 18, 2022
8. Human Rights Campaign (2011). Glossary of Terms - Human Rights Campaign. Accessed April 18, 2022

Sexual Orientation

1. Human Rights Campaign (2011). Glossary of Terms - Human Rights Campaign. Accessed April 18, 2022
2. Suen, L.W., Lunn, M.R., Katuzny, K., … & Obedin-Maliver, J. (2020). What Sexual and Gender Minority People Want Researchers to Know About Sexual Orientation and Gender Identity Questions: A Qualitative Study. *Archives of Sexual Behavior,* 49(7), pp.2301–2318.
3. Ucdavis.edu. (2014). LGBTQIA Resource Center Glossary | LGBTQIA Resource Center. Accessed April 18, 2022
4. Knudson, S. and Hahn, D. (2019). *Committing sociology: critical perspectives on our social world.* Toronto: Pearson Canada.
5. Fewster, P.H. (2018). Researching for LGBTQ Health. Lgbtqhealth.ca. Accessed April 18, 2022

Basic Needs

1. Collin, C. and Campbell, B. (2008). Measuring Poverty: A Challenge for Canada Accessed April 14, 2022
2. Sarlo C. (2013). Poverty: Where do we draw the line? Accessed April 13, 2022
3. Homeless Hub. (2021). Basic Needs. Accessed April 13, 2022

Housing Situation

1. www.homelesshub.ca. Affordable Housing | The Homeless Hub. Accessed April 7, 2022
2. Settlement.org. (2018). What is subsidized housing? Accessed April 7, 2022
3. www.durham.ca. (2021). Rent-Geared-to-Income Housing (RGI) - Region of Durham . Accessed April 7, 2022

Transportation

1. National Association of Community Health Centre, Inc, Association of Asian Pacific Community Health Organizations, and the Oregon primary Care Association. (2019). PRAPARE. Protocol for Responding to and Assessing Patients' Assets, Risks and Experiences. Implementation and Action Toolkit. Accessed April 14, 2022
2. Syed, ST., Gerber, BS., Shapr, LK. (2013). Traveling toward disease" transportation barriers to health care access. J community Health, 38(5), pp. 976-993.

Phone and Internet

1. Graves, JM., Abshire, DA., Amiri, S., Mackelprang, JL. (2021). Disparities in technology and broadband internet access across rurality: implications for health and education. Fam Community Health, 44(4), pp. 257-265.

Basic Utilities

1. International Policy Centre for Inclusive Growth. (2009). Equitable access to basic utilities: Public versus private provision and beyond. Accessed: April 20, 2022.

Social Supports

1. National Association of Community Health Centre, Inc, Association of Asian Pacific Community Health Organizations, and the Oregon primary Care Association. (2019). PRAPARE. Protocol for Responding to and Assessing Patients' Assets, Risks and Experiences. Implementation and Action Toolkit. Accessed April 14, 2022

Employment

1. Lewchuk, W. (2013). The limits of voice: Are workers afraid to express their health and safety rights. Osgoode Hall LJ, 50(4), p. 789-812. Accessed April 20, 2022

Religious Affiliation

1. Chatters, L. (2000). 'Religion and Health: Public health research and practice,' Annual Review of Public Health, 21, pp. 335-367.

UPSTREAMLAB

Updated SPARK Survey – Final Version

June 22, 2022

The descriptor is a hyperlinked ? beside the question that provides clarifications, definitions of terms and explains why are asking the question. Clients can hover over or click on the hyperlinked ? to show a pop-up window (depending on survey platform)

Domain	*Question (if branching or skip logic* is not used)*<br><br>***Skip logic is highly recommended**	*If Branching or Skip Logic is used*	*Descriptor**<br><br>***If possible, we recommend adding hyperlinked ? to the front of the question or to the front the option (for definitions), where applicable.**<br><br>For example:<br>**What was your sex at birth?**<br>• Female<br>• Male<br>• Intersex ?<br>• Do not know<br>• Prefer not to answer<br><br>Include descriptor with *Why are we asking this question?* And *Understanding the question* with definition of sex and birth and gender identity<br><br>Include definition of intersex

Demographics			
**For Research Only – To be removed from surveys after 6-month research period**<br><br>**Age**	**What age group do you belong to?**<br>• Under 18<br>• 18-24<br>• 25-34<br>• 35-44<br>• 45-54<br>• 55-64<br>• 65 or over	Not Applicable	Not Applicable
**1: Language**	**a) If available, would you prefer your healthcare appointments offered in another language?**<br>• Yes<br>• No<br>• Do not know<br>• Prefer not to answer<br><br>b) **If yes, which language?** (drop-down menu)<br>• English [if applicable]<br>• French [if applicable]<br>• Arabic<br>• Cantonese<br>• Dene<br>• German<br>• Greek<br>• Gujarati<br>• Hindi<br>• Inuktitut	**a) If available, would you prefer your healthcare appointments offered in another language?**<br>• Yes → Go to question 1b<br>• No → Go to question 2<br>• Do not know → Go to question 2<br>• Prefer not to answer → Go to question 2<br><br>b) [If yes in 1a] **Which language?** (drop-down menu)<br>• English [if applicable]<br>• French [if applicable]<br>• Arabic<br>• Cantonese<br>• Dene<br>• German<br>• Greek<br>• Gujarati<br>• Hindi<br>• Inuktitut	**Why are we asking this question?**<br><br>This information lets us know who may want an interpreter or translation services at our clinic.<br><br>This may also help us provide appropriate care to people who do not speak or read English or French.

	• Italian<br>• Korean<br>• Mandarin<br>• Montagnais (Innu)<br>• Ojib-Cree<br>• Ojibway<br>• Persian (Farsi)<br>• Polish<br>• Portuguese<br>• Punjabi (Panjabi)<br>• Romanian<br>• Russian<br>• Spanish<br>• Tagalog (Pilipino, Filipino)<br>• Tamil<br>• Urdu<br>• Vietnamese<br>• Other (specify)___________<br>• Not applicable, I would not prefer healthcare appointments offered in another language [remove if skip logic used]	• Italian<br>• Korean<br>• Mandarin<br>• Montagnais (Innu)<br>• Ojib-Cree<br>• Ojibway<br>• Persian (Farsi)<br>• Polish<br>• Portuguese<br>• Punjabi (Panjabi)<br>• Romanian<br>• Russian<br>• Spanish<br>• Tagalog (Pilipino, Filipino)<br>• Tamil<br>• Urdu<br>• Vietnamese<br>• Other (specify)___________<br><br>Note: This represents the top non-Indigenous *languages most spoken at home* in Canada and top 5 Indigenous languages spoken in Canada according to the 2016 Census<br><br>This list should be modified based on region. For example, using the top 20 *languages most spoken at home* in Ontario for SPARK Survey use in Ontario etc.	
**2: Born in Canada**	**a) Were you born in Canada?**<br>• Yes<br>• No<br>• Do not know<br>• Prefer not to answer	**a) Were you born in Canada?**<br>• Yes → Go to question 2b below<br>• No → Go to question 3<br>• Do not know → Go to question 3<br>• Prefer not to answer → Go to question 3	**Why are we asking this question?**<br>Providers may be able to refer people to appropriate newcomer services. This information lets us know who we serve at the clinic and may help us identify newcomers to Canada.

<table>
<tr>
<td></td>
<td>b) If no, when did you arrive?
less than 5 years ago
5 to 9 years ago
10 years ago or more</td>
<td>b) [If yes in 2a] When did you arrive?
less than 5 years ago
5 to 9 years ago
10 years ago or more</td>
<td></td>
</tr>
<tr>
<td>3: Indigenous Identity</td>
<td>a) Do you identify as an Indigenous person? Select all that apply:
Yes, First Nations
Yes, Métis
Yes, Inuk/Inuit
Yes, another Indigenous identity (specify) ____________
No
Do not know
Prefer not to answer</td>
<td>a) Do you identify as an Indigenous person? Select all that apply:
Yes, First Nations → Go to question 3b
Yes, Métis → Go to question 3b
Yes, Inuk/Inuit → Go to question 3b and 3c
Yes, another Indigenous identity (specify) ____________ → Go to question 3b
No → Go to question 4
Do not know → Go to question 4
Prefer not to answer → Go to question 4</td>
<td>Why are we asking this question?

Your provider may use this to refer you to First Nations, Métis or Inuit resources and supports. This information lets us know who we serve and helps us meet patient needs.

First Nations, Métis or Inuit identity data must be collected with local First Nations, Métis, and Inuit governance bodies. This way, the information is appropriately collected, stored, interpreted, used, or shared.[1-4] This is in line with the First Nations OCAP, Métis OCAS, and Inuit Qaujimajatuqangit data governance and sovereignty principles.[1-4]

This information helps identify and address differences in health outcomes between First Nations, Métis and Inuit people and other racial groups in Canada.[5]

Understanding the question:

We ask "Do you identify…" in the question because we are asking you to tell us if you feel you are part of this group. This may include people who are Status or not Status.

Truth and Reconciliation Commission of Canada gives calls to action.[5] These calls to action can improve outcomes for First Nations, Métis, and Inuit peoples in Canada.[5]</td>
</tr>
</table>

			**Data sovereignty** means that each nation governs the collection, ownership, and application of its data. No matter where that data is stored[1-4].<br><br>**Indigenous:** In Canada, Indigenous peoples are First Nations, Métis, and Inuit. These are the original people of the land that is now Canada.[6-9]<br><br>**First Nations** are the original people of the land that is now Canada. First Nations may or may not be Status (registered under the Indian Act) or live in a First Nations community.[10]<br><br>**Métis** have their own history, culture, language, and territory. Métis are people born in the Métis Nation Homeland (Manitoba, Saskatchewan, Alberta, as well as parts of Ontario, British Columbia, and the Northwest Territories).[11]<br><br>**Inuit** are the original people of the Northern region of Canada, Alaska, and Greenland. Their homeland included much of the land, water, and ice in the Artic.[7,12]<br><br>1. Canadian Institute for Health Information. (2020). A path forward: toward respectful governance of First Nations, Inuit and Metis Data Housed at CIHI. Accessed April 14, 2022.<br>2. First Nations Information Governance Centre. The First Nations principles of OCAP®. Accessed April 11, 2022.<br>3. University of Manitoba Faculty of Health Sciences. (2019) Framework for Research Engagement with First Nation, Metis, and Inuit Peoples. Accessed April 11, 2022.<br>4. Tagalik S. (2009–2010) National Collaborating Centre for Aboriginal Health. Inuit Qaujimajatuqangit: The Role of Indigenous Knowledge in Supporting Wellness in Inuit Communities in Nunavut. Accessed April 11, 2022.<br>5. Truth and Reconciliation Commission of Canada. (2015) Truth and Reconciliation Commission of Canada: Calls to Action. Accessed April 11, 2022.<br>6. Statistics Canada. (2021). Indigenous group of person. Accessed April 11, 2022<br>7. The Canadian Encyclopedia. Indigenous Peoples in Canada Accessed April 11, 2022

			8. United Nations. Indigenous Peoples at the United Nations Accessed April 11, 2022<br>9. United Nations. United Nations Declaration on the Rights of Indigenous Peoples. 2018<br>10. Government of Canada. (2014) First Nations People in Canada. Accessed April 11, 2022<br>11. Libraries and Archives Canada. Government of Canada. (2021) First Nations People in Canada. Accessed April 11, 2022<br>12. Government of Canada. (2021) Inuit. Accessed April 11, 2022
	b) **If yes, are you Status (Registered or Treaty Indian as defined by the *Indian Act* of Canada)?**<br>• Yes, Status Indian (Registered or Treaty)<br>• No<br>• Not applicable, I am not an Indigenous person [remove if skip logic used]<br>• Do not know<br>• Prefer not to answer	b) [If yes, to any Indigenous option in 4a] **Are you Status (Registered or Treaty Indian as defined by the *Indian Act* of Canada)?**<br>• Yes, Status Indian (Registered or Treaty)<br>• No<br>• Do not know<br>• Prefer not to answer	**Why are we asking this question?**<br><br>If you are Status, your provider may be able to refer you to more services.<br><br>You may be able to receive Non-Insured Health Benefits. This may include:<br>• some drugs<br>• vision care<br>• medical supplies<br>• medical transportation<br><br>**Understanding the question:**<br><br>**Status (Registered or Treaty Indian)** is a person registered under the Indian Act of Canada. People with status may receive health services under the Non-Insured Health Benefits Program.[1,2]<br><br>1. Government of Canada. (2022). About Indian Status. Accessed April 14, 2022<br>2. Statistics Canada. Registered or Treaty Indian status of person. 2021. Accessed April 11, 2022

<table>
<tr>
<td></td>
<td>c) If yes, Inuk/Inuit, are you a member of an Inuit land claims agreement?
• Yes
• No
• Not applicable, I am not an Indigenous person [remove if skip logic used]
• Do not know
• Prefer not to answer</td>
<td>c) [If yes, Inuk/Inuit in 4a] Are you a member of an Inuit land claims agreement?
• Yes
• No
• Do not know
• Prefer not to answer</td>
<td>Why are we asking this question?

If you are a member of an Inuit land claims agreement your provider may refer you to supports and services that may help you.

You may be able to receive Non-Insured Health Benefits. This may include:
• some drugs
• vision care
• medical supplies
• medical transportation

Understanding the Question:

A member of an Inuit land claims agreement is a beneficiary of: Nunavut Land Claim Agreement, or Inuvialuit Final Agreement.

You may have been automatically registered and received your territorial health care card.

If you live outside of the land claim settlement area you can register with a land claim organization to receive benefits.

1. Government of Canada. Your Health Benefits – A Guide for Inuit to Access Non-Insured Health Benefits. Accessed April 22, 2022.</td>
</tr>
<tr>
<td>4: Race</td>
<td>In our society, people are often described by their race or racial background. Our race may influence the way we are treated by individuals and institutions, and this may affect our health. Which category(ies) best describes you? Select all that apply:</td>
<td>Not Applicable</td>
<td>Why are we asking this question?

We are asking this question on race to help us know who we serve at the clinic. We're interested in monitoring the experience our clients have in the clinic to ensure that everyone has a positive experience regardless of their racial background.</td>
</tr>
</table>

	• Black (e.g., African, African Canadian, Afro-Caribbean descent)<br>• East Asian (e.g., Chinese, Japanese, Korean, Taiwanese descent)<br>• Indigenous (e.g., First Nations, Métis, Inuk/Inuit)<br>• Latin American (e.g., Hispanic or Latin American descent)<br>• Middle Eastern (e.g., Arab, Persian, West Asian descent (e.g., Afghan, Egyptian, Iranian, Kurdish, Lebanese, Turkish))<br>• South Asian (e.g., South Asian descent (e.g., Bangladeshi, Indian, Indo-Caribbean, Pakistani, Sri Lankan)<br>• Southeast Asian (e.g., Cambodian, Filipino, Indonesian, Thai, Vietnamese, or other Southeast Asian descent)<br>• White (e.g., European descent)<br>• Another race category (please specify) ________________<br>• Do not know<br>• Prefer not to answer		This information may also help us identify and address differences in health outcomes experienced by different racial groups.<br><br>**Understanding the question:**<br><br>**Race** is a term used to classify people into groups usually based on observable physical characteristics (for example, skin colour) but can also include characteristics such as accent or dress.[1,2,3]<br><br>Although these differences or groupings are created by the society we live in, racial categories can have significant consequences for people's lives, including unfair and unjust treatment by individuals and institutions. This is what we call racism.<br><br>Race should not be confused with ethnicity. **Ethnicity** refers to groups of people who share a common culture or ancestry. They may also share a common language, religion, geographic origin, nationality, cultural traditions, migration history or other commonalites.[1,2]<br><br>**Mixed race** individuals are encouraged to select more than one racial group.<br><br>If you are unsure what to select, you can choose "Another" and specify what racial group(s) best describe you.<br><br>This question was adapted from the March 2022 CIHI Race-based standards.[1]<br><br>1. Canadian Institute for Health Information (2022) Guidance on the Use of Standards for Race-Based and Indigenous Identity Data Collection and Health Reporting in Canada Ottawa, ON: CIHI. Accessed April 5, 2022

			2. Torontohealthequity.ca. Measuring Health Equity: Demographic Data Collection in Health Care. Accessed April 5, 2022<br>3. Government of Ontario Anti-Racism Directorate. Data Standards for the Identification and Monitoring of Systemic Racism. Accessed April 5, 2022
**5: People with Disabilities**	**Do you currently experience any of the following <u>due to a severe and persistent physical or mental condition</u>? Select all that apply:**<br><br>• Difficulty seeing (e.g., severe vision impairment)<br>• Difficulty hearing (e.g., severe hearing loss)<br>• Difficulty with walking or climbing (e.g., severe mobility issues)<br>• Difficulty remembering or with concentration (e.g., severe memory loss or disorientation)<br>• Difficulty with personal hygiene (e.g., physically unable or lack motivation to shower)<br>• Difficulty with activities for daily living (e.g., physically unable or lack motivation to: e.g., eat, get out of bed, work)<br>• Difficulty with communicating (e.g., severe speech impairment, trouble generating words)<br>• Difficulty with comprehension (e.g., severe learning disability, trouble understanding words)<br>• None of the above<br>• Do not know<br>• Prefer not to answer	Not Applicable	**Why are we asking this question?**<br>Your provider may use this to inform the care you receive.<br><br>Your provider may also use this to refer you to appropriate services that may be helpful to you.<br><br>This information may help us provide better accommodations for people living with disabilities. And this can improve their overall access to health care.<br><br>**Understanding the question:**<br>**“Severe and persistent condition”** is about long-term conditions or disabilities that affect day-to-day activities.<br><br>The condition **does not have to be diagnosed** by your provider.<br><br>***‘Difficulty seeing or hearing’*** is not asking about conditions that can be corrected with aids such as glasses or hearing aids.<br><br>***‘Difficulty with communication’*** is asking about people who have this difficulty because of a physical or mental condition and not because of other things such as language barriers or reading or writing level.

**6: Sex at Birth**	**What was your sex at birth?**<br>• Female<br>• Male<br>• Intersex<br>• Do not know<br>• Prefer not to answer	Not Applicable	**Why are we asking this question?**<br><br>This information lets us know who we serve at the clinic and how well we are meeting patient needs. This information can also help us screen for and prevent some health conditions.<br>This question gives you a space to share your identity and to help avoid assumptions about you or your healthcare.<br><br>**Understanding the question:**<br><br>**Sex at Birth** is often recorded at a person's birth and is usually based on a person's reproductive system and other physical traits.[1]<br><br>While **Gender Identity** is a person's sense of themselves relating to gender, be it male, female, a combination of both, or neither.[2]<br><br>Gender can be fluid and may change over time.[3]<br><br>**Intersex** refers to people born with differences in their reproductive features[2,4] that may not be easily characterized as male or female.[5]<br><br>1. Statistics Canada, Government of Canada (2021).Sex at birth of person Accessed April 18, 2022<br>2. Human Rights Campaign (2011). Glossary of Terms - Human Rights Campaign. Accessed April 18, 2022<br>3. Torontohealthequity.ca. Measuring Health Equity: Demographic Data Collection in Health Care. Accessed April 18, 2022<br>4. Ucdavis.edu. (2014). LGBTQIA Resource Center Glossary | LGBTQIA Resource Center. Accessed April 18, 2022<br>5. Ohrc.on.ca. (2014). Appendix B: Glossary for understanding gender identity and expression | Ontario Human Rights Commission (ohrc.on.ca). Accessed April 18, 2022

**7: Gender Identity**	**What is your gender identity?**<br>• Woman<br>• Man<br>• Transgender man<br>• Transgender woman<br>• Gender fluid or non-binary<br>• Two-Spirit (a term by and for Indigenous peoples)<br>• Prefer to self-describe______________<br>• Do not know<br>• Prefer not to answer	Not Applicable	**Why are we asking this question?**<br><br>This information lets us know who we serve at the clinic and how well we're meeting patient needs.<br><br>It helps us understand the range of gender identities that people at our clinic may experience.<br><br>This question gives you a space to share your identity and to help avoid assumptions about you or your healthcare.<br><br>**Understanding the question:**<br><br>**Gender Identity** is a person's sense of themselves relating to gender, be it male, female, a combination of both, or neither.[1]<br><br>Gender can be fluid and may change over time.[2]<br><br>**Sex at Birth** is often recorded at a person's birth and is usually based on a person's reproductive system and other physical traits.[3]<br><br>**Gender non-binary** describes a person who may experience a gender identity that is neither exclusively woman or man, is a combination of woman and man or is between or beyond genders.[4]<br>Non-binary can also be used as an umbrella term for identities that are gender fluid, moving between and through identities.[1]<br><br>**Two-Spirit** is a term used by Indigenous peoples to self-describe and express diverse sexual, gender and/or spiritual identities.[5] It is a way for Indigenous peoples to

			reconnect with their traditional languages, ways, and cultures.[6]<br><br>**Transgender** or trans is an umbrella term for people whose gender identity or expression is different from conventional or cultural expectations based on the sex they were assigned at birth.[7,8]<br><br>1. Ohrc.on.ca. (2014). Appendix B: Glossary for understanding gender identity and expression | Ontario Human Rights Commission. Accessed April 18, 2022<br>2. Torontohealthequity.ca. Measuring Health Equity: Demographic Data Collection in Health Care. Accessed April 18, 2022<br>3. Statistics Canada, Government of Canada (2021).Sex at birth of person. Accessed April 18, 2022<br>4. Losty, M. and O'Connor, J. (2017). Falling outside of the 'nice little binary box': a psychoanalytic exploration of the non-binary gender identity. *Psychoanalytic Psychotherapy*, 32(1), pp.40–60<br>5. Knudson, S. and Hahn, D. (2019). *Committing sociology: critical perspectives on our social world.* Toronto: Pearson Canada.<br>6. Fewster, P.H. (2018). Researching for LGBTQ Health. Lgbtqhealth.ca. Accessed April 18, 2022<br>7. Ucdavis.edu. (2014). LGBTQIA Resource Center Glossary | LGBTQIA Resource Center. Accessed April 18, 2022<br>8. Human Rights Campaign (2011). Glossary of Terms - Human Rights Campaign. Accessed April 18, 2022
**8: Sexual Orientation**	**Which category(ies) best describe your sexual orientation? Select all that apply:**<br>• Asexual or Aromantic<br>• Bisexual<br>• Demisexual<br>• Homosexual or Gay<br>• Heterosexual or Straight<br>• Lesbian<br>• Pansexual<br>• Queer<br>• Two-Spirit (a term by and for Indigenous peoples)	Not Applicable	**Why are we asking this question?**<br><br>This information lets us know who we serve at the clinic. It helps us understand the range of orientations that people at our clinic may experience.<br><br>This question gives you a space to share your orientation and to help avoid assumptions about you or your healthcare.<br><br>**Understanding the question:**

	• Prefer to self-describe______________<br>• Do not know<br>• Prefer not to answer		**Sexual orientation** is a term for the emotional, physical, romantic, sexual, and spiritual attraction, non-attraction, desire, or affection for another person.[1]<br><br>**Aromantic** describes a romantic identity for people who do not experience romantic attraction to people of any gender but may still have sexual attraction to other people.[2]<br><br>**Asexual** describes people who do not experience sexual attraction to people of any gender but may still experience romantic attractions to other people.[1,3]<br><br>**Bisexual** describes a person whose primary emotional, romantic, or sexual attraction is toward people of both the same and different gender.<br><br>**Demisexual** describes a person who feels sexual attraction only to people that they have an emotional bond with.[3]<br><br>**Homosexual or Gay** describes a person whose primary emotional, romantic, or sexual attraction is toward people of the same gender.[1]<br><br>**Heterosexual or Straight** describes a person whose primary emotional, romantic, or sexual attraction is toward people of an opposite gender.<br><br>**Lesbian** describes a person, usually a woman, whose primary emotional, romantic, or sexual attraction is toward people of the same gender.[1]<br><br>**Pansexual** describes a person who has emotional, romantic, or sexual desire for people of all genders and sexes.[1]

			**Queer** is a term used to express a range of identities and orientations that are beyond the mainstream.[1]<br><br>**Two-Spirit** is a term used by Indigenous peoples to self-describe and express diverse sexual identities as having both a masculine and feminine spirit.[4] It is a way for Indigenous peoples to reconnect with their traditional languages, ways, and cultures.[5]<br><br>1. Human Rights Campaign (2011). Glossary of Terms - Human Rights Campaign. Accessed April 18, 2022<br>2. Suen, L.W., Lunn, M.R., Katuzny, K., … & Obedin-Maliver, J. (2020). What Sexual and Gender Minority People Want Researchers to Know About Sexual Orientation and Gender Identity Questions: A Qualitative Study. *Archives of Sexual Behavior,* 49(7), pp.2301–2318.<br>3. Ucdavis.edu. (2014). LGBTQIA Resource Center Glossary | LGBTQIA Resource Center. Accessed April 18, 2022<br>4. Knudson, S. and Hahn, D. (2019). *Committing sociology: critical perspectives on our social world.* Toronto: Pearson Canada.<br>5. Fewster, P.H. (2018). Researching for LGBTQ Health. Lgbtqhealth.ca. Accessed April 18, 2022
Social Needs			
**9: Education**	**What is your current level of education?**<br><br>• No formal schooling<br>• Grade school (grade 1-8)<br>• Some high school, but did not graduate<br>• High school or high school equivalency certificate (grade 9-12)<br>• Completed Registered Apprenticeship or other trades certificate or diploma (or ongoing)	Not Applicable	**Why are we asking this question?**<br><br>Your provider may use this information to know how well you are able to read and understand health information such as medication labels and instructions.[1]<br><br>Your provider may also use this to connect you with supports that may be helpful to you.<br><br>A person's education level is related to their ability to understand basic health information and access health services. And these may affect their health.[1]<br><br>**Understanding the question:**

	• College, CEGEP or other non-university certificate or diploma (or ongoing)<br>• Undergraduate degree or some university<br>• Postgraduate degree or professional designation (e.g., Master's, PhD, MD)<br>• Do not know<br>• Prefer not to answer		The term ***'some'*** in '***Some high school***' means your high school education may be in progress or stopped.<br><br>The term ***'ongoing'*** means schooling or training is in progress.<br><br>If you received your education by home-schooling, please select the matching grade level you have completed.<br><br>1. Canadian Institute for Health Information (2018) In Pursuit of Health Equity: Defining Stratifies for Measuring Health Inequality — A Focus on Age, Sex, Gender, Income, Education and Geographic Location. Ottawa, ON: CIHI. Accessed April 4, 2022
**10: Income/ Finances**	**Do you currently have difficulty paying for basic needs?**<br>• Yes<br>• No<br>• Not applicable, I do not have to pay for basic needs<br>• Do not know<br>• Prefer not to answer	Not Applicable	**Why are we asking this question?**<br><br>Your provider may use this to refer you to income resources and supports that may be helpful to you.<br><br>Low income has been shown to negatively affect mental and physical health.[1-4]<br><br>**Understanding the question:**<br><br>**Basic Needs** are things that a person requires to achieve and maintain physical and mental well-being and include food, water, shelter, and clothing.[2-4]<br><br>1. National Association of Community Health Centre, Inc, Association of Asian Pacific Community Health Organizations, and the Oregon primary Care Association. (2019). PRAPARE. Protocol for Responding to and Assessing Patients' Assets, Risks and Experiences. Implementation and Action Toolkit. Accessed April 14, 2022.<br>2. Collin, C. and Campbell, B. (2008). Measuring Poverty: A Challenge for Canada Accessed April 14, 2022<br>3. Sarlo C. (2013). Poverty: Where do we draw the line? Accessed April 13, 2022<br>4. Homeless Hub. (2021). Basic Needs. Accessed April 13, 2022

**11: Food Security**	**Please respond to the following statements:**<br><br>**a) "Within the past 12 months, we worried whether our food would run out before we could buy or get more"**<br>• Often True<br>• Sometimes true<br>• Never true<br>• Do not know<br>• Prefer not Answer<br><br>b) **"Within the past 12 months, the food we bought just didn't last and we could not buy or get more."**<br>• Often True<br>• Sometimes true<br>• Never true<br>• Do not know<br>• Prefer not Answer	Not Applicable	**Why are we asking this question?**<br><br>Your provider may use this to refer you to resources and supports that may help you obtain food and maintain a healthy diet.[1,2]<br><br>Your provider may also use this to give you advice on regular eating and nutrition.[1]<br><br>Not having enough food can mean choosing less expensive, more filling, and less healthy food. This can negatively affect your health.[2]<br><br>This question was adapted from the Hunger Vital Sign™ screening tool.[3]<br><br>1. National Association of Community Health Centre, Inc, Association of Asian Pacific Community Health Organizations, and the Oregon primary Care Association. (2019). PRAPARE. Protocol for Responding to and Assessing Patients' Assets, Risks and Experiences. Implementation and Action Toolkit. Accessed April 14, 2022<br>2. Botelho, FC., Junior, IF., Guerra, LD., Rodrigues, SF., Tonacio, LV. (2020). Scientific literature on food and nutrition security in primary health care: A scoping review. Global Public Health, 15(12), pp. 1902-1916.<br>3. Hager, E.R., Quigg, A.M., Black, M.M., … & Cook, J.T., de Cuba, S.A.E., Casey, P.H., Chilton, M. and Cutts, D.B., 2010. Development and validity of a 2-item screen to identify families at risk for food insecurity. *Pediatrics*, *126*(1), pp.e26-e32.
**12: Medication Access**	**In the past 12 months, were you unable to get medicine or medical supplies, or did you do anything to make them last longer *because of the cost*?**<br>• Yes<br>• No	Not Applicable	**Why are we asking this question?**<br><br>Your provider may use this to refer you to resources that can help you access medicine or medical supplies.<br><br>This may also help your provider consider lower cost or free medicine options.

	• Not applicable, I did not have to get any medicine or medical supplies in the past 12 months<br>• Do not know<br>• Prefer not to answer		Being able to access and use needed medicine and medical supplies can affect your health.[1]<br><br>**Understanding the question:**<br><br>**'Medicine'** includes both prescription and over-the-counter medicines.<br><br>**'Medical supplies'** include any equipment you may need to manage your health or disability. For example, needles, glucose monitors, walking aids.<br><br>1. Sobeski, L. M., Schumacher, C. A., Alvarez, N. A., ... & Van Dril, E. (2021). Medication access: Policy and practice opportunities for pharmacists. *Journal of the American College of Clinical Pharmacy*, *4*(1), 113-125.
**13: Housing**	**a) What is your current housing situation?**<br>• A place you or your family owns<br>• A place you or your family rents<br>• Social housing, Subsidized housing or Rent-geared-to-income<br>• Supportive housing or Group Home<br>• Long-term care facility<br>• Correctional facility<br>• Staying in someone else's place because you have no alternative<br>• Experiencing homelessness (e.g., shelter, living in a public place or vehicle)<br>• Other (Specify)________________<br>• Do not know<br>• Prefer not to answer	Not Applicable	**Why are we asking this question?**<br><br>Your provider may use this to refer you to resources and supports that may help you with housing needs.<br><br>It is known that unstable housing can negatively affect people's health.[1,2]<br><br>**Understanding the question:**<br><br>**Social housing** is housing that is subsidized or partly paid for by a level of government.[3]<br><br>**Subsidized housing** means receiving help paying for housing from the government or a private organization.[4]<br><br>**Rent-geared-to-income** is a type of rent assistance. It lets a person pay a percentage of their income for rent.[5]

			**Supportive housing** refers to housing with on-site staff who provide residents with ongoing support with their needs.<br><br>1. Krieger, J. and Higgins, D.L. (2002). Housing and Health: Time Again for Public Health Action. American Journal of Public Health, 92(5), pp.758–768.<br>2. Office of Disease Prevention and Health Promotion (2014). Housing Instability | Healthy People 2020 Healthypeople.gov. Accessed April 7, 2022<br>3. www.homelesshub.ca. Affordable Housing | The Homeless Hub. Accessed April 7, 2022<br>4. Settlement.org. (2018). What is subsidized housing? Accessed April 7, 2022<br>5. www.durham.ca. (2021). Rent-Geared-to-Income Housing (RGI) - Region of Durham . Accessed April 7, 2022
	**b) Who do you live with? Select all that apply:**<br>• Parent(s) or Guardian(s)<br>• Spouse or Partner<br>• Child(ren)<br>• Grandparent(s)<br>• Sibling(s)<br>• Other family<br>• Friends or Roommates<br>• Paid caregiver or attendant<br>• Alone<br>• Other (Specify)<br>____________________<br>• Do not know<br>• Prefer not to answer	Not Applicable	**Why are we asking this question?**<br><br>This information may help your provider further understand your living situation and the social supports that may be available to you.<br><br>This may also help provide information on aspects of your overall health.<br><br>It is known that living with others can impact your health.[1,2]<br><br>1. Krieger, J. and Higgins, D.L. (2002). Housing and Health: Time Again for Public Health Action. American Journal of Public Health, 92(5), pp.758–768.<br>2. Office of Disease Prevention and Health Promotion (2014). Housing Instability | Healthy People 2020 Healthypeople.gov. Accessed April 7, 2022
	**c) In the past 12 months, was there a time when you were not able to pay the mortgage or rent on time?**<br>• Yes<br>• No	Not Applicable	**Why are we asking this question?**<br><br>Your provider may use this to refer you to housing or financial resources and supports that may be helpful to you.

	• Not applicable, I do not have to pay rent or mortgage<br>• Do not know<br>• Prefer not to answer		Not being able to pay your mortgage or rent on time may negatively affect your health (for example, it may cause stress).[1,2]<br><br>1. Krieger, J. and Higgins, D.L. (2002). Housing and Health: Time Again for Public Health Action. American Journal of Public Health, 92(5), pp.758–768.<br>2. Office of Disease Prevention and Health Promotion (2014). Housing Instability | Healthy People 2020 Healthypeople.gov. Accessed April 7, 2022
**14: Transportation**	**In the past 12 months, has lack of transportation kept you from medical appointments, meetings, work, or from getting things needed for daily living? Select all that apply:**<br>• Yes, it has kept me from medical appointments or getting medicines<br>• Yes, it has kept me from non-medical meetings, appointments, work, or getting things that I need<br>• No<br>• Not applicable, I did not need transportation for these activities in the past 12 months<br>• Do not know<br>• Prefer not to answer	Not Applicable	**Why are we asking this question?**<br><br>Your provider may use this to refer you to transportation resources and supports.<br><br>Lack of transportation can impact your health.[1-4] It may make it hard to get to a healthcare appointment on time. It can also make it hard to get to and from work, get healthy food, use medicine on time, or get something you need.<br><br>**Understanding the question:**<br><br>**"Medical Appointments"** are appointments to meet your health needs. This may include an appointment with your doctor(s), specialist, or social worker. This also includes getting medicine from your doctor or pharmacist.[1]<br><br>You might lack transportation because of:[4]<br>• cost and access,<br>• distance,<br>• gas or parking costs,<br>• public transportation safety,<br>• and/or other reasons.

			**"Things needed for daily living"** are things such as groceries or personal products that you need to be healthy.<br><br>This question was adapted from the PREPARE tool.[1]<br><br>1. National Association of Community Health Centre, Inc, Association of Asian Pacific Community Health Organizations, and the Oregon primary Care Association. (2019). PRAPARE. Protocol for Responding to and Assessing Patients' Assets, Risks and Experiences. Implementation and Action Toolkit. Accessed April 14, 2022<br>2. Billioux, A., Verlander, K., Anthony, S., Alley, D. Standardized screening for health-related social needs in clinical settings: the accountable health communities screening tool. Discussion Paper, National Academy of Medicine, Washington, DC. Accessed April 14, 2022<br>3. Razon, N., Gottlieb, L. (2022). Content Analysis of Transportation Screening Questions in Social Risk Assessment Tools: Are we capturing transportation insecurity. Journal of the American Board of Family Medicine, 35(2), pp.400-405.<br>4. Syed, ST., Gerber, BS., Shapr, LK. (2013). Traveling toward disease" transportation barriers to health care access. J community Health, 38(5), pp. 976-993.
**15: Phone and Internet Access**	**Do you currently have consistent access to a phone or the internet?**<br>• Yes, phone only<br>• Yes, internet only<br>• Yes, both<br>• No<br>• Do not know<br>• Prefer not to answer	Not Applicable	**Why are we asking this question?**<br><br>Your provider may use this to refer you to resources that can improve your access to phone and internet services.<br><br>These resources may help improve your access to health care appointments and health information.<br><br>This information may also inform how you receive health care services. For example, by phone, video, or in-person.<br><br>Access to a phone and internet is now a basic human right. This is due to its impact on access to health care and other needed services.[1,2]

			**Understanding the question:**<br><br>**“Consistent access”** means having regular, reliable, and adequate access to a phone or internet to obtain needed services. This includes access to a phone or internet for a healthcare appointment.[3] This also includes access to health information that is on the internet that your provider refers you to.[3]<br><br>1. Benda, NC., Veinot, TC., Sieck, C., Ancker, JS. (2020). Broadband internet access is a social determinant of Health! AM J Public Health, 110(8), pp. 1123-1125. doi:<br>2. Rubin, R., 2021. Internet access as a social determinant of health. *JAMA*, *326*(4), pp.298-298.<br>3. Graves, JM., Abshire, DA., Amiri, S., Mackelprang, JL. (2021). Disparities in technology and broadband internet access across rurality: implications for health and education. Fam Community Health, 44(4), pp. 257-265.
**16: Utilities**	**In the past 12 months, did you miss making a payment on any utility bills (e.g., electric, gas/oil, water) *because of cost*?**<br>• Yes<br>• No<br>• Not applicable, I did not have to pay utility bills in the past 12 months or utilities already included in rent<br>• Do not know<br>• Prefer not to answer	Not Applicable	**Why are we asking this question?**<br><br>Your provider may use this to refer you to supports that may be helpful to you.<br><br>Difficulty paying for utilities can negatively affect your health.[1] For example, you need heat when it is cold outside.[2]<br><br>Difficulty paying utility bills can also be a sign of financial need, which can affect your health (for example, it may cause stress).<br><br>**Understanding the question:**<br><br>This question is asking about basic utilities. Basic utilities may include:<br>• heating or cooling<br>• electricity or hydro<br>• water or sewer

			• phone, and internet.<br><br>The specific utilities you pay for may depend on where you live. For example, you may heat your home using natural gas, furnace oil or wood depending on where you live.<br><br>1. International Policy Centre for Inclusive Growth (2009) Equitable access to basic utilities: Public versus private provision and beyond. Accessed: April 20, 2022.<br>2. Grey, C. N., Jiang, S., Nascimento, C., Rodgers, S. E., Johnson, R., Lyons, R. A., & Poortinga, W. (2017). The short-term health and psychosocial impacts of domestic energy efficiency investments in low-income areas: a controlled before and after study. BMC Public Health, 17(1), 1-10.
**17: Social Supports**	**a) Do you feel you have people who you can open up to or confide in?**<br>• Yes, I always or sometimes have someone<br>• No, I don't have anyone<br>• Do not know<br>• Prefer not to answer<br><br>**b) Do you have people to rely on if you needed help?**<br>• Yes, I always or sometimes have someone<br>• No, I don't have anyone<br>• Do not know<br>• Prefer not to answer	Not Applicable	**Why are we asking this question?**<br><br>Your provider may use this to refer you to social resources and social supports that may be helpful to you.<br><br>**Understanding the question**<br><br>Having "**people you feel you can open up to or confide in**" means being able to talk to people that you care about, trust and feel close to.[1]<br>Having social relationships and feeling connected can impact health.[1]<br><br>**b. Why are we asking this question?**<br>Your provider may refer you to social resources and social supports that may be helpful to you.<br>Having **"people you can rely on"** can help you to feel more socially connected and secure, and this may impact your health.<br><br>1. National Association of Community Health Centre, Inc, Association of Asian Pacific Community Health Organizations, and the Oregon primary Care Association. (2019). PRAPARE. Protocol for Responding to and Assessing Patients' Assets, Risks

			and Experiences. Implementation and Action Toolkit. Accessed April 14, 2022
**18: Employment**	**a) Are you currently employed (this includes self-employed, full-time, part-time or other)?**<br>• Yes<br>• No<br>• Do not know<br>• Prefer not to answer	**a) Are you currently employed (this includes self-employed, full-time, part-time or other)?**<br>• Yes → Go to question 18c<br>• No → Go to question 18b<br>• Do not know → Go to question 18b<br>• Prefer not to answer → Go to question 18b	**Why are we asking this question?**<br><br>Your provider may use this information to better understand employment circumstances that could be affecting your health. Your provider may also refer you to employment resources that could be helpful. It is known that those who work tend to have better health than those who are unemployed.[1]<br><br>1. Waddell, G., & Burton, A. K. (2006). Is work good for your health and well-being? Accessed April 4, 2022.
	**b) Are you currently looking for work?**<br>• Yes<br>• No<br>• Not applicable, I am currently employed [remove if skip logic is used]<br>• Do not know<br>• Prefer not to answer	b) [If no is selected in 18a] **Are you currently looking for work?**<br>• Yes → Go to question 19<br>• No → Go to question 19<br>• Do not know → Go to question 19<br>• Prefer not to answer → Go to question 19	**Why are we asking this question?**<br><br>Your provider may use this information to refer you to employment resources that may be helpful.
	c) **Is your main job temporary or part-time (e.g., casual, contract, freelance, short-term, seasonal)?**<br>• Yes<br>• No<br>• Not applicable, I am not currently employed [remove if skip logic is used]<br>• Do not know<br>• Prefer not to answer	c) [If yes is selected in 18a] **Is your main job temporary or part-time (e.g., casual, contract, freelance, short-term, seasonal)?**<br>• Yes → Go to question 18d<br>• No → Go to question 18d<br>• Do not know → Go to question 18d<br>• Prefer not to answer → Go to question 18d	**Why are we asking this question?**<br><br>Your provider may use this information to better understand employment circumstances that may be affecting your health.<br><br>Your provider may also refer you to employment resources that could be helpful.<br><br>It is known that unstable or unpredictable work negatively affects peoples' health.[1,2]

			**Understanding the question:**<br><br>**"Main job"**: If you have more than one job, your main job is your primary source of income.<br><br>1. Benach, J., Vives, A., Amable, M., Vanroelen, C., Tarafa, G. and Muntaner, C., 2014. Precarious employment: understanding an emerging social determinant of health. *Annual review of public health*, *35*, pp.229-253.<br>2. Benach, J., Vives, A., Tarafa, G., Delclos, C. and Muntaner, C., 2016. What should we know about precarious employment and health in 2025? Framing the agenda for the next decade of research. *International journal of epidemiology*, *45*(1), pp.232-238.
	**d) Do you feel that your current employment could be negatively affected if you raised concerns about your work (e.g., health, safety, rights)?**<br>• Yes<br>• No<br>• Not applicable, I am not currently employed [remove if skip logic is used]<br>• Do not know<br>• Prefer not to answer	[If yes is selected in 18a] **Do you feel that your current employment could be negatively affected if you raised concerns about your work (e.g., health, safety, rights)?**<br>• Yes → Go to question 18e<br>• No → Go to question 18e<br>• Do not know → Go to question 18e<br>• Prefer not to answer → Go to question 18e	**Why are we asking this question?**<br><br>Your provider may use this information to better understand employment circumstances that may be affecting your health.<br><br>Your provider may also refer you to employment resources that could be helpful.<br><br>A person's inability or unwillingness to raise concerns about health, safety and rights at work can negatively affect their health.[1]<br><br>**Understanding the question:**<br><br>**Health and safety concerns** at work may include concerns about unsafe work (for example, lifting items that are too heavy, working with violent clients), unsafe work environments (for example, slippery floors, broken machinery), or harassment due to race, gender, or sexual orientation.[1]

			**Concerns about employment rights** may include questions about your right to breaks, sick leave or vacation time.[1]<br><br>Your **health and safety rights**[1] include your right to**:**<br>• know about unsafe work<br>• refuse unsafe work<br>• participate in workplace health and safety decisions<br><br>1. Lewchuk, W., 2013. The limits of voice: Are workers afraid to express their health and safety rights. Osgoode Hall LJ, 50(4), p. 789-812. Accessed April 20, 2022
	e) **In the past 12 months, did your income change a lot from month to month?**<br>• Yes<br>• No<br>• Not applicable, I am not currently employed [remove if skip logic is used]<br>• Do not know<br>• Prefer not to answer	[If yes is selected in 18a] **In the past 12 months, did your income change a lot from month to month?**<br>• Yes → Go to question 19<br>• No → Go to question 19<br>• Do not know → Go to question 19<br>• Prefer not to answer → Go to question 19	**Why are we asking this question?**<br><br>Your provider may use this information to understand employment conditions that could be affecting your health.<br><br>Your provider may also refer you to employment or income resources that could be helpful.<br><br>Changing income is a key feature of unstable or unpredictable work. This is known to have negative effects on health[1].<br><br>1. Lewchuk, W., 2012. The limits of voice: Are workers afraid to express their health and safety rights. Osgoode Hall LJ, 50, p. 789-812. Accessed April 20, 2022
Optional Questions			
**1: Ethnicity**	**What is your ethnic or cultural background?** e.g., Chinese, Filipino, Guyanese, Scottish, Somali, Korean<br><br>__________________	Not Applicable	**Why are we asking this question?**<br>This question gives people a space to share their ethnic or cultural identity. This information lets us know who

			we serve at the clinic and may help us understand and address the unique needs of our patients.<br><br>**Understanding the question:**<br>This question on ethnicity should not be confused with Race. **Ethnicity** refers to groups of people who share a common culture or ancestry. They may also share a common language, religion, geographic origin, nationality, cultural traditions, migration history and so on.[1,2]<br><br>**Race** is a term used to classify people into groups usually based on observable physical characteristics (e.g., skin colour) but can also include characteristics such as accent, dress and so on.[1,2,3]<br><br>Although these differences or groupings are created by the society we live in, racial categories can have significant consequences for people's lives, including unfair and unjust treatment by individuals and institutions. This is what we call racism.<br><br>We welcome you to share the ethnic group or culture you identify with.<br><br>1. Canadian Institute for Health Information (2022) Guidance on the Use of Standards for Race-Based and Indigenous Identity Data Collection and Health Reporting in Canada Ottawa, ON: CIHI. Accessed April 5, 2022<br>2. Torontohealthequity.ca. Measuring Health Equity: Demographic Data Collection in Health Care. Accessed April 5, 2022<br>3. Government of Ontario Anti-Racism Directorate. Data Standards for the Identification and Monitoring of Systemic Racism. Accessed April 5, 2022
**2: Religion**	**What is your religious or spiritual affiliation? Select all that apply:**	Not Applicable	**Why are we asking this question?**

	• Agnosticism<br>• Animism or Shamanism<br>• Atheism<br>• Baha'i Faith<br>• Buddhism<br>• Christian Orthodox<br>• Christian, *not included elsewhere on this list*<br>• Confucianism<br>• Hinduism<br>• Islam<br>• Jainism<br>• Judaism<br>• Native Spirituality<br>• Pagan<br>• Protestant<br>• Rastafarianism<br>• Roman Catholic<br>• Sikhism<br>• Spiritual<br>• Unitarianism<br>• Zoroastrianism<br>• Other (Specify) ____________________<br>• Not Applicable, I do not have a religious or spiritual affiliation<br>• Do not know<br>• Prefer not to answer		Your provider may use this to better understand how your religious or spiritual beliefs and practices influence your health or health behaviors. This may include your diet, holidays you observe, or the health care treatment options you feel comfortable with.<br><br>Your provider may use this to inform the care you receive.<br><br>It is known that religious affiliation or involvement can positively affect health[1].<br><br>**Understanding the question:**<br><br>"**Religious or spiritual affiliation**" is about your connection with a particular set of beliefs, attitudes, or practices. This may or may not be related to a specific religion (for example, Catholicism, Islam) or belief in a higher being.[1] This also relates to a person's sense of spirituality.<br><br>Select the religion(s) you feel connected to. This includes a religion you are actively practicing, your family raised you in, or you feel close to.<br><br>You can select more than one option. If you do not find your religion listed, you can write it in.<br><br>1. Chatters, L. (2000) 'Religion and Health: Public health research and practice,' Annual Review of Public Health, 21, pp. 335-367. Doi:

Implementing the SPARK Tool: Collecting Sociodemographic Data in Health Care

SPARK: Screening for Poverty And Related social determinants and intervening to improve Knowledge of and links to resources

Acknowledgement
This training module was developed by the SPARK Study and the Upstream Lab at St. Michael's Hospital, Unity Health Toronto, Toronto, Ontario and formatted using Rise360 by Olivia Neale, University of Toronto.

Its content was adapted from the “Measuring Health Equity in Toronto Central Local Health Integration Network (TC LHIN)” Training Model, which was developed by the Human Rights & Health Equity Office, Sinai Health System, with support from TC LHIN.

More information available at www.torontohealthequity.ca

We acknowledge that Indigenous peoples are the traditional guardians of this land that we call Canada in which we gather. We acknowledge the historical oppression of lands, cultures and the original Peoples of this country and know we have a role to play in the path to decolonization that we share together. We recognize our duty to fight for Indigenous rights to be restored and commit ourselves to the journey of healing.

Learning Objectives

- Sociodemographic
- Health Equity
- Examining Health Equity
- The SPARK Project
- The SPARK Tool Questions
- Social Needs Resources for Patients and Providers
- Introducing the SPARK Tool to Patients
- Role Playing - How Would You Respond?
- Implementation Resources and Materials
- Studying the SPARK Tool Implementation
- Wrap-Up
- Help Us Improve this Training Module
- Thank You

Learning Objectives

1. Develop knowledge of health equity principles, the difference between health equity and inequity, and sociodemographic data

2. Become familiar with the SPARK Implementation Study and the SPARK Tool survey questions

3. Understand best practices for introducing the SPARK Tool survey to patients and responding to their questions

This training module will prepare you to introduce the SPARK Tool in your clinic.

The self-administered SPARK Tool is a survey, to be completed by patients of your clinic, to collect sociodemographic data.

CONTINUE

Sociodemographic

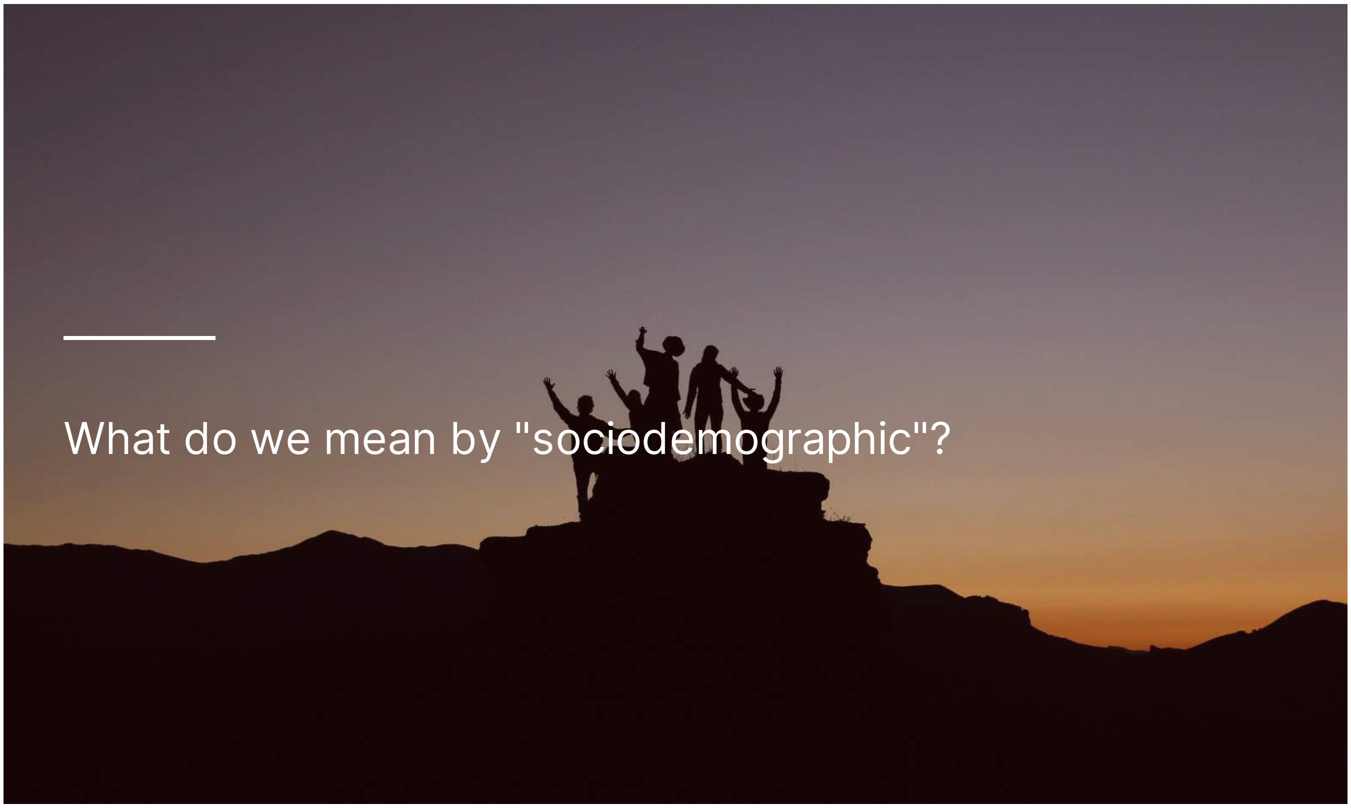

“Sociodemographic” data is information about a group of people that helps to describe them.

Examples of sociodemographic information are:

- Language

- Racial/Ethnic group
- Disability
- Sexual Orientation
- Gender
- Income

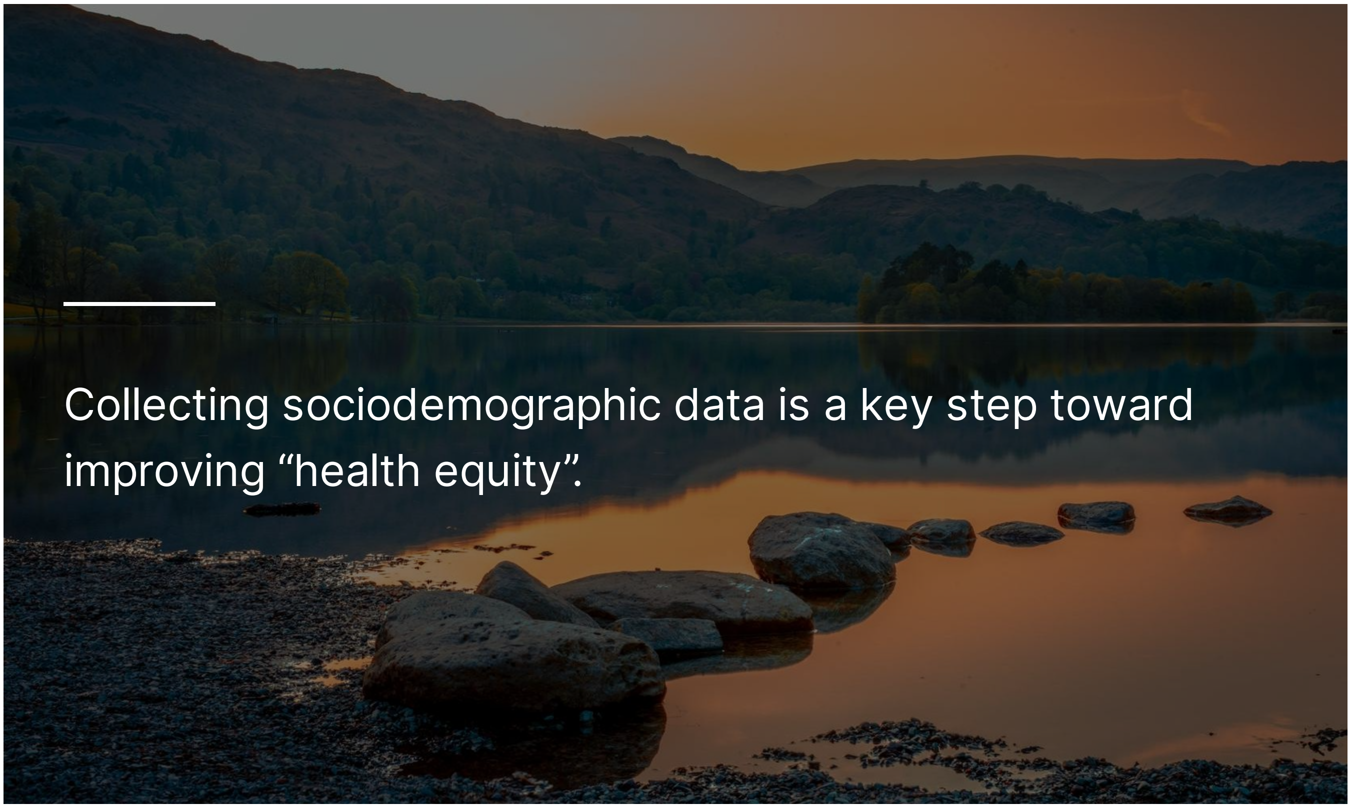

CONTINUE

Health Equity

What is “health equity”?

> *Health equity means that all people can:*
>
> - *Reach their full health potential*
> - *Receive high quality care that is fair and appropriate from each person's perspective*
>
> *This can be done no matter where they live, who they are, or what they have."*

Health Quality Ontario, 2016

Equal care does not equal **Equitable care**

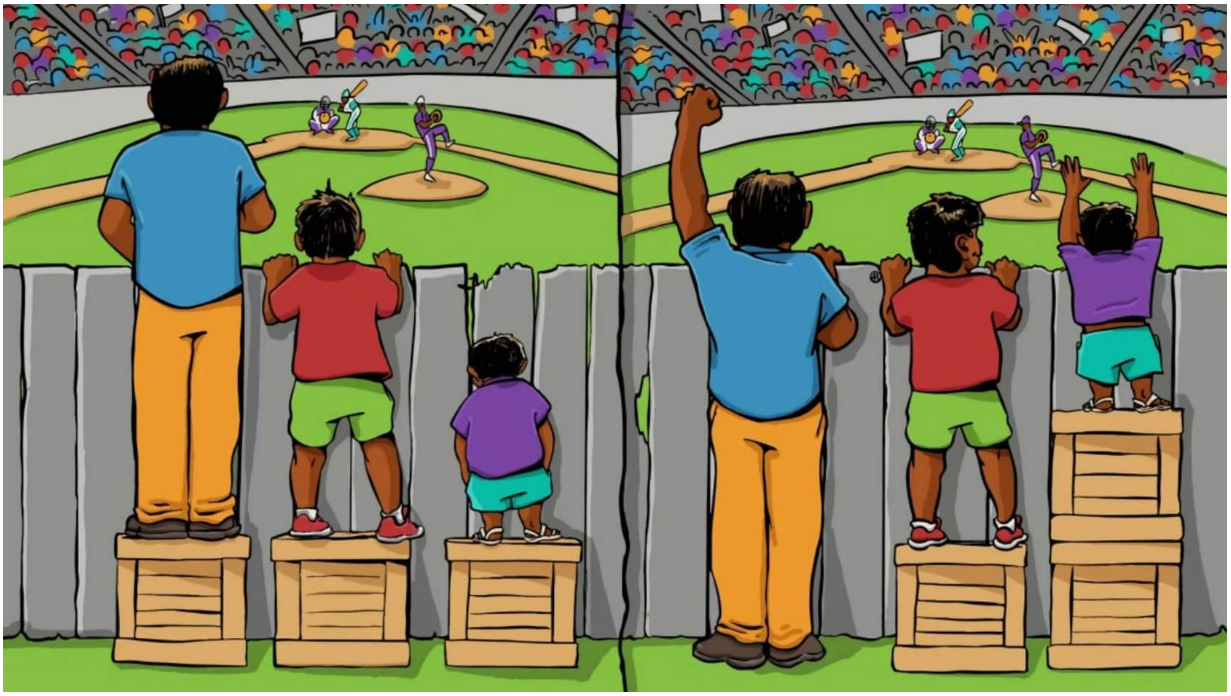

Gutoskey, E (2020). The young man in the purple knows just how important the difference between equality and equity can be. / Angus Maguire // Interaction Institute for Social Change. Mental Floss. https://www.mentalfloss.com/article/625404/equity-vs-equality-what-is-the-difference

---

Health equity is achieved when everyone using the healthcare system has **the same opportunity** for positive outcomes.

Equality presumes that everyone is the same.

Rather than thinking about everyone as the same, equity recognizes differences amongst certain groups of people which may impact their access to care.

Health Equity and Social Determinants of Health

- When we talk about health equity, it’s important to understand social determinants of health.
- These are systematic social and economic conditions that influence a person’s health.
- Social determinants of health include factors such as access to housing, healthcare, education, or a living wage
- Other social determinants of health include race, gender identity, disability, and availability of social supports

According to the World Health Organization and the Canadian Medical Association, **social determinants impact individual and population health more than biological and environmental conditions and access to the healthcare system.**

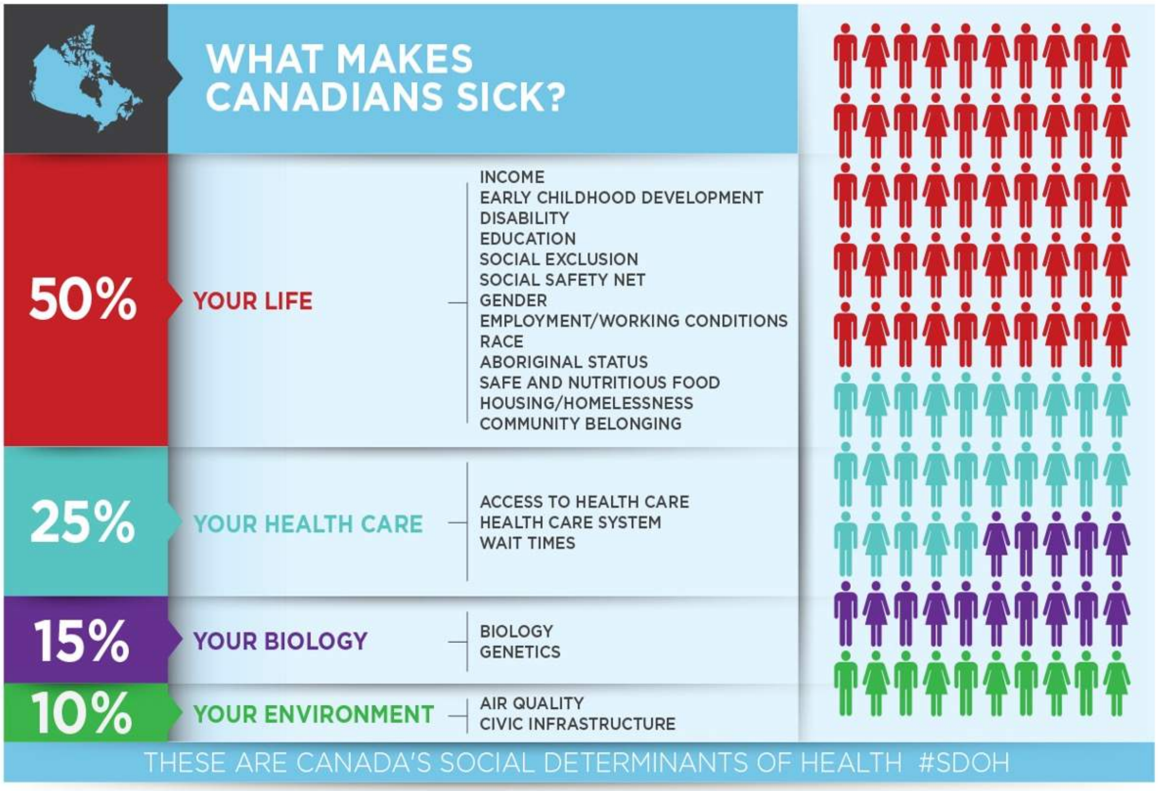

Canadian Medical Association

CONTINUE

Examining Health Equity

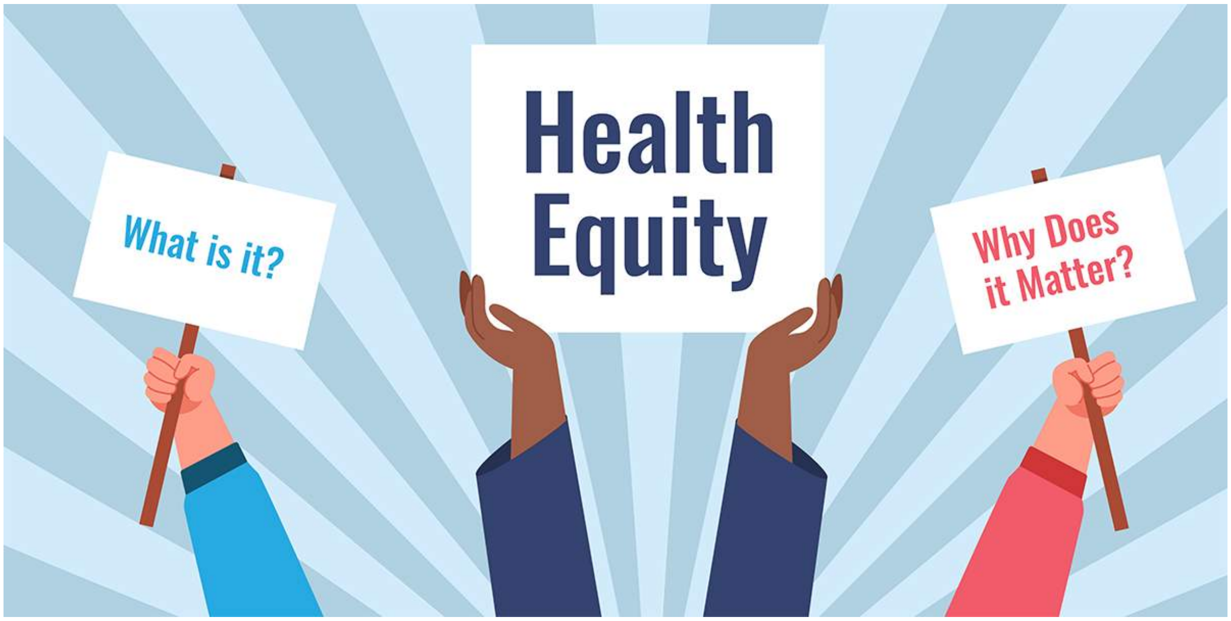

Audacious Inquiry (2021). Health Equity and Health Disparities: The Role of Health IT and the Need to Standardize Data Collection. https://ainq.com/health-equity-role-of-health-it/

Examining Health Equity

A way to measure health equity is to collect sociodemographic data. You will now watch a video on "Measuring Health Equity".

This video was generated for a previous sociodemographic data collection study in Toronto using the 8 “Health Equity Questions,” that serve as the basis for the SPARK Tool.

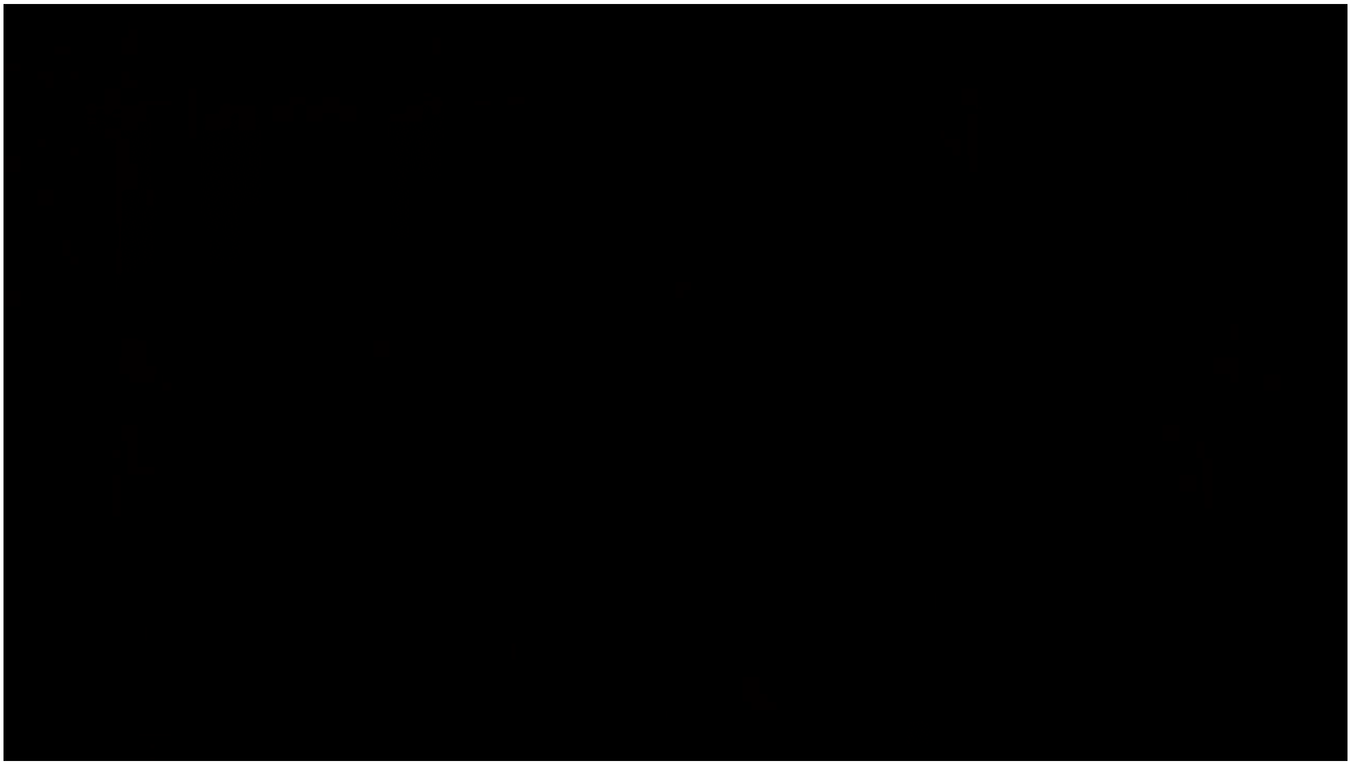

Complete the content above before moving on.

Now that you’ve watched the video, **let’s see what you’ve learned** about social determinants of health and health inequities.

New Understanding 1

Access to a living wage* does not affect health, it is what we do to take care of ourselves that determines whether we get sick or not.

**A 'living wage' is defined as the hourly pay a worker needs to earn to cover their basic expenses and participate in their community*

- ◯ True
- ◯ False

SUBMIT

New Understanding 2

Some children in Canada experience poorer health than others due to their socio-economic status. Children from lower income families are more likely to have lower birth rates, poorer oral health, and poorer nutrition.

- ○ True
- ○ False

SUBMIT

New Understanding 3

In Canada, living with a disability does not significantly impact experiences in the healthcare system.

- ○ True

False

SUBMIT

New Understanding 4

People seeking mental health supports are a small proportion of the population and are usually able to obtain effective healthcare.

True

False

SUBMIT

New Understanding 5

People may not feel comfortable answering questions from healthcare providers and staff based on previous experiences of discrimination and harassment in their daily lives.

- ○ True
- ○ False

SUBMIT

Complete the content above before moving on.

The SPARK Project

Overview of the SPARK Project

The Screening for Poverty And Related social determinants and intervening to improve Knowledge of and links to resources (SPARK) project is a national project that is being conducted in 5 provinces in Canada (Ontario, Manitoba, Newfoundland and Labrador, Nova Scotia and Saskatchewan). **The goal of the SPARK project** is to develop and implement a standardized tool for collecting sociodemographic data in primary healthcare settings. The SPARK tool is an updated version of the 2012 Health Equity Questions. It has 18 core questions and 2 optional questions.

Please note there may be some differences in the Tool questions used at your clinic. For example, some clinics will not include questions about Indigenous Identity, unless they have an established relationship with relevant local Indigenous groups.

The SPARK tool will be implemented in your clinic for at least the next 6 months. This training is to ensure that you:

- Understand the purpose and importance of collecting this data in healthcare
- Are able to confidently respond to questions from patients about the tool

- Are familiar with the logistics of the implementation

Overview of the SPARK Project: Implementing the Tool in your clinic

- Front desk staff will offer the SPARK Tool to adult patients with appointments
- Staff will give patients a tablet (or a paper form in some clinics) to complete the Tool while they wait for their appointment OR patients will receive an email with a link to the online Tool to complete at home. Some clinics may use different approaches i.e., enlisting the help of volunteers.
- Your clinic is using a software program called OCEAN (or Pomelo in NS) to administer the tool so that it can be completed electronically on the tablet or online.
- OCEAN and Pomelo connect to your clinic’s EMR so:

  i) patients’ tool responses can be directly uploaded to their EMR; and

  ii) automated emails containing a link to the online Tool can be sent to patients with upcoming appointments

Interviews and Focus Groups

- You will be contacted by the research team to share your experience with implementing the Tool at your clinic

- You will be compensated for your time if you choose to participate

CONTINUE

The SPARK Tool Questions

The following section presents the SPARK Tool Questions. The Survey that patients complete will also include "descriptors," which patients can view by clicking a hyperlinked question mark next to each question.

The descriptor (shown on this page beneath each question) contains two sections:

- **"Why are we asking this question?" –** this section explains the purpose of the question and how the information may be used in patient care
- **"Understanding the Question" –** this section clarifies the question and provides definitions of terms

The SPARK Tool Questions

Please note that while responding to the Tool questions is highly encouraged, it is optional.

All responses are anonymous and any data retrieved from these Tool questions is promptly destroyed.

1. Language

1. If available, would you prefer your healthcare appointments offered in another language?

- ◯ Yes
- ◯ No
- ◯ Do not know
- ◯ Prefer not to answer

Why are we asking this question?

This information lets us know who may want an interpreter or translation services at our clinic.

This may also help us provide appropriate care to people who do not speak or read English or French.

2. Born in Canada

1. Were you born in Canada?

- ◯ Yes
- ◯ No
- ◯ Do not know
- ◯ Prefer not to answer

Why are we asking this question?

Providers may be able to refer people to appropriate newcomer services. This information lets us know who we serve at the clinic and may help us identify newcomers to Canada.

3. a) Indigenous Identity

1. Do you identify as an Indigenous person? Select all that apply:

- ☐ Yes, First Nations
- ☐ Yes, Métis
- ☐ Yes, Inuk/Inuit
- ☐ Yes, another Indigenous identity (specify below)
- ☐ [illegible]

Why are we asking this question?

Your provider may use this to refer you to First Nations, Métis or Inuit resources and supports. This information lets us know who we serve and helps us meet patient needs.

First Nations, Métis or Inuit identity data must be collected with local First Nations, Métis, and Inuit governance bodies. This way, the information is appropriately collected, stored, interpreted, used, or shared. This in the line with the First Nations OCAP, Métis OCAS, and Inuit Qaujimajatuqangit data governance and sovereignty principles.[1-4]

This information helps identify and address differences in health outcomes between First Nations, Métis and Inuit people and other racial groups in Canada.[5]

Understanding the question:

We ask “**Do you identify…**” in the question because we are asking you to tell us if you feel you are part of this group. This may include people who are Status or not Status.

Truth and Reconciliation Commission of Canada gives calls to action.[5] These calls to action can improve outcomes for First Nations, Métis, and Inuit peoples in Canada.[5]

Data sovereignty means that each nation governs the collection, ownership, and application of its data. No matter where that data is stored[1-4].

Indigenous: In Canada, Indigenous peoples are First Nations, Métis, and Inuit. These are the original people of the land that is now Canada.[6-9]

First Nations are the original people of the land that is now Canada. First Nations may or may not be Status (registered under the Indian Act) or live in a First Nations community.[10]

Métis have their own history, culture, language, and territory. Métis are people born in the Métis Nation Homeland (Manitoba, Saskatchewan, Alberta, as well as parts of Ontario, British Columbia, and the Northwest Territories).[11]

Inuit are the original people of the Northern region of Canada, Alaska, and Greenland. Their homeland included much of the land, water, and ice in the Artic.[7,12]

References —

1. *Canadian Institute for Health Information. (2020). A path forward: toward respectful governance of First Nations, Inuit and Metis Data Housed at CIHI. Accessed April 14, 2022.*
2. *First Nations Information Governance Centre. The First Nations principles of OCAP®. Accessed April 11, 2022.*

3. *University of Manitoba Faculty of Health Sciences. (2019) Framework for Research Engagement with First Nation, Metis, and Inuit Peoples. Accessed April 11, 2022.*

4. *Tagalik S. (2009–2010) National Collaborating Centre for Aboriginal Health. Inuit Qaujimajatuqangit: The Role of Indigenous Knowledge in Supporting Wellness in Inuit Communities in Nunavut. Accessed April 11, 2022.*

5. *Truth and Reconciliation Commission of Canada. (2015) Truth and Reconciliation Commission of Canada: Calls to Action. Accessed April 11, 2022.*

6. *Statistics Canada. (2021). Indigenous group of person. Accessed April 11, 2022*

7. *The Canadian Encyclopedia. Indigenous Peoples in Canada. Accessed April 11, 2022*

8. *United Nations. Indigenous Peoples at the United Nations. Accessed April 11, 2022*

9. *United Nations. United Nations Declaration on the Rights of Indigenous Peoples. 2018*

10. *Government of Canada. (2014) First Nations People in Canada. Accessed April 11, 2022*

11. *Libraries and Archives Canada. Government of Canada. (2021) First Nations People in Canada. Accessed April 11, 2022*

12. *Government of Canada. (2021) Inuit. Accessed April 11, 2022*

3. b) Indigenous Identity

1. If yes to the previous question, are you Status (Registered or Treaty Indian as defined by the *Indian Act* of Canada)?

- ◯ Yes, Status Indian (Registered or Treaty)
- ◯ No
- ◯ Not applicable, I am not an Indigenous person
- ◯ Do not know

Why are we asking this question?

If you are Status, your provider may be able to refer you to more services.

You may be able to receive Non-Insured Health Benefits. This may include:

- some drugs
- vision care
- medical supplies
- medical transportation

Understanding the question:

Status (Registered or Treaty Indian) is a person registered under the Indian Act of Canada. People with status may receive health services under the Non- Insured Health Benefits Program.[1,2]

References

1. *Government of Canada. (2022). About Indian Status. Accessed April 14, 2022*
2. *Statistics Canada. Registered or Treaty Indian status of person. 2021. Accessed April 11, 2022*

3. c) Indigenous Identity

1. If yes, Inuk/Inuit, are you a member of an Inuit land claims agreement?

- ◯ Yes
- ◯ No
- ◯ Not applicable, I am not an Indigenous person.
- ◯ Do not know
- ◯ [illegible]

Why are we asking this question?

If you are a member of an Inuit land claims agreement your provider may refer you to supports and services that may help you.

You may be able to receive Non-Insured Health Benefits. This may include:

- some drugs
- vision care
- medical supplies
- medical transportation

Understanding the Question

A member of an **Inuit land claims agreement** is a beneficiary of: Nunavut Land Claim Agreement, or Inuvialuit Final Agreement.

You may have been automatically registered and received your territorial healthcare card.

If you live outside of the land claim settlement area you can register with a land claim organization to receive benefits.

References

1. Government of Canada. Your Health Benefits – A Guide for Inuit to Access Non-Insured Health Benefits. Accessed April 22, 2022.

4. Race

1. In our society, people are often described by their race or racial background. Our race may influence the way we are treated by individuals and institutions, and this may affect our health. Which category(ies) best describes you?

   Select all that apply:

- [ ] Black (e.g., African, African Canadian, Afro-Caribbean descent)
- [ ] East Asian (e.g., Chinese, Japanese, Korean, Taiwanese descent)

Why are we asking this question?

We are asking this question on race to help us know who we serve at the clinic. We're interested in monitoring the experience our clients have in the clinic to ensure that everyone has a positive experience regardless of their racial background.

This information may also help us identify and address differences in health outcomes experienced by different racial groups.

Understanding the Question

Race is a term used to classify people into groups usually based on observable physical characteristics (for example, skin colour) but can also include characteristics such as accent or dress.[1,2,3]

Although these differences or groupings are created by the society we live in, racial categories can have significant consequences for people's lives, including unfair and unjust treatment by individuals and institutions. This is what we call racism.

Race should not be confused with ethnicity. Ethnicity refers to groups of people who share a common culture or ancestry. They may also share a common language, religion, geographic origin, nationality, cultural traditions, migration history or other commonalites.[1,2]

Mixed race individuals are encouraged to select more than one racial group.

If you are unsure what to select, you can choose "Another" and specify what racial group(s) best describe you.

This question was adapted from the March 2022 CIHI Race-based standards.[1]

References —

1. *Canadian Institute for Health Information (2022) Guidance on the Use of Standards for Race-Based and Indigenous Identity Data Collection and Health Reporting in Canada Ottawa, ON: CIHI. Accessed April 5, 2022*
2. *Torontohealthequity.ca. Measuring Health Equity: Demographic Data Collection in Health Care. Accessed April 5, 2022*
3. *Government of Ontario Anti-Racism Directorate. Data Standards for the Identification and Monitoring of Systemic Racism. Accessed April 5, 2022*

5. People with Disabilities

1. Do you currently experience any of the following due to a severe and persistent physical or mental condition? Select all that apply:

- [ ] Difficulty seeing (e.g., severe vision impairment)
- [ ] Difficulty hearing (e.g., severe hearing loss)
- [ ] Difficulty with walking or climbing (e.g., severe mobility issues)
- [ ] Difficulty remembering or with concentration (e.g., severe memory loss or disorientation)

Why are we asking this question?

Your provider may use this to inform the care you receive.

Your provider may also use this to refer you to appropriate services that may be helpful to you.

This information may help us provide better accommodations for people living with disabilities. And this can improve their overall access to healthcare.

Understanding the Question

"Severe and persistent condition" is about long-term conditions or disabilities that affect day-to-day activities.

The condition **does not have to be diagnosed** by your provider.

'Difficulty seeing or hearing' is not asking about conditions that can be corrected with aids such as glasses or hearing aids.

'Difficulty with communication' is asking about people who have this difficulty because of a physical or mental condition and not because of other things such as language barriers or reading or writing level.

6. Sex at Birth

1. What was your sex at birth?

- ○ Female
- ○ Male
- ○ Intersex
- ○ Do not know
- ○ [illegible]

Why are we asking this question?

This information lets us know who we serve at the clinic and how well we are meeting patient needs. This information can also help us screen for and prevent some health

conditions.

This question gives you a space to share your identity and to help avoid assumptions about you or your healthcare.

Understanding the Question

Sex at Birth is often recorded at a person's birth and is usually based on a person's reproductive system and other physical traits.[1]

While **Gender Identity** is a person's sense of themselves relating to gender, be it male, female, a combination of both, or neither.[2]

Gender can be fluid and may change over time.[3]

Intersex refers to people born with differences in their reproductive features[2,4] that may not be easily characterized as male or female.[5]

References —

1. *Statistics Canada, Government of Canada (2021).Sex at birth of person Accessed April 18, 2022*
2. *Human Rights Campaign (2011). Glossary of Terms - Human Rights Campaign. Accessed April 18, 2022*
3. *Torontohealthequity.ca. Measuring Health Equity: Demographic Data Collection in Health Care. Accessed April 18, 2022*
4. *Ucdavis.edu. (2014). LGBTQIA Resource Center Glossary | LGBTQIA Resource Center. Accessed April 18, 2022*
5. *Ohrc.on.ca. (2014). Appendix B: Glossary for understanding gender identity and expression | Ontario Human Rights Commission (ohrc.on.ca). Accessed April 18, 2022*

7. Gender Identity

1. What is your gender identity?

- ◯ Woman
- ◯ Man
- ◯ Transgender man
- ◯ Transgender woman

Why are we asking this question?

This information lets us know who we serve at the clinic and how well we're meeting patient needs.

It helps us understand the range of gender identities that people at our clinic may experience.

This question gives you a space to share your identity and to help avoid assumptions about you or your healthcare.

Understanding the Question

Gender Identity is a person's sense of themselves relating to gender, be it male, female, a combination of both, or neither.[1]

Gender can be fluid and may change over time.[2]

Sex at Birth is often recorded at a person's birth and is usually based on a person's reproductive system and other physical traits.[3]

Gender non-binary describes a person who may experience a gender identity that is neither exclusively woman or man, is a combination of woman and man or is between or beyond genders.[4]

Non-binary can also be used as an umbrella term for identities that are gender fluid, moving between and through identities.[1]

Two-Spirit is a term used by Indigenous peoples to self-describe and express diverse sexual, gender and/or spiritual identities.[5] It is a way for Indigenous peoples to reconnect with their traditional languages, ways, and cultures.[6]

Transgender or trans is an umbrella term for people whose gender identity or expression is different from conventional or cultural expectations based on the sex they were assigned at birth.[7,8]

References —

1. *Ohrc.on.ca. (2014). Appendix B: Glossary for understanding gender identity and expression | Ontario Human Rights Commission. Accessed April 18, 2022*

2. *Torontohealthequity.ca. Measuring Health Equity: Demographic Data Collection in Health Care. Accessed April 18, 2022*
3. *Statistics Canada, Government of Canada (2021). Sex at birth of person. Accessed April 18, 2022*
4. *Losty, M. and O'Connor, J. (2017). Falling outside of the 'nice little binary box': a psychoanalytic exploration of the non-binary gender identity. Psychoanalytic Psychotherapy, 32(1), pp.40–60*
5. *Knudson, S. and Hahn, D. (2019). Committing sociology: critical perspectives on our social world. Toronto: Pearson Canada.*
6. *Fewster, P.H. (2018). Researching for LGBTQ Health. Lgbtqhealth.ca. Accessed April 18, 2022*
7. *Ucdavis.edu. (2014). LGBTQIA Resource Center Glossary | LGBTQIA Resource Center. Accessed April 18, 2022*
8. *Human Rights Campaign (2011). Glossary of Terms - Human Rights Campaign. Accessed April 18, 2022*

8. Sexual Orientation

1. Which category(ies) best describe your sexual orientation? Select all that apply:

- [ ] Asexual or Aromantic
- [ ] Bisexual
- [ ] Demisexual
- [ ] Homosexual or Gay

Why are we asking this question?

This information lets us know who we serve at the clinic. It helps us understand the range of orientations that people at our clinic may experience.

This question gives you a space to share your orientation and to help avoid assumptions about you or your healthcare.

Understanding the Question

Sexual orientation is a term for the emotional, physical, romantic, sexual, and spiritual attraction, non-attraction, desire, or affection for another person.[1]

Aromantic describes a romantic identity for people who do not experience romantic attraction to people of any gender but may still have sexual attraction to other people.[2]

Asexual describes people who do not experience sexual attraction to people of any gender but may still experience romantic attractions to other people.[1,3]

Bisexual describes a person whose primary emotional, romantic, or sexual attraction is toward people of both the same and different gender.

Demisexual describes a person who feels sexual attraction only to people that they have an emotional bond with.[3]

Homosexual or Gay describes a person whose primary emotional, romantic, or sexual attraction is toward people of the same gender.[1]

Heterosexual or Straight describes a person whose primary emotional, romantic, or sexual attraction is toward people of an opposite gender.

Lesbian describes a person, usually a woman, whose primary emotional, romantic, or sexual attraction is toward people of the same gender.[1]

Pansexual describes a person who has emotional, romantic, or sexual desire for people of all genders and sexes.[1]

Queer is a term used to express a range of identities and orientations that are beyond the mainstream.[1]

Two-Spirit is a term used by Indigenous peoples to self-describe and express diverse sexual identities as having both a masculine and feminine spirit.[4] It is a way for Indigenous peoples to reconnect with their traditional languages, ways, and cultures.[5]

References

1. *Human Rights Campaign (2011). Glossary of Terms - Human Rights Campaign. Accessed April 18, 2022*
2. *Suen, L.W., Lunn, M.R., Katuzny, K., ... & Obedin-Maliver, J. (2020). What Sexual and Gender Minority People Want Researchers to Know About Sexual Orientation and Gender Identity Questions: A Qualitative Study. Archives of Sexual Behavior, 49(7), pp.2301–2318.*
3. *Ucdavis.edu. (2014). LGBTQIA Resource Center Glossary | LGBTQIA Resource Center. Accessed April 18, 2022*
4. *Knudson, S. and Hahn, D. (2019).* Committing sociology: critical perspectives on our social world. *Toronto: Pearson Canada.*
5. *Fewster, P.H. (2018). Researching for LGBTQ Health. Lgbtqhealth.ca. Accessed April 18, 2022*

9. Education

1. What is your current level of education?

- ◯ No formal schooling
- ◯ Grade school (grade 1-8)
- ◯ Some high school, but did not graduate
- ◯ High school or high school equivalency certificate (grade 9- 12)
- [illegible]

Why are we asking this question?

Your provider may use this information to know how well you are able to read and understand health information such as medication labels and instructions.[1]

Your provider may also use this to connect you with supports that may be helpful to you.

A person's education level is related to their ability to understand basic health information and access health services. And these may affect their health.[1]

Understanding the Question

The term ***'some'*** in ***'Some high school'*** means your high school education may be in progress or stopped.

The term 'ongoing' means schooling or training is in progress.

If you received your education by home-schooling, please select the matching grade level you have completed.

If your educational experiences fall outside the scope of the categories described above (e.g., cultural or spiritual education within your community), please select the most equivalent grade level.

References —

1. *Canadian Institute for Health Information (2018) In Pursuit of Health Equity: Defining Stratifies for Measuring Health Inequality— A Focus on Age, Sex, Gender, Income, Education and Geographic Location. Ottawa, ON: CIHI. Accessed April 4, 2022*

10. Income/Finances

1. Do you currently have difficulty paying for basic needs?

- ◯ Yes
- ◯ No
- ◯ Not applicable, I do not have to pay for basic needs
- ◯ Do not know

Why are we asking this question?

Your provider may use this to refer you to income resources and supports that may be helpful to you.

Low income has been shown to negatively affect mental and physical health.[1-4]

Understanding the Question

Basic Needs are things that a person requires to achieve and maintain physical and mental well-being and include food, water, shelter, and clothing.[2-4]

References —

1. *National Association of Community Health Centre, Inc, Association of Asian Pacific Community Health Organizations, and the Oregon primary Care Association. (2019). PRAPARE. Protocol for Responding to and Assessing Patients' Assets, Risks and Experiences. Implementation and Action Toolkit. Accessed April 14, 2022.*
2. *Collin, C. and Campbell, B. (2008). Measuring Poverty: A Challenge for Canada. Accessed April 14, 2022*
3. *Sarlo C. (2013). Poverty: Where do we draw the line? Accessed April 13, 2022*
4. *Homeless Hub. (2021). Basic Needs. Accessed April 13, 2022*

11. Food Security

Please respond to the following statements:

1. "Within the past 12 months, we worried whether our food would run out before we could buy or get more"

- ○ Often true
- ○ Sometimes true
- ○ Never true

Why are we asking this question?

Your provider may use this to refer you to resources and supports that may help you obtain food and maintain a healthy diet.[1,2]

Your provider may also use this to give you advice on regular eating and nutrition.[1]

Not having enough food can mean choosing less expensive, more filling, and less healthy food. This can negatively affect your health.[2]

This question was adapted from the Hunger Vital Sign™ screening tool.[3]

References —

1. *National Association of Community Health Centre, Inc, Association of Asian Pacific Community Health Organizations, and the Oregon primary Care Association. (2019). PRAPARE. Protocol for Responding to and Assessing Patients' Assets, Risks and Experiences. Implementation and Action Toolkit. Accessed April 14, 2022*

2. *Botelho, FC., Junior, IF., Guerra, LD., Rodrigues, SF., Tonacio, LV. (2020). Scientific literature on food and nutrition security in primary health care: A scoping review. Global Public Health, 15(12), pp. 1902-1916.*

3. *Hager, E.R., Quigg, A.M., Black, M.M., ... & Cook, J.T., de Cuba, S.A.E., Casey, P.H., Chilton, M. and Cutts, D.B., 2010. Development and validity of a 2-item screen to identify families at risk for food insecurity. Pediatrics, 126(1), pp.e26-e32.*

12. Medication Access

1. In the past 12 months, were you unable to get medicine or medical supplies, or did you do anything to make them last longer *because of the cost*?

- ◯ Yes
- ◯ No
- ◯ Not applicable, I did not have to get any medicine or medical supplies in the past 12 months

Why are we asking this question?

Your provider may use this to refer you to resources that can help you access medicine or medical supplies.

This may also help your provider consider lower cost or free medicine options.

Being able to access and use needed medicine and medical supplies can affect your health.[1]

Understanding the Question

'Medicine' includes both prescription and over-the-counter medicines.

'Medical supplies' include any equipment you may need to manage your health or disability. For example, needles, glucose monitors, walking aids.

References —

1. *Sobeski, L. M., Schumacher, C. A., Alvarez, N. A., ... & Van Dril,E. (2021). Medication access: Policy and practice opportunities for pharmacists. Journal of the American College of Clinical Pharmacy, 4(1), 113-125.*

13. a) Housing

1. What is your current housing situation?

◯ A place you or your family owns

◯ A place you or your family rents

◯ Social housing, Subsidized housing or Rent-geared-to-income

◯ Supportive housing or Group Home

◯ [illegible]

Why are we asking this question?

Your provider may use this to refer you to resources and supports that may help you with housing needs.

It is known that unstable housing can negatively affect people's health.[1,2]

Understanding the Question

Social housing is housing that is subsidized or partly paid for by a level of government.[3]

Subsidized housing means receiving help paying for housing from the government or a private organization.[4]

Rent-geared-to-income is a type of rent assistance. It lets a person pay a percentage of their income for rent.[5]

Supportive housing refers to housing with on-site staff who provide residents with ongoing support with their needs.

References —

1. *Krieger, J. and Higgins, D.L. (2002). Housing and Health: Time Again for Public Health Action. American Journal of Public Health, 92(5), pp.758–768.*
2. *Office of Disease Prevention and Health Promotion (2014). Housing Instability | Healthy People 2020. Healthypeople.gov. Accessed April 7, 2022*
3. *www.homelesshub.ca. Affordable Housing | The Homeless Hub. Accessed April 7, 2022*
4. *Settlement.org. (2018). What is subsidized housing? Accessed April 7, 2022*

5. *www.durham.ca. (2021). Rent-Geared-to-Income Housing (RGI) - Region of Durham . Accessed April 7, 2022*

13. b) Housing

1. Who do you live with? Select all that apply:

- [ ] Parent(s) or Guardian(s)
- [ ] Spouse or Partner
- [ ] Child(ren)
- [ ] Grandparent(s)
- [ ] [illegible]

Why are we asking this question?

This information may help your provider further understand your living situation and the social supports that may be available to you.

This may also help provide information on aspects of your overall health.

It is known that living with others can impact your health.[1,2]

References —

1. *Krieger, J. and Higgins, D.L. (2002). [Housing and Health: Time Again for Public Health Action.](#) American Journal of Public Health, 92(5), pp.758–768.*

2. *Office of Disease Prevention and Health Promotion (2014). [Housing Instability | Healthy People 2020.](#) Healthypeople.gov. Accessed April 7, 2022*

13. c) Housing

1. In the past 12 months, was there a time when you were not able to pay the mortgage or rent on time?

○ Yes

○ No

○ Not applicable, I do not have to pay rent or mortgage

○ Do not know

Why are we asking this question?

Your provider may use this to refer you to housing or financial resources and supports that may be helpful to you.

Not being able to pay your mortgage or rent on time may negatively affect your health (for example, it may cause stress).[1,2]

References

- *Krieger, J. and Higgins, D.L. (2002). Housing and Health: Time Again for Public Health Action. American Journal of Public Health, 92(5), pp.758–768.*
- *Office of Disease Prevention and Health Promotion (2014). Housing Instability | Healthy People 2020. Healthypeople.gov. Accessed April 7, 2022*

14. Transportation

1. In the past 12 months, has lack of transportation kept you from medical appointments, meetings, work, or from getting things needed for daily living?
   Select all that apply:

- ◯ Yes, it has kept me from medical appointments or getting medicines
- ◯ Yes, it has kept me from non- medical meetings, appointments, work, or getting things that I need

Why are we asking this question?

Your provider may use this to refer you to transportation resources and supports.

Lack of transportation can impact your health.[1-4] It may make it hard to get to a healthcare appointment on time. It can also make it hard to get to and from work, get healthy food, use medicine on time, or get something you need.

Understanding the Question

"Medical Appointments" are appointments to meet your health needs. This may include an appointment with your doctor(s), specialist, or social worker. This also includes getting medicine from your doctor or pharmacist.[1]

You might lack transportation because of:[4]

- cost and access,
- distance,
- gas or parking costs,
- public transportation safety,
- and/or other reasons.

"Things needed for daily living" are things such as groceries or personal products that you need to be healthy.

This question was adapted from the PREPARE tool.[1]

References —

1. *National Association of Community Health Centre, Inc, Association of Asian Pacific Community Health Organizations, and the Oregon primary Care Association. (2019). PRAPARE. Protocol for Responding to and Assessing Patients' Assets, Risks and Experiences. Implementation and Action Toolkit. Accessed April 14, 2022*

2. *Billioux, A., Verlander, K., Anthony, S., Alley, D. Standardized screening for health-related social needs in clinical settings: the accountable health communities screening tool. Discussion Paper, National Academy of Medicine, Washington, DC. Accessed April 14, 2022*

3. *Razon, N., Gottlieb, L. (2022). Content Analysis of Transportation Screening Questions in Social Risk Assessment Tools: Are we capturing transportation insecurity. Journal of the American Board of Family Medicine, 35(2), pp.400-405.*

4. *Syed, ST., Gerber, BS., Shapr, LK. (2013). Traveling toward disease" transportation barriers to health care access. J community Health, 38(5), pp. 976-993.*

15. Phone and Internet Access

1. Do you currently have consistent access to a phone or the internet?

- ◯ Yes, phone only
- ◯ Yes, internet only
- ◯ Yes, both
- ◯ No
- ◯ Do not know

Why are we asking this question?

Your provider may use this to refer you to resources that can improve your access to phone and internet services.

These resources may help improve your access to healthcare appointments and health information.

This information may also inform how you receive healthcare services. For example, by phone, video, or in-person.

Access to a phone and internet is now a basic human right. This is due to its impact on access to healthcare and other needed services.[1,2]

Understanding the Question

"Consistent access" means having regular, reliable, and adequate access to a phone or internet to obtain needed services. This includes access to a phone or internet for a healthcare appointment.[3] This also includes access to health information that is on the internet that your provider refers you to.[3]

References —

1. *Benda, NC., Veinot, TC., Sieck, C., Ancker, JS. (2020). Broadband internet access is a social determinant of Health! AM J Public Health, 110(8), pp. 1123-1125. doi:*
2. *Rubin, R., 2021. Internet access as a social determinant of health. JAMA, 326(4), pp.298-298.*
3. *Graves, JM., Abshire, DA., Amiri, S., Mackelprang, JL. (2021). Disparities in technology and broadband internet access across rurality: implications for health and education. Fam Community Health, 44(4), pp. 257-265.*

16. Utilities

1. In the past 12 months, did you miss making a payment on any utility bills (e.g., electric, gas/oil, water) *because of cost*?

◯ Yes

◯ No

◯ Not applicable, I did not have to pay utility bills in the past 12 months or utilities already included in rent

◯ Do not know

Why are we asking this question?

Your provider may use this to refer you to supports that may be helpful to you.

Difficulty paying for utilities can negatively affect your health.[1] For example, you need heat when it is cold outside.[2]

Difficulty paying utility bills can also be a sign of financial need, which can affect your health (for example, it may cause stress).

Understanding the Question

This question is asking about basic utilities. Basic utilities may include:

- heating or cooling
- electricity or hydro
- water or sewer
- phone, and internet.

The specific utilities you pay for may depend on where you live. For example, you may heat your home using natural gas, furnace oil or wood depending on where you live.

References —

1. *International Policy Centre for Inclusive Growth (2009) Equitable access to basic utilities: Public versus private provision and beyond. Accessed: April 20, 2022.*
2. *Grey, C. N., Jiang, S., Nascimento, C., Rodgers, S. E., Johnson, R., Lyons, R. A., & Poortinga, W. (2017). The short-term health and psychosocial impacts of domestic energy efficiency investments in low-income areas: a controlled before and after study. BMC Public Health, 17(1), 1-10.*

17. Social Supports

1. Do you feel you have people who you can open up to or confide in?

- ◯ Yes, I always or sometimes have someone
- ◯ No, I don't have anyone
- ◯ Do not know
- ◯ Prefer not to answer

Why are we asking this question?

Question 1: Your provider may use this to refer you to social resources and social supports that may be helpful to you.

Question 2: Your provider may refer you to social resources and social supports that may be helpful to you.

Having "people you can rely on" can help you to feel more socially connected and secure, and this may impact your health.

Understanding the Question

Having **"people you feel you can open up to or confide in"** means being able to talk to people that you care about, trust and feel close to.[1]

Having social relationships and feeling connected can impact health.[1]

References —

1. *National Association of Community Health Centre, Inc, Association of Asian Pacific Community Health Organizations, and the Oregon primary Care Association. (2019). PRAPARE. Protocol for Responding to and Assessing Patients' Assets, Risks and Experiences. Implementation and Action Toolkit. Accessed April 14, 2022*

18. a) Employment

1. Are you currently employed (this includes self-employed, full-time, part-time or other)?

- ◯ Yes
- ◯ No
- ◯ Do not know
- ◯ Prefer not to answer

Why are we asking this question?

Your provider may use this information to better understand employment circumstances that could be affecting your health. Your provider may also refer you to employment resources that could be helpful. It is known that those who work tend to have better health than those who are unemployed.[1]

References

1. *Waddell, G., & Burton, A. K. (2006). Is work good for your health and well-being? Accessed April 4, 2022.*

18. b) Employment

1. Are you currently looking for work?

- ○ Yes
- ○ No
- ○ Not applicable, I am currently employed
- ○ Do not know

Why are we asking this question?

Your provider may use this information to refer you to employment resources that may be helpful.

18. c) Employment

1. Is your main job temporary or part-time (e.g., casual, contract, freelance, short-term, seasonal)?

- ◯ Yes
- ◯ No
- ◯ Not applicable, I am currently employed
- ◯ Do not know

Why are we asking this question?

Your provider may use this information to better understand employment circumstances that may be affecting your health.

Your provider may also refer you to employment resources that could be helpful.

It is known that unstable or unpredictable work negatively affects peoples' health.[1,2]

Understanding the Question

“Main job”: If you have more than one job, your main job is your primary source of income.

References —

1. *Benach, J., Vives, A., Amable, M., Vanroelen, C., Tarafa, G. and Muntaner, C., 2014. Precarious employment: understanding an emerging social determinant of health. Annual review of public health, 35, pp.229-253.*
2. *Benach, J., Vives, A., Tarafa, G., Delclos, C. and Muntaner, C., 2016. What should we know about precarious employment and health in 2025? Framing the agenda for the next decade of research. International journal of epidemiology, 45(1), pp.232- 238.*

18. d) Employment

1. Do you feel that your current employment could be negatively affected if you raised concerns about your work (e.g., health, safety, rights)?

◯ Yes

◯ No

◯ Not applicable, I am currently employed

◯ Do not know

Why are we asking this question?

Your provider may use this information to better understand employment circumstances that may be affecting your health.

Your provider may also refer you to employment resources that could be helpful.

A person's inability or unwillingness to raise concerns about health, safety and rights at work can negatively affect their health.[1]

Understanding the Question

Health and safety concerns at work may include concerns about unsafe work (for example, lifting items that are too heavy, working with violent clients), unsafe work environments (for example, slippery floors, broken machinery), or harassment due to race, gender, or sexual orientation.[1]

Concerns about employment rights may include questions about your right to breaks, sick leave or vacation time.[1]

Your health and safety rights1 include your right to:

- know about unsafe work
- refuse unsafe work
- participate in workplace health and safety decisions

References —

1. *Lewchuk, W., 2013. The limits of voice: Are workers afraid to express their health and safety rights. Osgoode Hall LJ, 50(4), p. 789-812. Accessed April 20, 2022*

18. e) Employment

1. In the past 12 months, did your income change a lot from month to month?

- ◯ Yes
- ◯ No
- ◯ Not applicable, I am currently employed
- ◯ Do not know

Why are we asking this question?

Your provider may use this information to understand employment conditions that could be affecting your health.

Your provider may also refer you to employment or income resources that could be helpful.

Changing income is a key feature of unstable or unpredictable work. This is known to have negative effects on health[1].

References —

1. *Lewchuk, W., 2013. The limits of voice: Are workers afraid to express their health and safety rights. Osgoode Hall LJ, 50(4), p. 789-812. Accessed April 20, 2022*

Optional Question: Ethnicity

1. What is your ethnic or cultural background?
   e.g., Chinese, Filipino, Guyanese, Scottish, Somali, Korean

   Enter your answer

Never give out your password. Report abuse

Microsoft 365

Why are we asking this question?

This question gives people a space to share their ethnic or cultural identity. This information lets us know who we serve at the clinic and may help us understand and address the unique needs of our patients.

Understanding the Question

This question on ethnicity should not be confused with Race. Ethnicity refers to groups of people who share a common culture or ancestry. They may also share a common language, religion, geographic origin, nationality, cultural traditions, migration history and so on.[1,2]

Race is a term used to classify people into groups usually based on observable physical characteristics (e.g., skin colour) but can also include characteristics such as accent, dress and so on.[1,2,3]

Although these differences or groupings are created by the society we live in, racial categories can have significant consequences for people's lives, including unfair and unjust treatment by individuals and institutions. This is what we call racism.

We welcome you to share the ethnic group or culture you identify with.

References —

1. *Canadian Institute for Health Information (2022) Guidance on the Use of Standards for Race-Based and Indigenous Identity Data Collection and Health Reporting in Canada Ottawa, ON: CIHI. Accessed April 5, 2022*

2. *Torontohealthequity.ca. Measuring Health Equity: Demographic Data Collection in Health Care. Accessed April 5, 2022*

3. *Government of Ontario Anti-Racism Directorate. Data Standards for the Identification and Monitoring of Systemic Racism. Accessed April 5, 2022*

Optional Question: Religion

1. What is your religious or spiritual affiliation? Select all that apply:

- ☐ Agnosticism
- ☐ Animism or Shamanism
- ☐ Atheism
- ☐ Baha'i Faith
- ☐ [illegible]

Why are we asking this question?

Your provider may use this to better understand how your religious or spiritual beliefs and practices influence your health or health behaviours. This may include your diet, holidays you observe, or the healthcare treatment options you feel comfortable with.

Your provider may use this to inform the care you receive.

It is known that religious affiliation or involvement can positively affect health[1].

Understanding the Question

“Religious or spiritual affiliation” is about your connection with a particular set of beliefs, attitudes, or practices. This may or may not be related to a specific religion (for example, Catholicism, Islam) or belief in a higher being.[1] This also relates to a person's sense of spirituality.

Select the religion(s) you feel connected to. This includes a religion you are actively practising, your family raised you in, or you feel close to.

You can select more than one option. If you do not find your religion listed, you can write it in.

References —

1. *Chatters, L. (2000) ‘Religion and Health: Public health research and practice,’ Annual Review of Public Health, 21, pp. 335-367.*

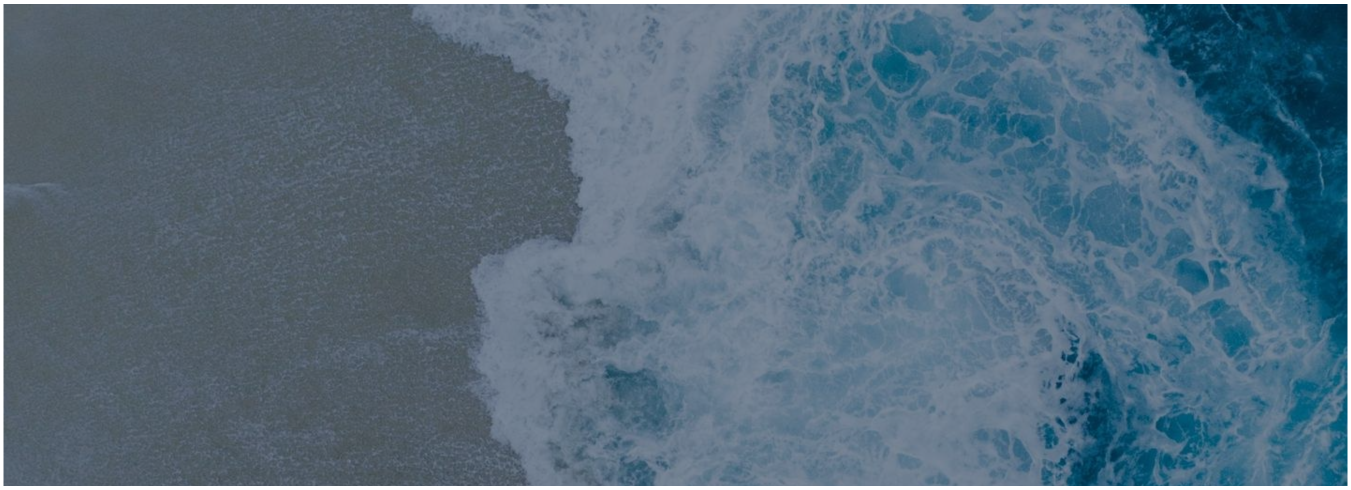

How can patient sociodemographic data be used in healthcare?

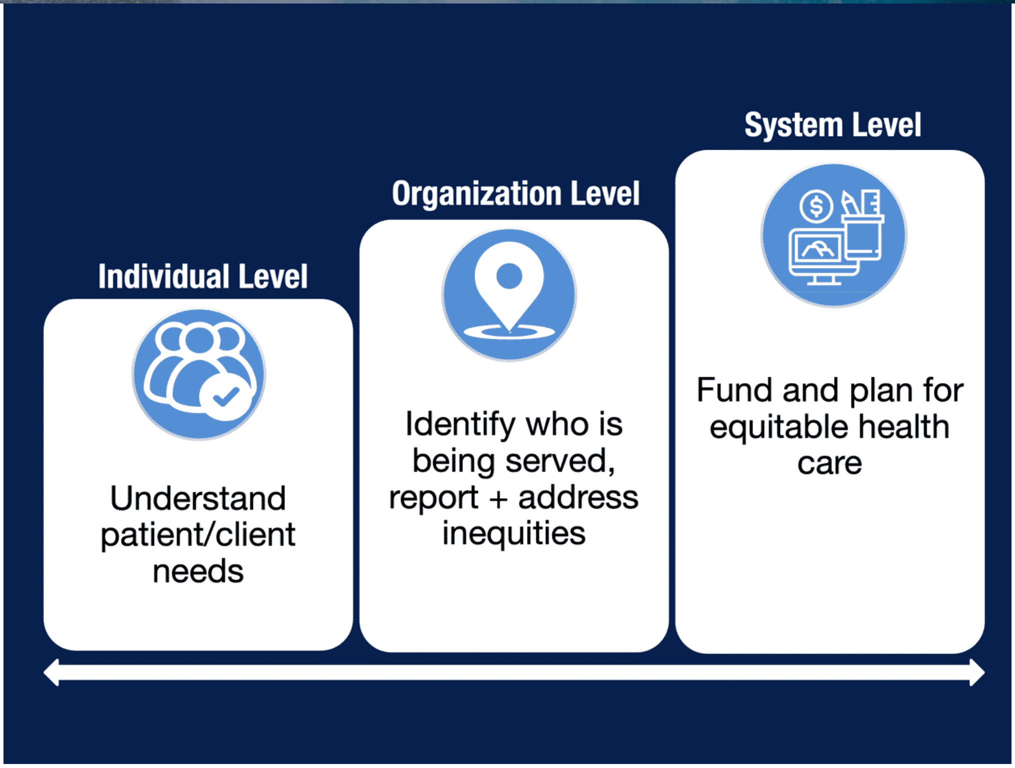

CONTINUE

Social Needs Resources for Patients and Providers

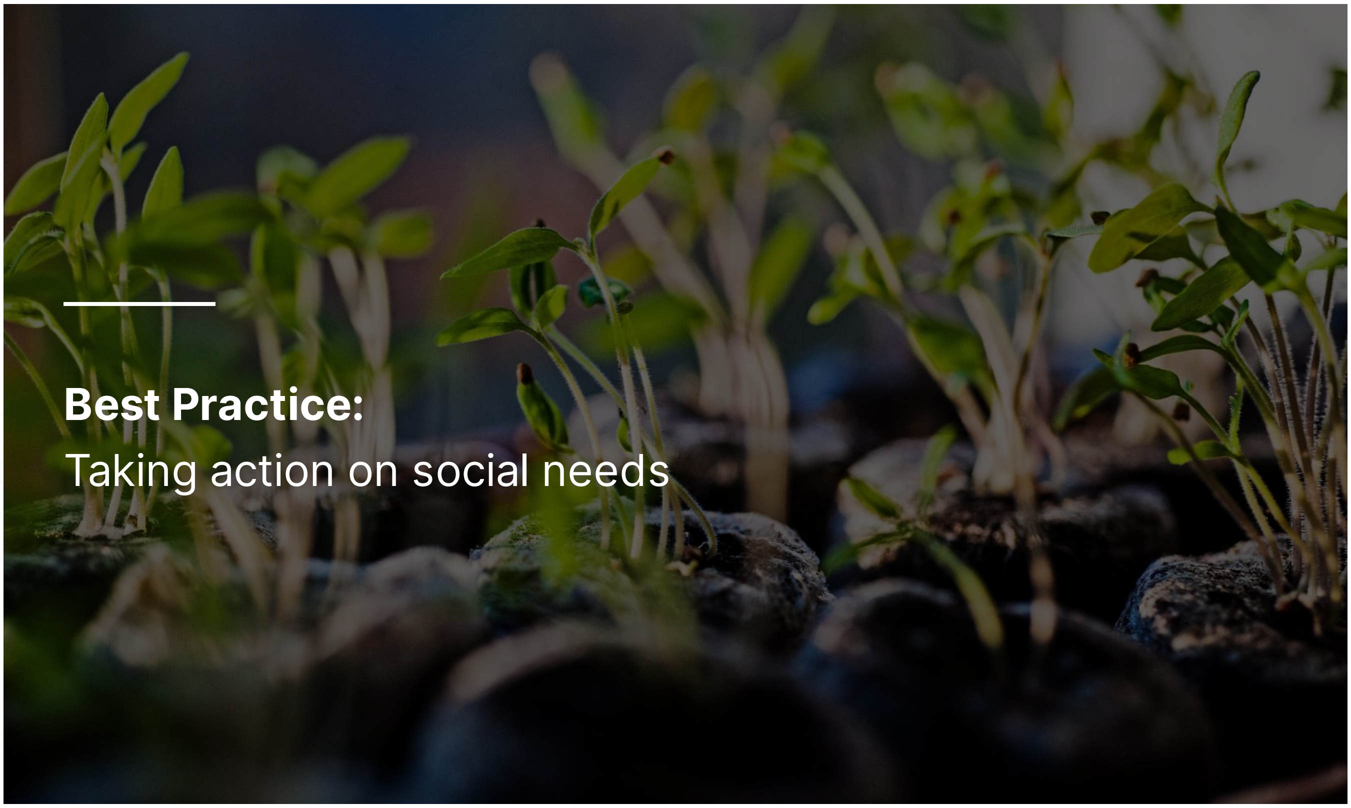

- All patients will be provided a list of local resources after they complete the Tool
- Providers will be given a list of local resources, to help address any needs identified by patients in the SPARK Tool

- Resources provided will relate to each of the domains in the Tool e.g., newcomer supports, food banks, housing supports, and so on.
- In addition, Providers are encouraged to go over the resource list with their patients, and to refer them to resources that would be helpful to address their identified social need

Role of Front Desk Staff

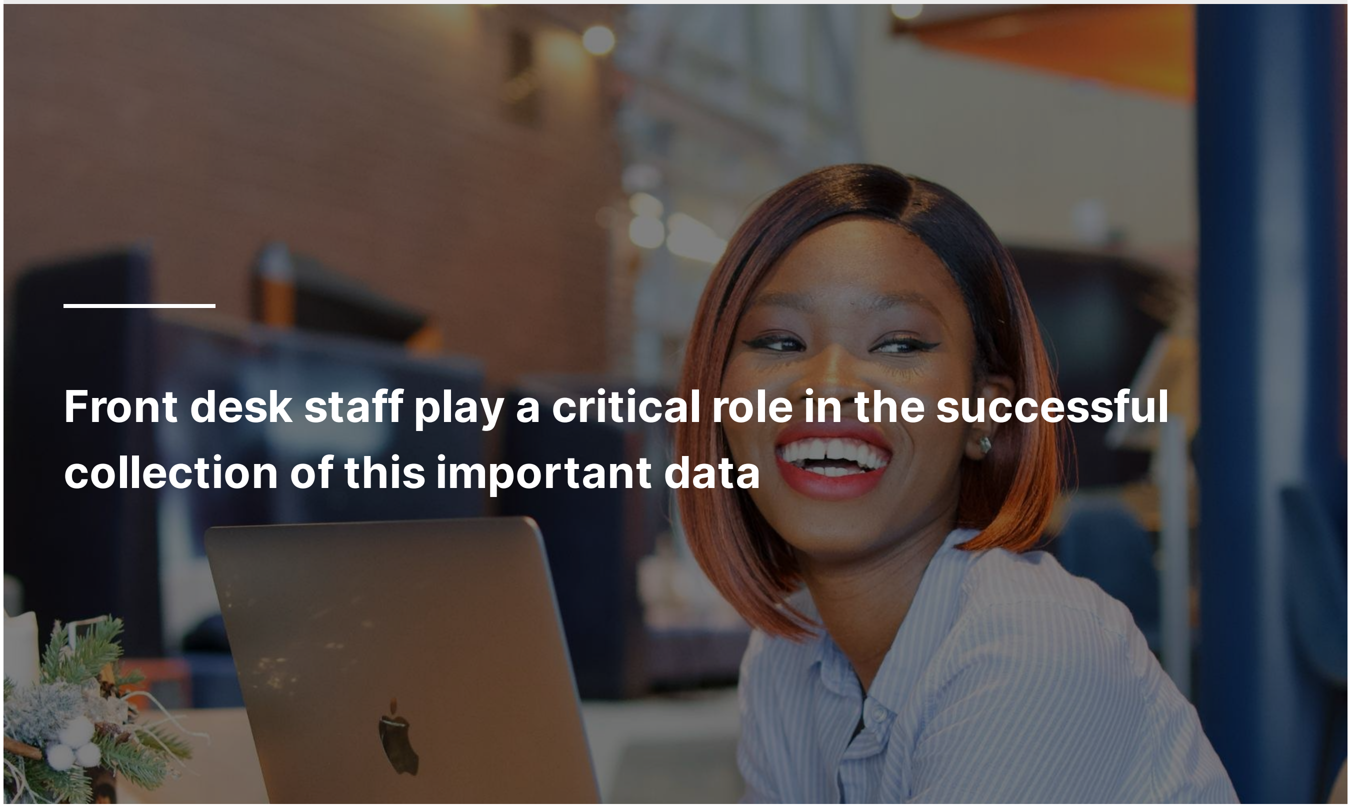

In a previous study, 42,396 patients visited a Toronto-based family health team within a 3 year period (2013-2016):

- Only 35% of patients were offered the survey by the front desk staff, 65% were not offered the survey.
- The majority (90%) of patients who were offered the survey completed it.

Most patients will complete the Tool, but we need front desk staff to offer it to them!

CONTINUE

Introducing the SPARK Tool to Patients

3 key messages to convey when offering the Tool to patients

1) Why are we asking?

We are collecting social information from patients to:

- find out who we serve and what unique needs our patients have

- understand patient experiences and outcomes
- improve delivery of care

2) The Tool is voluntary.

This Tool is voluntary and patients can decline to complete it.

Not completing the Tool will have no negative affect on patients' care.

Each question is voluntary and patients can choose 'prefer not to answer' to any or all questions.

3) Who will see the information?

This information is confidential

Patient responses on the SPARK Tool will only be visible to their direct healthcare team

For research purposes, Tool data will be combined with data from all other patients, with all identifying information removed. No one will be able to identify an individual patient.

Sample Messages

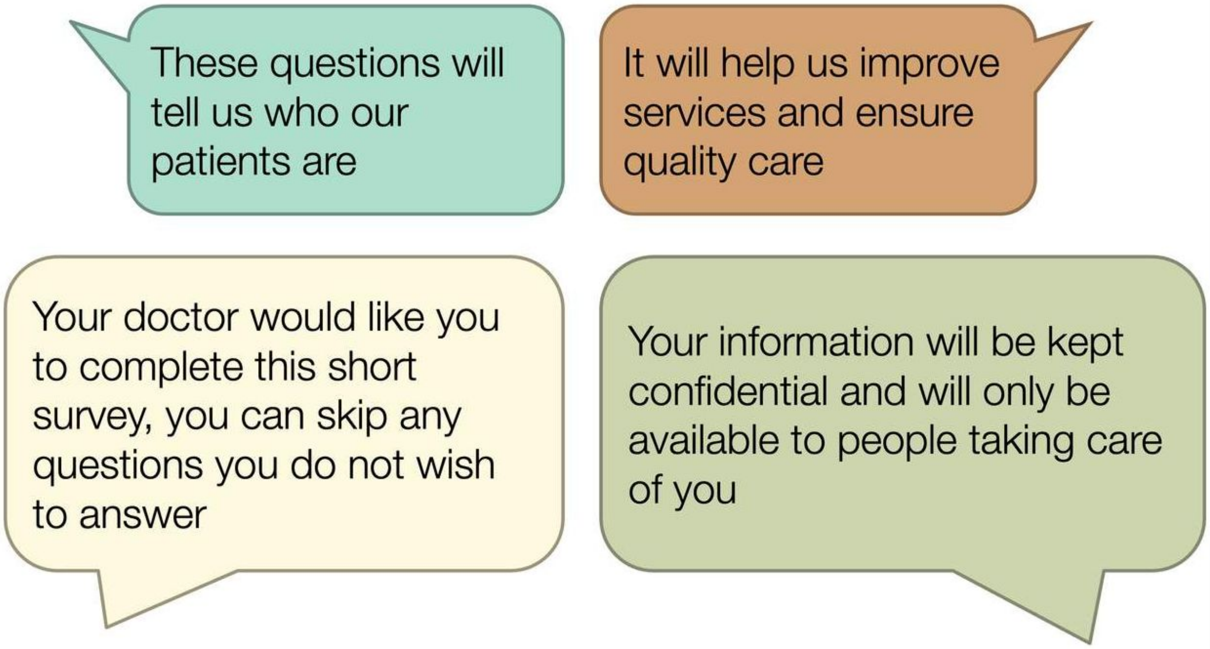

CONTINUE

Role Playing - How Would You Respond?

Consider the following scenarios that you may encounter as you offer the SPARK Tool to patients, and practice some ways you can respond

Scenario 1: Front Desk Check-in

Today was your first day offering the SPARK Tool to patients.

Patients have been receptive so far and no one has refused to answer the questions. They have hardly asked you anything about the Tool and no one has complained. You are no longer anxious about asking patients to complete the Tool.

You greet the next patient and their partner. The patient is unwell and unable to focus and complete any of the questions. The patient asks their partner to complete the Tool on their behalf. The partner takes the Tool, looks at it and looks curious. The patient's partner proceeds to ask questions about the Tool.

QUESTION	POTENTIAL RESPONSES

Question: Why do they have to answer all these questions before they see the doctor?

QUESTION	POTENTIAL RESPONSES

Things you can say:

- "Completing this survey is voluntary. You can choose not to complete it, or you can select 'prefer not to answer' for any individual questions that you don't want to answer"
- "We are collecting this information because we want to provide care based on our patient needs. We don't want to make any assumptions about patient needs or who our patients are. For example, when we know a patient doesn't speak English, we can look into interpreter services."
- "In some cases, depending on the patient and the situation, knowing this information means providing better care."

QUESTION	POTENTIAL RESPONSES

Question: What is the purpose of knowing the patient's gender, sexual orientation, ability to pay for basic needs/utilities?

QUESTION	POTENTIAL RESPONSES

Things you can say:

- “We welcome all patients at our clinic. We don’t want to make assumptions, so we ask everyone.”
- “We may use this information about your gender identity to determine screening or treatment options”
- “Information about your sexual orientation can help us to deliver health services that best suit your needs”
- “It’s helpful for us to know about your ability to pay for the things you need because we want to understand the connection between any financial needs you may have and your health, and we want to help where we can.
- “Only members of your healthcare team will see your survey responses. The information you provide is protected by law like all of your other health information.”

Scenario 2: Front Desk Check-in

You are the front desk staff and distribute and explain the Tool to patients as they arrive.

When you hand the Tool to the next patient, you notice that she starts to look uncomfortable, and seems to look irritated and upset as she reads through the questions.

TASK	POTENTIAL RESPONSES

Task: Explain why the information is being collected

TASK	POTENTIAL RESPONSES

Things you can say:

- "We are collecting information from patients to find out who we serve at this clinic and to understand the unique needs our patients may have. We don't want to guess or make assumptions, so we are asking you to tell us about yourself."
- "This information will help your healthcare provider understand your health outcomes as well as any experiences or needs that may affect your health"
- "Knowing more about you will allow us to improve the care we provide"

TASK	POTENTIAL RESPONSES

Task: Provide information about the questions on education, housing and employment

TASK	POTENTIAL RESPONSES

Things you can say:

- "A patient's ability to understand basic health information and access services may be linked to their education level. This information will help your healthcare provider to know how well you are able to understand and read health information such as medication labels and instructions. They may be able to provide support where needed."
- "Unstable housing and/or living alone can affect your health. Knowing where you live and who you live with can help your healthcare provider understand your health and they may be able to identify supports that can help you."
- "Being unemployed, working in an unstable environment, or having unpredictable income can affect your health. Knowing your work situation can help your healthcare provider understand your health and identify supports that may help."

CONTINUE

Implementation Resources and Materials

Staff Resources you will receive

Cheat Sheet

- Cheat sheet/workstation poster with the 3 key messages for introducing the SPARK tool
- Instructions on what to say when providing the tablet to patients and collecting it from them

Three Key Messages

For Introducing the SPARK Tool to Patients

Why

- Find out who we serve
- Identify patient needs
- Understand patient experiences and outcomes

Voluntary

- The questions are voluntary
- You can choose 'prefer not to answer' to any/all questions
- Your responses will not affect your care

Confidential

- Responses only visible to healthcare team
- Protected like all health information
- If used in research, responses will be grouped with other data
- Researchers will not be able to identify you

Definitions of Key Terms

- Provides plain language definitions of key terms used on the SPARK Survey

Definitions of Key Terms in SPARK Tool

Socio-demographics

Indigenous Identity

Truth and Reconciliation Commission of Canada gives calls to action.[5] These calls to action can improve outcomes for First Nations, Métis, and Inuit peoples in Canada.[5]

Data sovereignty means that each nation governs the collection, ownership, and application of its data. No matter where that data is stored[1-4].

Indigenous: In Canada, Indigenous peoples are First Nations, Métis, and Inuit. These are the original people of the land that is now Canada.[6-9]

First Nations are the original people of the land that is now Canada. First Nations may or may not be Status (registered under the Indian Act) or live in a First Nations community.[10]

Métis have their own history, culture, language, and territory. Métis are people born in the Métis Nation Homeland (Manitoba, Saskatchewan, Alberta, as well as parts of Ontario, British Columbia, and the Northwest Territories).[11]

Inuit are the original people of the Northern region of Canada, Alaska, and Greenland. Their homeland included much of the land, water, and ice in the Artic.[7,12]

1. Canadian Institute for Health Information. (2020). A path forward: toward respectful governance of First Nations, Inuit and Metis Data Housed at CIHI. Accessed April 14, 2022.
2. First Nations Information Governance Centre. The First Nations principles of OCAP®. Accessed April 11, 2022.
3. University of Manitoba Faculty of Health Sciences. (2019). Framework for Research Engagement with First Nation, Metis, and Inuit Peoples. Accessed April 11, 2022.
4. Tagalik S. (2009–2010). National Collaborating Centre for Aboriginal Health. Inuit Qaujimajatuqangit: The Role of Indigenous Knowledge in Supporting Wellness in Inuit Communities in Nunavut. Accessed April 11, 2022.
5. Truth and Reconciliation Commission of Canada. (2015) Truth and Reconciliation Commission of Canada: Calls to Action. Accessed April 11, 2022.
6. Statistics Canada. (2021). Indigenous group of person. Accessed April 11, 2022
7. The Canadian Encyclopedia. Indigenous Peoples in Canada Accessed April 11, 2022
8. United Nations. Indigenous Peoples at the United Nations Accessed April 11, 2022
9. United Nations. United Nations Declaration on the Rights of Indigenous Peoples. 2018
10. Government of Canada. (2014). First Nations People in Canada. Accessed April 11, 2022
11. Libraries and Archives Canada. Government of Canada. (2021) First Nations People in Canada. Accessed April 11, 2022
12. Government of Canada. (2021). Inuit. Accessed April 11, 2022

Status (Registered or Treaty Indian) is a person registered under the Indian Act of Canada. People with status may receive health services under the Non-Insured Health Benefits Program.[1,2]

1. Government of Canada. (2022). About Indian Status. Accessed April 14, 2022
2. Statistics Canada. (2021). Registered or Treaty Indian status of person. Accessed April 11, 2022

A member of an **Inuit land claims agreement** is a beneficiary of: Nunavut Land Claim Agreement, or Inuvialuit Final Agreement.

1. Government of Canada. Your Health Benefits – A Guide for Inuit to Access Non-Insured Health Benefits. Accessed April 22, 2022.

Race and Ethnicity

Race is a term used to classify people into groups usually based on observable physical characteristics (for example, skin colour) but can also include characteristics such as accent or dress.[1,2,3]

Although these differences or groupings are created by the society we live in, racial categories can have significant consequences for people's lives, including unfair and unjust treatment by individuals and institutions. This is what we call racism.

Race should not be confused with ethnicity.

Ethnicity refers to groups of people who share a common culture or ancestry. They may also share a common language, religion, geographic origin, nationality, cultural traditions, migration history, or other commonalites.[1,2]

1. Canadian Institute for Health Information (2022). Guidance on the Use of Standards for Race-Based and Indigenous Identity Data Collection and Health Reporting in Canada Ottawa, ON: CIHI. Accessed April 5, 2022
2. Torontohealthequity.ca. Measuring Health Equity: Demographic Data Collection in Health Care. Accessed April 5, 2022
3. Government of Ontario Anti-Racism Directorate. Data Standards for the Identification and Monitoring of Systemic Racism. Accessed April 5, 2022

Disability

Severe and persistent conditions – long-term conditions or disabilities that affect day-to-day activities. The condition does not have to be diagnosed by a provider.

Difficulty seeing or hearing – does not refer to conditions that can be corrected with aids such as glasses or hearing aids.

Difficulty with communication – refers to a difficulty because of a physical or mental condition and not because of other things such as language barriers or reading or writing level.

Sex at Birth

Sex at Birth is often recorded at a person's birth and is usually based on a person's reproductive system and other physical traits.[1]

Intersex refers to people born with differences in their reproductive features[2,4] that may not be easily characterized as male or female.[5]

1. Statistics Canada, Government of Canada (2021). Sex at birth of person Accessed April 18, 2022
2. Human Rights Campaign (2011). Glossary of Terms - Human Rights Campaign. Accessed April 18, 2022
3. Torontohealthequity.ca. Measuring Health Equity: Demographic Data Collection in Health Care. Accessed April 18, 2022
4. Ucdavis.edu. (2014). LGBTQIA Resource Center Glossary | LGBTQIA Resource Center. Accessed April 18, 2022
5. Ohrc.on.ca. (2014). Appendix B: Glossary for understanding gender identity and expression | Ontario Human Rights Commission (ohrc.on.ca). Accessed April 18, 2022

Gender Identity

Gender Identity is a person's sense of themselves relating to gender, be it male, female, a combination of both, or neither.[1] Gender can be fluid and may change over time.[2]

Gender non-binary describes a person who may experience a gender identity that is neither exclusively woman or man, is a combination of woman and man or is between or beyond genders.[4]
Non-binary can also be used as an umbrella term for identities that are gender fluid, moving between and through identities.[1]

Two-Spirit is a term used by Indigenous peoples to self-describe and express diverse sexual, gender and/or spiritual identities.[5] It is a way for Indigenous peoples to reconnect with their traditional languages, ways, and cultures.[6]

Transgender or trans is an umbrella term for people whose gender identity or expression is different from conventional or cultural expectations based on the sex they were assigned at birth.[7,8]

1. Ohrc.on.ca. (2014). Appendix B: Glossary for understanding gender identity and expression | Ontario Human Rights Commission. Accessed April 18, 2022
2. Torontohealthequity.ca. Measuring Health Equity: Demographic Data Collection in Health Care. Accessed April 18, 2022
3. Statistics Canada, Government of Canada (2021). Sex at birth of person. Accessed April 18, 2022
4. Losty, M. and O'Connor, J. (2017). Falling outside of the 'nice little binary box': a psychoanalytic exploration of the non-binary gender identity. *Psychoanalytic Psychotherapy*, 32(1), pp.40–60
5. Knudson, S. and Hahn, D. (2019). *Committing sociology: critical perspectives on our social world*. Toronto: Pearson Canada.
6. Fewster, P.H. (2018). Researching for LGBTQ Health. Lgbtqhealth.ca. Accessed April 18, 2022
7. Ucdavis.edu. (2014). LGBTQIA Resource Center Glossary | LGBTQIA Resource Center. Accessed April 18, 2022
8. Human Rights Campaign (2011). Glossary of Terms - Human Rights Campaign. Accessed April 18, 2022

Sexual Orientation

Sexual orientation is a term for the emotional, physical, romantic, sexual, and spiritual attraction, non-attraction, desire, or affection for another person.[1]

Aromantic describes a romantic identity for people who do not experience romantic attraction to people of any gender but may still have sexual attraction to other people.[2]

Asexual describes people who do not experience sexual attraction to people of any gender but may still experience romantic attractions to other people.[1,3]

Bisexual describes a person whose primary emotional, romantic, or sexual attraction is toward people of both the same and different gender.

Demisexual describes a person who feels sexual attraction only to people that they have an emotional bond with.[3]

Homosexual or Gay describes a person whose primary emotional, romantic, or sexual attraction is toward people of the same gender.[1]

Heterosexual or Straight describes a person whose primary emotional, romantic, or sexual attraction is toward people of an opposite gender.

Lesbian describes a person, usually a woman, whose primary emotional, romantic, or sexual attraction is toward people of the same gender.[1]

Pansexual describes a person who has emotional, romantic, or sexual desire for people of all genders and sexes.[1]

Queer is a term used to express a range of identities and orientations that are beyond the mainstream.[1]

Two-Spirit is a term used by Indigenous peoples to self-describe and express diverse sexual identities as having both a masculine and feminine spirit.[4] It is a way for Indigenous peoples to reconnect with their traditional languages, ways, and cultures.[5]

1. Human Rights Campaign (2011). Glossary of Terms - Human Rights Campaign. Accessed April 18, 2022
2. Suen, L.W., Lunn, M.R., Katuzny, K., … & Obedin-Maliver, J. (2020). What Sexual and Gender Minority People Want Researchers to Know About Sexual Orientation and Gender Identity Questions: A Qualitative Study. *Archives of Sexual Behavior*, 49(7), pp.2301–2318.
3. Ucdavis.edu. (2014). LGBTQIA Resource Center Glossary | LGBTQIA Resource Center. Accessed April 18, 2022
4. Knudson, S. and Hahn, D. (2019). *Committing sociology: critical perspectives on our social world*. Toronto: Pearson Canada.
5. Fewster, P.H. (2018). Researching for LGBTQ Health. Lgbtqhealth.ca. Accessed April 18, 2022

Social Needs

Education

Some high school means high school education may be in progress or stopped.

Ongoing means schooling or training is in progress.

Basic Needs

Basic Needs are things that a person requires to achieve and maintain physical and mental well-being and include food, water, shelter, and clothing.[1-2]

1. Sarlo C. (2013). Poverty: Where do we draw the line? Accessed April 13, 2022
2. Homeless Hub. (2021). Basic Needs. Accessed April 13, 2022

Medication and Medical Supplies

Medicine includes both prescription and over-the-counter medicines.

Medical supplies are any equipment needed to manage health or disability. For example, needles, glucose monitors, walking aids.

Housing Situation
**Social housing** is housing that is subsidized or partly paid for by a level of government.[1]<br><br>**Subsidized housing** means receiving help paying for housing from the government or a private organization.[2]<br><br>**Rent-geared-to-income** is a type of rent assistance. It lets a person pay a percentage of their income for rent.[3]<br><br>**Supportive housing** refers to housing with on-site staff who provide residents with ongoing support with their needs.<br><br>1. www.homelesshub.ca. Affordable Housing | The Homeless Hub. Accessed April 7, 2022<br>2. Settlement.org. (2018). What is subsidized housing? Accessed April 7, 2022<br>3. www.durham.ca. (2021). Rent-Geared-to-Income Housing (RGI) - Region of Durham . Accessed April 7, 2022
Transportation
**"Medical Appointments"** are appointments to meet your health needs. This may include an appointment with your doctor(s), specialist, or social worker. This also includes getting medicine from your doctor or pharmacist.[1]<br><br>**Lack of transportation** may be because of:[2]<br>• cost and access,<br>• distance,<br>• gas or parking costs,<br>• public transportation safety,<br>• and/or other reasons.<br><br>**"Things needed for daily living"** are things such as groceries or personal products that you need to be healthy.<br><br>1. National Association of Community Health Centre, Inc, Association of Asian Pacific Community Health Organizations, and the Oregon primary Care Association. (2019). PRAPARE. Protocol for Responding to and Assessing Patients' Assets, Risks and Experiences. Implementation and Action Toolkit. Accessed April 14, 2022<br>2. Syed, ST., Gerber, BS., Shapr, LK. (2013). Traveling toward disease" transportation barriers to health care access. J community Health, 38(5), pp. 976-993.
Phone and Internet
**"Consistent access"** means having regular, reliable, and adequate access to a phone or internet to obtain needed services. This includes access to a phone or internet for a healthcare appointment.[1] This also includes access to health information that is on the internet that your provider refers you to.[1]<br><br>1. Graves, JM., Abshire, DA., Amiri, S., Mackelprang, JL. (2021). Disparities in technology and broadband internet access across rurality: implications for health and education. Fam Community Health, 44(4), pp. 257-265.
Basic Utilities

Basic utilities[1] may include:

- heating or cooling
- electricity or hydro
- water or sewer
- phone, and internet.

Basic utilities may vary by where a person lives. For example, homes may be heated using natural gas, furnace oil or wood depending on where the home is.

1. International Policy Centre for Inclusive Growth (2009) Equitable access to basic utilities: Public versus private provision and beyond. Accessed: April 20, 2022.

Social Supports

Having **"people you feel you can open up to or confide in"** means being able to talk to people that you care about, trust and feel close to.[1]

1. National Association of Community Health Centre, Inc, Association of Asian Pacific Community Health Organizations, and the Oregon primary Care Association. (2019). PRAPARE. Protocol for Responding to and Assessing Patients' Assets, Risks and Experiences. Implementation and Action Toolkit. Accessed April 14, 2022

Employment

Health and safety concerns at work may include concerns about unsafe work (for example, lifting items that are too heavy, working with violent clients), unsafe work environments (for example, slippery floors, broken machinery), or harassment due to race, gender, or sexual orientation.[1]

Concerns about **employment rights** may include questions about the right to breaks, sick leave or vacation time.[1]

Health and safety rights[1] include the right to:

- know about unsafe work
- refuse unsafe work
- participate in workplace health and safety decisions

1. Lewchuk, W., 2013. The limits of voice: Are workers afraid to express their health and safety rights. Osgoode Hall LJ, 50(4), p. 789-812. Accessed April 20, 2022

Ethnicity

Ethnicity refers to groups of people who share a common culture or ancestry. They may also share a common language, religion, geographic origin, nationality, cultural traditions, migration history and so on.[1,2]Ethnicity is not the same as race.

Race is a term used to classify people into groups usually based on observable physical characteristics (e.g., skin colour) but can also include characteristics such as accent, dress and so on.[1,2,3]

1. Canadian Institute for Health Information (2022) Guidance on the Use of Standards for Race-Based and Indigenous Identity Data Collection and Health Reporting in Canada Ottawa, ON: CIHI. Accessed April 5, 2022

2. Torontohealthequity.ca. Measuring Health Equity: Demographic Data Collection in Health Care. Accessed April 5, 2022
3. Government of Ontario Anti-Racism Directorate. Data Standards for the Identification and Monitoring of Systemic Racism. Accessed April 5, 2022

Religious Affiliation

"Religious or spiritual affiliation" is about your connection with a particular set of beliefs, attitudes, or practices. This may or may not be related to a specific religion (for example, Catholicism, Islam) or belief in a higher being.[1] This also relates to a person's sense of spirituality.

1. Chatters, L. (2000) 'Religion and Health: Public health research and practice,' Annual Review of Public Health, 21, pp. 335-367. Doi:

Sample Script for Staff

- Answers to common questions and concerns you may hear from patients

SAMPLE SCRIPTS FOR HANDLING PATIENT QUESTIONS

**Patient Question**	**What does this have to do with my care?**
**Script**	"We want to provide care based on our patients' needs. We don't want to make any assumptions about patient needs or who our patients are. For example, when we know a patient doesn't speak English, we will obtain interpreter services."<br>"Having this information gives us an idea of who visits our clinic."<br>"In some cases, depending on the patient and the situation, knowing this information means providing better care. E.g. getting an interpreter for non-English speakers."
**Patient Question**	**"I'm just here for a quick appointment."**
**Script**	"This information is for both now and future visits as well".
**Patient Question**	**This has nothing to do with me - so what if I say (e.g. straight)?**
**Script**	"We ask everyone these questions. Depending on the patient's response, we may be able to refer them to services or talk to them about any needs they have."
**Patient Question**	**Do I have to answer these questions?**
**Script**	"No, it's completely voluntary and you can choose 'Prefer not to answer' to any or all of the questions."
**Patient Question**	**Who will see this information?**
**Script**	"Your provider(s) will see this information, and it will become part of your medical record. Your information is confidential and protected by law, just like all of your other health information."
Question	**How will this information be used?**
**Script**	"Your provider(s) will use this information to help meet your healthcare needs. In addition, gathering this information from all patients allows the clinic to see if there are gaps in care or services across different populations. Learning this tells us if we need to improve the care we give to our patients."<br><br>"With your permission, a research team may access your anonymous data which will be grouped with other patients' data to study the use and value of this survey in our clinic."

Patient-facing Resources

Poster

A poster to raise patient awareness about the SPARK Survey and data collection happening at your clinic.

INTRODUCING THE SPARK TOOL!

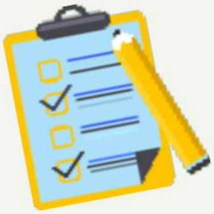

Collecting information about your personal characteristics and social situation

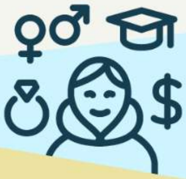

We are collecting information from all our patients using the SPARK Tool!

The SPARK Tool is a survey that will ask about your personal characteristics, such as gender, and your social situation, such as housing.

Why are we asking these questions?

These questions help us understand who you are so that we can give you better care.

It is important to ask these questions to make healthcare for equitable for everyone.

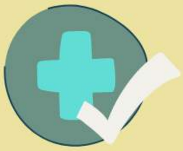

Do I have to answer the questions?

No. The questions are voluntary, and you can choose 'prefer not to answer' to any or all questions. This will not affect your care.

Who will see this information?

This information will be visible to your healthcare team and will be protected like all your other health information.

If used in research, your responses will be made anonymous.

How can I complete the SPARK Tool?

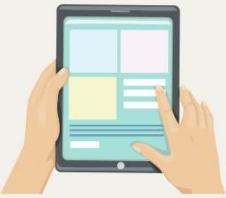

On a tablet in the clinic

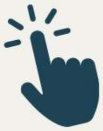

Online through a link sent to your email

?

Questions?
Please contact XXX at XXX or visit XXXX.org

This study has been approved by the St. Michael's Research Ethics Board #21-306

ST. MICHAEL'S
UNITY HEALTH TORONTO

UPSTREAMLAB

Brochure

- To help patients understand why we are asking the sociodemographic questions

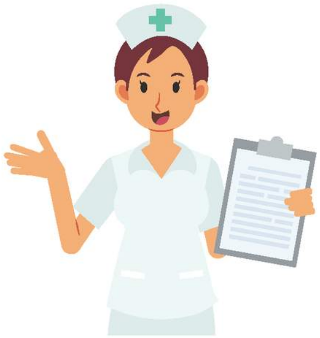

The SPARK Tool

Collecting Information on Personal Characteristics and Social Situations of Patients

Information Brochure

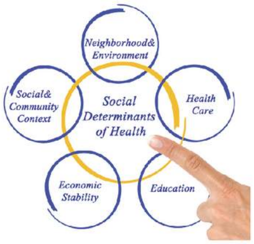

Our clinic has started collecting information on personal characteristics and social situations from our patients using a survey called **the SPARK Tool.**

The SPARK Tool **asks questions** about your **personal characteristics** including:

- age
- language preference
- if born in Canada
- race and ethnicity
- disability
- sex at birth
- gender identity
- sexual orientation

The SPARK Tool **asks questions** about your **social situation** including:

- education level
- ability to pay for basic needs
- access to food
- medication access
- housing situation
- transportation access
- phone and internet access
- access to utilities
- access to social or family supports
- employment status

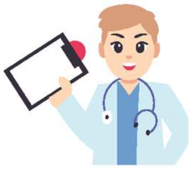

Why are you asking me these questions?

These questions **help us understand who you are** so that we can strive to give you better care.

It is important to ask these questions to **make healthcare more equitable (fair) for everyone.**

Who can see this information?

We take your privacy very seriously.

This **information** will not be visible to anyone outside your healthcare team and **protected like all your other health information.**

If used in research, the information from all patients and clients will be combined, and researchers will not be able to identify who any of the patients and clients are.

How will you use this information?

Your **healthcare provider** may look at and use your information to improve your individual care. The **healthcare clinic** may use it to develop programs and do service training.

Members of your healthcare team may refer you to services, provide you with information, or identify unique needs such as:

- Language translation
- Health and care information
- Treatment programs
- Accommodation for disabilities
- Financial and similar supports related to housing, medication, utilities, and food.

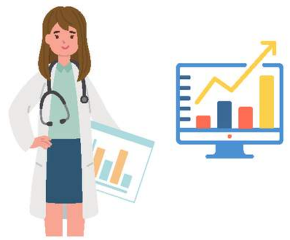

Is it legal to ask these questions?

Yes. **It is legal to ask these questions.** The safe collection of this data is also urged by the **Canadian Public Health Association.**

We believe that we cannot fully understand patients and clients healthcare experiences without knowing more about them.

I'm here for a quick appointment. How is this relevant to my care?

It is important for us know **who we serve** and whether patient and client needs match the care we provide.

This information will help us understand and plan care not only for your future visits but for other patients and clients who may have similar needs as yours.

What if there are survey questions that I don't want to answer on the SPARK Tool?

You can select **"prefer not to answer"** to any or all questions. **This will not impact the care you receive here.**

How can I complete the SPARK Tool?

- On a **tablet** given to you by front desk staff when you come to the clinic

OR

- Online through a **survey link** sent to your email

Questions or Concerns?

The **clinic front desk staff can answer questions** you may have about the SPARK Tool.

If you have questions or concerns about the information you provide in the SPARK Tool or how your healthcare team may use it, you may discuss them with your healthcare provider.

Members of the **SPARK research team** would be happy to discuss the survey and this study with you.

If you have questions, please contact the SPARK research coordinator XXXX

The University of XXXX Ethics Board approves this study. Project ID: XXXX

CONTINUE

Studying the SPARK Tool Implementation

Overview of the SPARK Project: Research Aspect

A research team will be evaluating the success of the SPARK Tool implementation by:

1. Developing this training module, and collecting data about it including staff and provider feedback and number of staff and providers that completed it
2. Collecting data on the number of patients who were offered and completed or declined the SPARK tool and response rate for each question
3. Asking all patients to complete a feedback Tool immediately after completing the SPARK Tool
4. Inviting a small number of patients to participate in an interview on their experience of completing the SPARK Tool
5. Where possible, conducting clinic observations to understand how well the SPARK Tool data collection fits into regular clinic work flows

CONTINUE

Wrap-Up

Takeaway Points

- Sociodemographic data collection is a critical step in improving health equity and health outcomes for patients
- Patients and clients are willing to share this information
- Some patients and clients are likely to have experienced discrimination and harassment and may be reluctant to answer questions
- Front desk staff play a critical role in collecting this data
- The approach and messaging of the person offering the Tool is the best predictor of patient and client engagement
- Embedding data collection into ongoing practices will foster success
- Best Practice: Data collection should include provider follow-up with patient to discuss any identified needs

Additional Training

- A meeting will be held shortly before the Tool is launched at your clinic to review clinic-specific details and to discuss any questions.

Contact Information

If you have any questions about this training or the SPARK implementation please contact the research staff for your site:

Saskatchewan: zita.seshie@usask.ca

Manitoba: Leanne.Kosowan@umanitoba.ca

Ontario: sparkproject@unityhealth.to

Nova Scotia: sparksty@dal.ca

Newfoundland and Labrador: dhowse@mun.ca

Module Completion Form

To evaluate the implementation of the SPARK Tool in your clinic it is helpful for us to know

who completed this training module and whether it is valuable. Please fill out the form below to indicate that you've completed the module.

Sorry, this survey is not currently active.

Powered by Qualtrics

CONTINUE

Help Us Improve this Training Module

We would like to ask you a few questions about your experience with the online training session in a 5 minute follow-up evaluation survey.

Your feedback will help us understand how helpful the online training is and how we can improve it.

Your responses on this evaluation survey will be anonymous and are not linked to the information you provided in the Module Completion Form.

Please follow this link to complete the survey.

CONTINUE

Thank You

Thank you for completing the SPARK implementation online training module.

STROBE Statement—checklist of items that should be included in reports of observational studies

	Item No	Recommendation	Page No
**Title and abstract**	1	(*a*) Indicate the study's design with a commonly used term in the title or the abstract	3
		(*b*) Provide in the abstract an informative and balanced summary of what was done and what was found	3
**Introduction**			
Background/rationale	2	Explain the scientific background and rationale for the investigation being reported	4
Objectives	3	State specific objectives, including any prespecified hypotheses	4-5
**Methods**			
Study design	4	Present key elements of study design early in the paper	5-6
Setting	5	Describe the setting, locations, and relevant dates, including periods of recruitment, exposure, follow-up, and data collection	5-6
Participants	6	(*a*) *Cohort study*—Give the eligibility criteria, and the sources and methods of selection of participants. Describe methods of follow-up<br>*Case-control study*—Give the eligibility criteria, and the sources and methods of case ascertainment and control selection. Give the rationale for the choice of cases and controls<br>*Cross-sectional study*—Give the eligibility criteria, and the sources and methods of selection of participants	5-6
		(*b*) *Cohort study*—For matched studies, give matching criteria and number of exposed and unexposed<br>*Case-control study*—For matched studies, give matching criteria and the number of controls per case	N/A
Variables	7	Clearly define all outcomes, exposures, predictors, potential confounders, and effect modifiers. Give diagnostic criteria, if applicable	5-8
Data sources/ measurement	8*	For each variable of interest, give sources of data and details of methods of assessment (measurement). Describe comparability of assessment methods if there is more than one group	*5-8*
Bias	9	Describe any efforts to address potential sources of bias	5-8,14
Study size	10	Explain how the study size was arrived at	6
Quantitative variables	11	Explain how quantitative variables were handled in the analyses. If applicable, describe which groupings were chosen and why	7-8
Statistical methods	12	(*a*) Describe all statistical methods, including those used to control for confounding	7-8
		(*b*) Describe any methods used to examine subgroups and interactions	7-8
		(*c*) Explain how missing data were addressed	7-8,14
		(*d*) *Cohort study*—If applicable, explain how loss to follow-up was addressed<br>*Case-control study*—If applicable, explain how matching of cases and controls was addressed<br>*Cross-sectional study*—If applicable, describe analytical methods taking account of sampling strategy	N/A
		(*e*) Describe any sensitivity analyses	

**Results**			
Participants	13*	(a) Report numbers of individuals at each stage of study—eg numbers potentially eligible, examined for eligibility, confirmed eligible, included in the study, completing follow-up, and analysed	8-12
		(b) Give reasons for non-participation at each stage	8-12
		(c) Consider use of a flow diagram	Figure 1, Supp Table 1
Descriptive data	14*	(a) Give characteristics of study participants (eg demographic, clinical, social) and information on exposures and potential confounders	8-12
		(b) Indicate number of participants with missing data for each variable of interest	N/A
		(c) *Cohort study*—Summarise follow-up time (eg, average and total amount)	7
Outcome data	15*	*Cohort study*—Report numbers of outcome events or summary measures over time	*8-12*
		Case-control study—Report numbers in each exposure category, or summary measures of exposure	
		Cross-sectional study—Report numbers of outcome events or summary measures	
Main results	16	(*a*) Give unadjusted estimates and, if applicable, confounder-adjusted estimates and their precision (eg, 95% confidence interval). Make clear which confounders were adjusted for and why they were included	N/A
		(*b*) Report category boundaries when continuous variables were categorized	N/A
		(*c*) If relevant, consider translating estimates of relative risk into absolute risk for a meaningful time period	N/A
Other analyses	17	Report other analyses done—eg analyses of subgroups and interactions, and sensitivity analyses	8-12
**Discussion**			
Key results	18	Summarise key results with reference to study objectives	12-14
Limitations	19	Discuss limitations of the study, taking into account sources of potential bias or imprecision. Discuss both direction and magnitude of any potential bias	14
Interpretation	20	Give a cautious overall interpretation of results considering objectives, limitations, multiplicity of analyses, results from similar studies, and other relevant evidence	14-15
Generalisability	21	Discuss the generalisability (external validity) of the study results	14-15
**Other information**			
Funding	22	Give the source of funding and the role of the funders for the present study and, if applicable, for the original study on which the present article is based	1,16

*Give information separately for cases and controls in case-control studies and, if applicable, for exposed and unexposed groups in cohort and cross-sectional studies.

Note: An Explanation and Elaboration article discusses each checklist item and gives methodological background and published examples of transparent reporting. The STROBE checklist is best used in conjunction with this article (freely available on the Web sites of PLoS Medicine at http://www.plosmedicine.org/, Annals of Internal Medicine at http://www.annals.org/, and Epidemiology at http://www.epidem.com/). Information on the STROBE Initiative is available at www.strobe-statement.org.
